# Targeting tryptophan hydroxylase 1 restricts growth and suppresses plasticity in neuroendocrine prostate cancer

**DOI:** 10.1172/JCI200391

**Published:** 2026-09-17

**Authors:** Jing Wei, Jing Wang, Jingrui Chen, Michelle Zhang, Chia-Hui Chen, Tianjie Pu, Alivia O’Brien, Sephtis Hargrove, Eva Corey, Tzu-Ping Lin, Allen C. Gao, Boyang Jason Wu

**Affiliations:** 1 Department of Pharmaceutical Sciences, College of Pharmacy and Pharmaceutical Sciences, Washington State University, Spokane, Washington, USA.; 2 Leigh High School, San Jose, California, USA.; 3 Summer Undergraduate Research Fellowship Program, College of Pharmacy and Pharmaceutical Sciences, Washington State University, Spokane, Washington, USA.; 4 Department of Urology, University of Washington, Seattle, Washington, USA.; 5 Department of Surgery, Kaohsiung Veterans General Hospital, Kaohsiung, Taiwan, China.; 6 Department of Urology, School of Medicine and Shu-Tien Urological Research Center, National Yang Ming Chiao Tung University, Taipei, Taiwan, China.; 7 Department of Urologic Surgery, University of California, Davis, Sacramento, California, USA.

**Keywords:** Cell biology, Oncology, Drug therapy, Oncogenes, Prostate cancer

## Abstract

Advanced prostate cancer has increasingly developed a lethal neuroendocrine form, small cell/neuroendocrine prostate cancer (NEPC), as a consequence of the widespread use of highly potent androgen receptor signaling inhibitors in castration-resistant disease. The molecular mechanisms remain unclear and no effective therapies currently exist. We report that tryptophan hydroxylase 1 (TPH1), the enzyme responsible for peripheral serotonin biosynthesis — a neurotransmitter enriched in neuroendocrine tumors and a classical neuroendocrine biomarker — was upregulated in both de novo and therapy-induced human NEPC. TPH1 upregulation was necessary and sufficient for neuroendocrine differentiation and the NEPC phenotype through its enzymatic activity. Silencing TPH1 suppressed neuroendocrine plasticity and various aggressive behaviors of NEPC cells, including proliferation, invasion, sphere formation, and NEPC tumor xenograft growth. Mechanistically, TPH1 activated mTOR via intracellular serotonin-dependent serotonylation of mTOR at glutamine 2453, which triggered the induction of FOXM1 and E2F1 to drive NEPC differentiation and growth. Importantly, pharmacological inhibition of TPH1 using the clinically available inhibitor LX1606 effectively restricted growth and neuroendocrine marker expression in multiple NEPC cell lines and patient-derived xenografts. Collectively, these findings characterize TPH1’s contribution to NEPC and suggest TPH1 as a potential therapeutic target.

## Abstract 2

Tryptophan hydroxylase 1 is highly expressed in human neuroendocrine prostate cancer and represents a potential therapeutic target.

## Graphical abstract

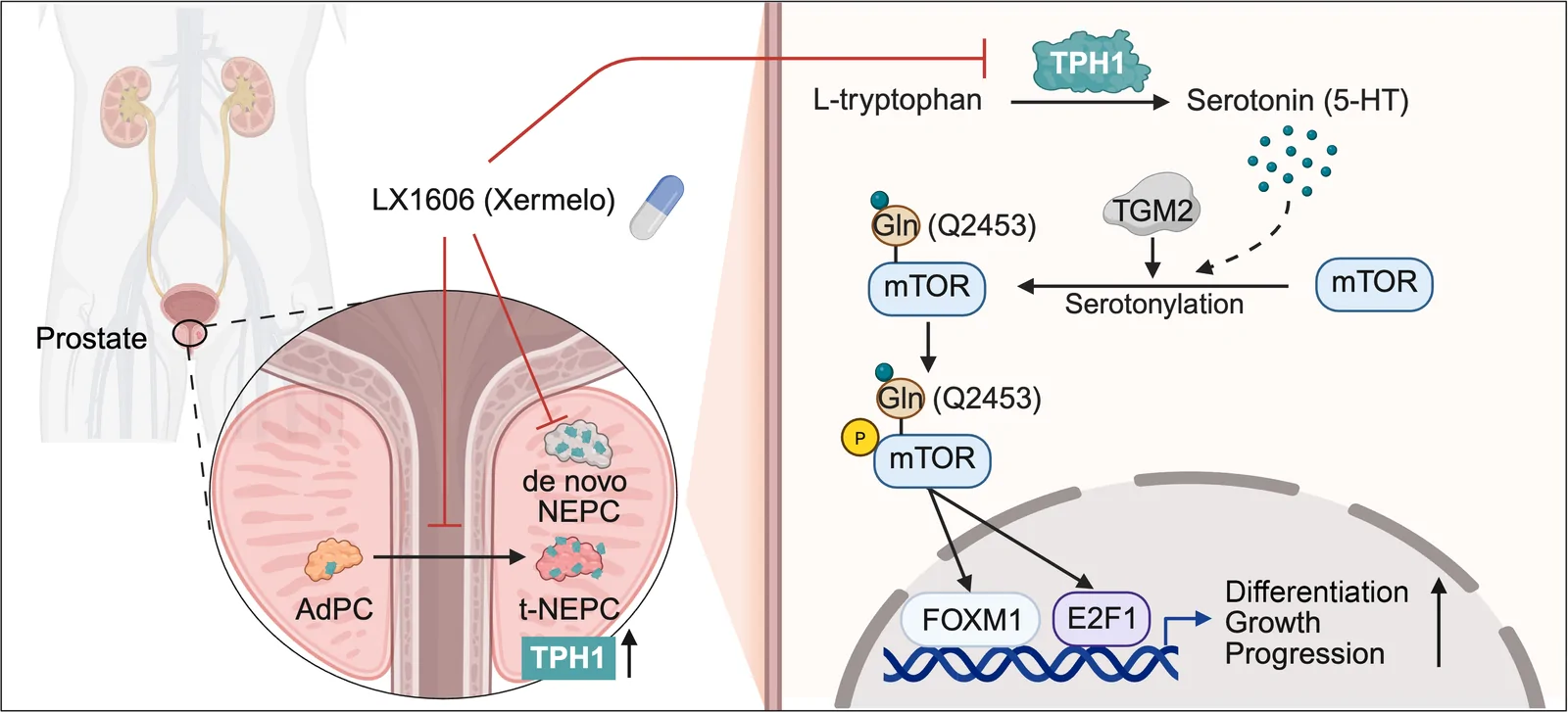

## Introduction

Prostate cancer (PC) is the second most commonly diagnosed cancer and the fifth leading cause of cancer death among men globally (1). Androgen deprivation therapy (ADT) is the cornerstone treatment of PC. Despite an initial favorable response to ADT, the vast majority of tumors relapse as castration-resistant prostate cancer (CRPC), including a subset of CRPC that progresses to small cell or poorly differentiated neuroendocrine prostate cancer (NEPC). With the clinical introduction of highly potent androgen receptor signaling inhibitors (ARSIs), such as enzalutamide (Enz), the incidence of therapy-induced NEPC (t-NEPC) has escalated in recent years. Although it rarely occurs de novo, accounting for less than 2% of all PCs, NEPC has been reported in up to 25% of advanced therapy-resistant CRPC cases (2). NEPC demonstrates high proliferative rates, widespread metastases to viscera and bone, acquisition of stemness, and reduced or lost androgen receptor (AR) signaling during the lineage switch from AR^+^ luminal prostatic carcinoma to a neuroendocrine (NE) phenotype resistant to ADT (3). Currently, NEPC remains incurable, with a median overall survival of less than a year from the time of diagnosis (4), highlighting the urgent need for new effective therapies.

Recent studies have revealed genetic and epigenetic alterations as potential regulators of NE plasticity in PC, including tumor suppressors (e.g., RB1, TP53), transcription factors (e.g., SOX2, ONECUT2, MYCN), and chromatin modifiers (e.g., EZH2) (5). Despite these efforts, the contributing mechanisms of NEPC pathogenesis and progression remain elusive, and importantly, druggable targets that drive NEPC are still largely unidentified.

One hallmark of NE tumors is their high neurosynthesizing and neurosecretory abilities, evidenced by the excessive production and secretion of neural and related substances, including neurotransmitters like serotonin (also known as 5-hydroxytryptamine) (6). As a key neurotransmitter involved in a spectrum of biological and physiological processes in the brain and peripheral tissues, serotonin is highly expressed and secreted in different NE tumors, including NEPC, and is considered a bona fide NE marker (7, 8). Serotonin’s biological functions are mediated through receptor-dependent or -independent mechanisms using extra- or intra-cellular serotonin, respectively (9, 10). Serotonin has been reported to exert a mitogenic effect in several types of cancer (11), including PC (12, 13), but its role and specific mechanisms in NEPC are not yet fully understood. Among the regulators of the serotonergic system, tryptophan hydroxylase 1 (TPH1) catalyzes the first and rate-limiting step in peripheral serotonin biosynthesis from the essential amino acid l-tryptophan (8), making it an ideal target to fully block serotonin effects in cancerous peripheral tissues, including PC. Here, we explore the role, mechanism, and therapeutic targeting potential of TPH1 in NEPC.

## Results

### TPH1 is upregulated in NEPC.

To clarify TPH1’s clinical relevance to NEPC, we examined TPH1 protein expression in human PC tissues. Immunohistochemistry (IHC) revealed higher TPH1 expression in CRPC than in hormone-sensitive PC (HSPC) and normal prostatic glands, with further elevation in CRPC with NE histology (CRPC-NE) compared with the adenocarcinoma counterpart (CRPC-Adeno), where NE differentiation was predefined by positive synaptophysin (SYP) staining (Figure 1A). Within the same CRPC-NE samples, TPH1 protein levels were higher in SYP^+^ NE-differentiated areas compared with SYP^–^ adenocarcinoma foci (Figure 1B). We also demonstrated a positive correlation between TPH1 and the additional NE marker CD56 in these CRPC-NE samples using multiplex immunofluorescence (IF) staining (Supplemental Figure 1A; supplemental material available online with this article; https://doi.org/10.1172/JCI200391DS1). We also observed increased TPH1 protein levels in CRPC and NEPC compared with HSPC, with NEPC exhibiting greater TPH1 levels than CRPC in a LuCaP PC patient-derived xenograft (PDX) tissue microarray (TMA) (14) (Supplemental Figure 1B). Examining clinical datasets, we found increased *TPH1* mRNA levels in NEPC compared with CRPC-Adeno across 3 datasets and in NE^+^ cases compared with others within a dataset categorized by AR and NE activity (Figure 1C). Additionally, *TPH1* showed a positive coexpression correlation with the NE marker *CHGA* across multiple cohorts (Supplemental Figure 1C). We further demonstrated a downregulated androgen-responsive gene set and a strong correlation of genes differentially expressed in human NEPC in *TPH1*-high versus -low tumors in the Beltran cohort (15) (Figure 1D). These data underscore TPH1’s clinical relevance to NEPC.

Next, we analyzed a panel of human PC cell lines and observed substantially higher TPH1 protein levels in AR^–^ de novo small cell/NE cells, LASCPC-01 and NCI-H660, accompanied by the absence of prostate-specific antigen (PSA) and strong expression of NE markers, compared with several AR^+^ prostatic adenocarcinoma (AdPC) cell lines (LNCaP, C4-2, and VCaP). We also demonstrated upregulated TPH1 protein in Enz-resistant C4-2B (C4-2B^ENZR^) cells, which model t-NEPC by loss of canonical AR signaling and acquisition of NE traits (16), compared with control cells (Figure 1E). Based on their differential TPH1 expression (Supplemental Figure 1D), we selected LNCaP, C4-2B^ENZR^, and LASCPC-01 as the main cell line models for TPH1 manipulation. Notably, the brain-specific isoform TPH2 was undetected in all PC cell lines in contrast with its high level in mouse brain tissue (Supplemental Figure 1E). In parallel with the changes in TPH1 expression, we also found elevated serotonin levels in NEPC cells compared with AdPC cells (Supplemental Figure 1F), which was corroborated in selected tumor xenografts (Supplemental Figure 1G). We further examined TPH1 expression in another NEPC model characterized by dual ablation of *RB1* and *TP53* (17, 18). We found increased TPH1 expression in Rb1/p53 double-knockdown (DKD) LNCaP cells, as well as in NE-featured tumors from Pten/Rb1 double-knockout and Pten/Rb1/Trp53 triple-knockout genetically engineered mouse models (GEMMs) (Supplemental Figure 1, H and I). Furthermore, we demonstrated concurrent upregulation of *TPH1* and NE markers by AR inactivation under culture in androgen-depleted media or Enz treatment, or by REST silencing, a neural transcription factor lost during progression to NEPC (19), in LNCaP cells (Supplemental Figure 1J). The effect of AR on TPH1 was also reflected in decreased *TPH1*, along with induced *KLK3* and reduced NE markers in LNCaP cells after androgen stimulation with R1881 (Supplemental Figure 1K). Consistently, we also showed increased *TPH1* mRNA levels in post-Enz relative to pre-Enz prostate tumors collected from the same individuals before and after 3 months of Enz treatment (Supplemental Figure 1L). Additionally, GSEA revealed that multiple gene sets related to key steps and regulation of the neurotransmitter system, including biosynthesis, transport, and secretion of neurotransmitters, were prevalently upregulated in the C4-2B^ENZR^ and NEPC patient samples compared with respective controls (Supplemental Figure 1M). Collectively, these results indicate that TPH1 is upregulated in NEPC.

### TPH1 induces and maintains NEPC differentiation and growth.

To determine TPH1 function in PC, we stably overexpressed wild-type (WT) or enzymatically inactive mutant (A300T) (20) TPH1 in TPH1-low LNCaP AdPC cells. WT TPH1 overexpression (OE) markedly increased serotonin secretion and cell proliferation compared with the control expressing an empty vector, while mutant TPH1 failed to do so (Figure 2, A–C). WT but not mutant TPH1 was also sufficient to facilitate the transition from a luminal to a NE, basal, mesenchymal, and stem-like mixed phenotype by inducing corresponding markers concomitant with downregulation of luminal markers, including AR and PSA, in LNCaP cells (Figure 2A). These changes were reversed by siRNA-mediated KD of TPH1 (Supplemental Figure 2, A and B). Gain of NE and other nonluminal lineage features freed LNCaP cells expressing WT but not mutant TPH1 from reliance on AR for increased proliferation in androgen-depleted media or under Enz treatment (Figure 2D). WT but not mutant TPH1 OE further resulted in an NE-like differentiated cell morphology (21) with increases in neurite number and length (Figure 2E). Moreover, we showed that OE of WT but not mutant TPH1 induced cell invasion and sphere formation, indicating enhanced metastatic potential and stemness, respectively (Figure 2, F and G). Additionally, we observed similar effects on lineage markers, cell morphology, and AR-independent proliferation upon TPH1 OE in C4-2 AdPC cells (Supplemental Figure 2, C–E). These results demonstrate that TPH1 promotes NE differentiation and aggressive behavior in AdPC cells through its enzymatic activity.

Conversely, we stably knocked down TPH1 expression in C4-2B^ENZR^ and LASCPC-01 cells, representing therapy-induced and de novo NEPC, respectively, using 2 independent shRNAs, which led to decreased serotonin secretion and proliferation (Figure 3, A–C, and Supplemental Figure 3A). We demonstrated that TPH1 knockdown (KD) slowed cell cycle progression but had minimal effects on cell apoptosis and senescence in these NEPC cells (Supplemental Figure 3, B–D). TPH1 KD also reduced NE, basal, stem cell, and mesenchymal markers in both cell lines (Figure 3A). GSEA of genes differentially expressed in TPH1-KD cells revealed repressed transcriptomic profiles compared with those in human NEPC (Figure 3D). Notably, re-expression of TPH1 in TPH1-KD cells restored proliferation and expression of NE and other nonluminal lineage markers (Supplemental Figure 4, A and B). TPH1 silencing also markedly impaired the invasion and sphere formation of NEPC cells (Figure 3, E and F, and Supplemental Figure 4C). Moreover, we observed reductions in serotonin secretion, NE marker expression, and proliferation of NCI-H660 cells after silencing TPH1 with 2 siRNAs (Supplemental Figure 4, D–F). TPH1 KD also decreased cell proliferation and NE marker expression and induced dedifferentiated cell morphology in Rb1/p53 DKD LNCaP cells (Supplemental Figure 4, G–I). Then, using organoids derived from TPH1-high NEPC PDX tumors (LuCaP 49, 93, and 173.1) (22) (Supplemental Figure 5A), we demonstrated notable reductions in organoid growth and NE markers following effective *TPH1* KD (Figure 4, A and B, and Supplemental Figure 5, B and C).

To analyze the consequences of TPH1 silencing in vivo, we subcutaneously grafted control and TPH1-KD LASCPC-01 cells into immunodeficient male mice. TPH1 silencing reduced tumor growth rate, tumor weight, and tumor serotonin levels (Figure 4, C–F). IHC confirmed sustained lower TPH1 protein expression and revealed a 78% average drop in the percentage of Ki-67^+^ cells, reflecting decreased cell proliferation, as well as reductions in the NE markers SYP and CD56 in TPH1-KD tumors (Figure 4G). We also observed fewer formed tumors, slower tumor growth, and reduced tumor NE markers after TPH1 depletion in C4-2B^ENZR^ xenografts (Supplemental Figure 5, D–G). Together, these results indicate that TPH1 is both sufficient and necessary for NEPC differentiation and growth.

### TPH1 activates mTOR to operate in NEPC through intracellular serotonin.

Next, we investigated how TPH1 promotes NEPC growth and NE traits. GSEA identified several upregulated Hallmark gene signatures in TPH1-OE LNCaP cells (Figure 5A). Among the top 5 signatures, epithelial-mesenchymal transition (EMT), hypoxia, mTORC1 signaling, and cholesterol homeostasis have been implicated in NEPC (23–26). Given serotonin’s known effect on mTOR activity and mTOR’s central role in mTORC1 signaling (25, 27), we focused on mTOR as a potential mediator of TPH1 function. WT but not mutant TPH1 OE increased phosphorylation of mTOR and 2 mTORC1 downstream effectors, p70S6K and 4E-BP1, in LNCaP cells, suggesting that TPH1 activates mTOR through its enzymatic function, whereas TPH1 KD decreased their phosphorylation in C4-2B^ENZR^ and LASCPC-01 cells. Interestingly, AKT phosphorylation, a known upstream inducer of mTOR, remained stable (Figure 5B and Supplemental Figure 6A). Moreover, re-expressing TPH1 in TPH1-KD cells restored phosphorylation of mTOR, p70S6K, and 4E-BP1 to control levels (Supplemental Figure 6B). Given the reciprocal regulatory circuit between mTOR and AMPK signaling in the maintenance of cellular energy homeostasis (28), we also asked whether TPH1 affects AMPK signaling and found no change in AMPK phosphorylation in TPH1-OE or -KD PC cells (Supplemental Figure 6C). Functionally, we showed that rapamycin inhibition of mTOR reversed TPH1-induced proliferation and NE marker expression in LNCaP cells, with stronger effects in TPH1-OE cells (Figure 5, C and D), suggesting that the reversal is TPH1 dependent rather than a general inhibitory effect. Conversely, we stably expressed a doxycycline-inducible (Dox-inducible) *TPH1* shRNA in C4-2B^ENZR^ and LASCPC-01 cells, and we demonstrated that rapamycin pretreatment abolished the decreases in proliferation and NE markers caused by Dox-induced TPH1 KD in these cells (Figure 5, E and F, and Supplemental Figure 6, D and E). We further showed that silencing Raptor, but not Rictor, repressed TPH1-induced proliferation and NE markers in LNCaP cells, implicating mTORC1 but not mTORC2 as the predominant mediator of TPH1 effects (Supplemental Figure 6, F and G). Consistently, TPH1 silencing in TRAMP-C2 mouse PC cells, derived from a TRAMP transgenic mouse model of NEPC (29), decreased serotonin secretion and downregulated mTOR, p70S6K, and 4E-BP1 phosphorylation, as well as NE markers (Supplemental Figure 7, A and B). These data suggest that mTOR mediates TPH1 function in NEPC cells.

We then examined whether extracellular versus intracellular serotonin mediates TPH1’s effects, including mTOR activation, in NEPC. TPH1 controls the synthesis of intracellular serotonin, which forms covalent bonds with glutamine (Gln) residues on substrate proteins via transamidation, primarily mediated by transglutaminase 2 (TGM2), to modulate their activities, a serotonin receptor–independent (5-HTR–independent) posttranslational modification known as serotonylation (30). Extracellular serotonin released from cells binds to cell surface 5-HTRs to trigger downstream signaling, a mechanism reported to activate mTOR via 5-HTR6 (31). To discern the role of extracellular versus intracellular serotonin in mediating TPH1 function, we treated TPH1-OE LNCaP cells with asenapine, a broad-spectrum 5-HTR antagonist, or sertraline, an inhibitor of serotonin transporter (SERT) for cellular reuptake of released extracellular serotonin. Neither treatment substantially affected TPH1-induced proliferation or NE markers, unlike TPH1 silencing in parallel (Figure 5, G and H). Conversely, exogenous serotonin failed to rescue the reduced proliferation, NE markers, and mTOR phosphorylation caused by TPH1 KD in C4-2B^ENZR^ and LASCPC-01 cells (Supplemental Figure 8, A–C). This aligns with the minimal changes in AKT phosphorylation following TPH1 manipulation, suggesting that TPH1 activates mTOR through a mechanism that likely differs from the canonical AKT-dependent pathway downstream of extracellular serotonin and 5-HTRs (13). These data suggest that TPH1 tends to activate mTOR for NEPC promotion independently of extracellular serotonin.

To explore whether intracellular serotonin mediates TPH1-dependent mTOR activation, we first confirmed increased intracellular serotonin around the nucleus as reported (32) in TPH1-OE LNCaP cells (Figure 5I). Blockade of TGM2-mediated serotonylation with monodansylcadaverine (MDC), but not 5-HTR or SERT inhibition, reduced TPH1-induced phosphorylation of mTOR, p70S6K, and 4E-BP1 in LNCaP cells, suggesting intracellular serotonin’s contribution to mTOR activation in the TPH1 context (Figure 5J). Alternatively, we treated LNCaP cells with 5-hydroxytryptophan (5-HTP), the immediate serotonin precursor converted from l-tryptophan by TPH1, which increases intracellular serotonin by bypassing the rate-limiting biosynthesis step (33). Unlike serotonin, which is primarily transported into cells via SERT (34), 5-HTP enters cells efficiently via facilitated or passive diffusion (35). 5-HTP induced mTOR activation, proliferation, and NE markers in LNCaP cells (Supplemental Figure 8, D and E). In contrast, exogenous serotonin increased proliferation and NE marker expression in LNCaP cells only when SERT was forcibly expressed (Supplemental Figure 8, F–H). Similarly, 5-HTP rescued proliferation that was repressed by TPH1 KD in C4-2B^ENZR^ and LASCPC-01 cells, whereas serotonin required concurrent forced SERT expression to do so (Supplemental Figure 8I). Collectively, these results suggest that TPH1 activates mTOR through TPH1-dependent intracellular serotonin to operate in NEPC.

In addition to the mTOR-targeted mechanism identified by bioinformatic analysis, we examined whether estrogen signaling contributes to TPH1 function in NEPC, given its reported regulation of tryptophan hydroxylase and serotonin synthesis in the brain (36) and its upregulation during PC progression, including NEPC, particularly via ERα (37, 38). Although 17β-estradiol (E_2_) levels were elevated in NEPC cells alongside TPH1 and serotonin (Supplemental Figure 9A), E_2_ treatment neither affected TPH1 and NE marker levels and proliferation in LNCaP cells nor restored the reduced NE markers and proliferation caused by TPH1 KD in NEPC cells (Supplemental Figure 9, B–F). Conversely, blockade of estrogen signaling with the aromatase inhibitor letrozole or the ER antagonist fulvestrant failed to alter *TPH1* and NE marker expression and proliferation of C4-2B^ENZR^ cells, nor did it repress TPH1-induced proliferation of LNCaP cells (Supplemental Figure 9, G–I). As parallel controls, E_2_ increased expression of estrogen-responsive genes and proliferation, whereas letrozole and fulvestrant downregulated E_2_ secretion and ERα protein expression, respectively, in MCF-7 cells (Supplemental Figure 9, J–M). Consistently, siRNA-mediated silencing of individual ERs produced minimal changes in TPH1 and SYP protein expression and proliferation in C4-2B^ENZR^ cells (Supplemental Figure 9, N and O). Additionally, depleting ERα or ERβ did not reverse TPH1-induced cell proliferation and NE markers in LNCaP cells (Supplemental Figure 9, P and Q). These results suggest potential tissue context–dependent estrogen regulation of tryptophan hydroxylases and, further, a low likelihood that TPH1 functions in NEPC through estrogen signaling in our models.

### TPH1 triggers mTOR serotonylation at Gln2453 for activation.

Investigating whether TPH1 regulates mTOR through serotonylation, we found elevated mTOR serotonylation in TPH1-OE LNCaP cells, which was reversed by the TGM2 inhibitor MDC (Figure 6A). In situ proximity ligation assays further revealed a 3-fold increase in mTOR-TGM2 interaction in TPH1-OE LNCaP cells and a 36% reduction in TPH1-KD C4-2B^ENZR^ cells compared with respective controls (Figure 6B and Supplemental Figure 10A). To map the mTOR region for TGM2 binding, which may contain key Gln residue(s) involved in mTOR serotonylation, we cloned full-length (FL) mTOR and 2 truncation mutants, including only the kinase domain or lacking the kinase domain (ΔKD) due to its critical role in mTOR’s activity (Figure 6C). Co-immunoprecipitation (co-IP) assays indicated that the kinase domain (aa 2,181–2,516) (39) was sufficient and necessary for mTOR to interact with TGM2 (Figure 6D). Reciprocally, we generated FL TGM2 and 2 mutant versions, one containing only the catalytic core domain and another lacking this domain (ΔCCD), based on its importance for enzyme activity (Figure 6E). Co-IP assays revealed that the catalytic domain is required for TGM2 to interact with mTOR (Figure 6F). These data suggest that TPH1 enables serotonylation of mTOR, likely via specific Gln residue(s) within the kinase domain.

Given that TGMs preferentially modify glutamines in unstructured regions like flexible loops (40), we examined a reported 55-residue unstructured segment (aa 2,437–2,491) within the mTOR kinase domain (41) and identified Gln2453 as its only Gln residue. Notably, deleting the bulk of this segment (aa 2,443–2,486) was shown to deactivate mTOR (41). We also found that the Gln2453 and flanking sequences are highly conserved across different species (Figure 7A). In vitro TGM2 monoaminylation assays using recombinant truncated mTOR protein (aa 2,226–2,488) demonstrated that mTOR exhibited TGM2-dependent transamidation of MDC, which was attenuated by the TGM2 inhibitor cystamine and donor competition with serotonin, with recombinant fibrinogen protein as a positive control (42) (Figure 7B). Targeted liquid chromatography-tandem mass spectrometry (LC-MS/MS) following TGM2 assays using truncated mTOR protein with MDC further revealed Gln2453, but not Gln2282, which resides outside the unstructured region and thus served as a control, as a reactive substrate for serotonylation (Figure 7C). This was confirmed using a synthesized Gln2453-centered peptide, ^2446^TDSYSAGQSVEILDG^2460^, with MDC or serotonin (Supplemental Figure 10, B and C). These data indicate that mTOR can be serotonylated at Gln2453.

To assess whether mTOR serotonylation impacts its activity, we demonstrated that WT but not catalytically dead mutant (W241A) (43) TGM2 enhanced mTOR phosphorylation in TPH1-OE LNCaP cells (Supplemental Figure 10D), whereas treatment with *TGM2* siRNA or different inhibitors of TGM2, including MDC, cystamine, and LDN27219, reduced mTOR phosphoprotein in C4-2B^ENZR^ and LASCPC-01 cells (Supplemental Figure 10E). Importantly, we observed markedly reduced phosphorylation of mTOR, p70S6K, and 4E-BP1 in LASCPC-01 cells expressing the mutant mTOR (Q2453A), in which Gln2453 was replaced by alanine, compared with WT mTOR (Figure 7D), suggesting that Gln2453 influences mTOR activity. We further examined Rheb binding to mTOR, a critical step in allosteric activation of mTOR for mTORC1 activity (44). MDC treatment reduced the Rheb-mTOR interaction in both C4-2B^ENZR^ and 5-HTP–stimulated LNCaP cells as shown by co-IP and proximity ligation assays (Supplemental Figure 11, A and B). Similarly, the Q2453A mutation decreased the interaction between endogenous Rheb and transfected mTOR in C4-2B^ENZR^ and LASCPC-01 cells (Supplemental Figure 11, C and D). Structural modeling further revealed that Q2453A disrupts polar hydrogen-bond interactions between the side-chain amide group of Gln2453 and neighboring Gly2452/Ser2454 due to the shortened side chain and loss of hydrogen-bonding capacity, resulting in structural instability in this region (Supplemental Figure 11E). This may weaken mTOR’s interaction with other partners, such as Rheb, and warrants further investigation. Additionally, WT but not mutant mTOR rescued proliferation and NE marker expression reduced by TPH1 KD in LASCPC-01 cells, with restored mTOR activity compared with the control (Figure 7, E and F). Together, these results demonstrate that TPH1 induces mTOR serotonylation at Gln2453 to facilitate mTOR activation and mediate TPH1’s effect in NEPC cells.

### TPH1 induces FOXM1 and E2F1 downstream of mTOR as TPH1 effectors.

We carried out pathway analysis of RNA-seq data from TPH1-KD versus control C4-2B^ENZR^ cells and revealed FOXM1 as the most significantly downregulated (absolute value of *z*-score of ≥2 and *P* < 0.05) upstream regulator of the transcriptional changes (Figure 8A). FOXM1 is a master regulator of NEPC that interacts with NE differentiation factors and activates NEPC drivers, such as AURKA, which stabilize N-Myc to promote NEPC (45, 46). Promoter analysis of genes differentially expressed in TPH1-KD C4-2B^ENZR^ cells showed enrichment of E2F-binding sites (Figure 8B). E2F1 exhibits excessively high transcriptional activity in NEPC and confers NEPC development by transactivation of NE differentiation genes (47). Consistent with these findings, we demonstrated that FOXM1, E2F1, AURKA, and N-Myc protein levels were increased in WT but not mutant TPH1-OE LNCaP cells and reduced in TPH1-KD C4-2B^ENZR^ and LASCPC-01 cells (Figure 8C and Supplemental Figure 12A). We also found downregulated FOXM1 and E2F-directed gene signatures in TPH1-KD cells (Figure 8D).

To determine whether FOXM1 and E2F1 mediate TPH1’s effects in NEPC, we showed that FOXM1 or E2F1 silencing more effectively reduced proliferation and NE markers in TPH1-OE LNCaP cells than in control cells (Figure 8, E and F), indicating TPH1-dependent inhibitory effects. Conversely, preincubation with siRNAs against FOXM1 or E2F1 lessened the reductions in proliferation and NE markers caused by Dox-induced TPH1 KD in C4-2B^ENZR^ cells (Supplemental Figure 12, B and C). Moreover, rapamycin suppressed TPH1-induced expression of *FOXM1*, *E2F1*, *AURKA*, and *MYCN* in LNCaP cells (Figure 8G), whereas forced expression of WT but not mutant mTOR restored their expression following TPH1 KD in LASCPC-01 cells (Figure 8H). These data suggest that FOXM1, E2F1, and associated NEPC driver genes act downstream of mTOR in TPH1’s context. Additionally, we found that *TPH1* tends to positively correlate with the mTOR score, defined by Hallmark mTORC1 signaling genes, as well as with *FOXM1*, *E2F1*, *AURKA*, and *MYCN* expression in the Beltran 2016 cohort (Figure 8I). Together, these data demonstrate FOXM1 and E2F1 as valid effectors downstream of mTOR mediating TPH1’s effects on NEPC.

### Pharmacological TPH1 inhibition restricts NEPC differentiation and growth in vitro and in vivo.

Next, we evaluated the efficacy of TPH1 inhibitors, specifically LX1606 (telotristat ethyl, Xermelo), an FDA-approved small-molecule tryptophan hydroxylase inhibitor used clinically to treat carcinoid syndrome diarrhea (48). Due to its inability to cross the blood-brain barrier and interfere with brain-specific TPH2, LX1606 selectively inhibits peripheral TPH1 and avoids adverse effects on the central nervous system (8).

We first assessed the specificity of LX1606 for TPH1. We showed that TPH1-high NEPC cells (LASCPC-01 and NCI-H660) are more responsive to LX1606, with up to 80% lower IC_50_ values than TPH1-low AdPC cells (LNCaP and C4-2) (Supplemental Figure 13A). Effective CRISPR/Cas9-mediated pooled TPH1 knockout in C4-2B^ENZR^ cells also abolished the inhibitory effects of LX1606 on proliferation and NE markers compared with control cells (Supplemental Figure 13, B–D), suggesting TPH1-specific targeting. LX1606 treatment at 2 and 5 μM, which inactivated TPH1 enzymatic activity by up to ~70% and decreased serotonin secretion, dose-dependently repressed proliferation, colony formation, invasion, and sphere formation in C4-2B^ENZR^ and LASCPC-01 cells (Figure 9, A–C, and Supplemental Figure 13, E–J). LX1606 also dose-dependently reduced protein levels of NE markers and TPH1 downstream effectors, including p-mTOR, FOXM1, and E2F1 (Figure 9D and Supplemental Figure 13K). Additionally, LX1606 enhanced the efficacy of cisplatin and carboplatin, 2 platinum-based agents currently used as standard NEPC therapies (49), reducing their IC_50_ values by 3.6- and 1.9-fold, respectively, in LASCPC-01 cells (Supplemental Figure 14).

To address whether LX1606 inhibition of NEPC differentiation and growth in vitro translates in vivo, we established multiple TPH1-high NEPC xenograft mouse models. In mice bearing LASCPC-01 and NCI-H660 subcutaneous xenografts, LX1606 treatment slowed tumor growth and reduced endpoint tumor size and weight compared with vehicle controls (Figure 9, E–G, and Supplemental Figure 15, A–C). Similarly, LX1606 suppressed tumor growth in 3 NEPC PDX models (LuCaP 49, 93, and 173.1), resulting in smaller tumors and lower endpoint weights (Figure 9, H–J, and Supplemental Figure 15, D–I). LX1606 reduced tumor serotonin levels across all NEPC models (Figure 9K and Supplemental Figure 15J), confirming inhibition of tumor TPH1 activity, and decreased Ki-67^+^ cell percentages and NE marker expression (Figure 9L and Supplemental Figure 15K). LX1606 did not alter mouse body weight or cause apparent histopathological abnormalities in the liver or kidneys (Supplemental Figure 16, A and B). Additionally, we observed no substantial changes in tumor growth profiles, along with low and stable tumor serotonin levels, in parallel TPH1-low LNCaP AdPC xenografts (Supplemental Figure 17, A–E).

To further evaluate LX1606’s efficacy in t-NEPC, we found that LX1606 alone increased AR and PSA protein expression in C4-2B^ENZR^ cells, and its combination with Enz dramatically reduced their expression (Supplemental Figure 18A). Consistently, LX1606 restored Enz sensitivity, reducing Enz IC_50_ by nearly 2-fold and colony formation by 67% (Supplemental Figure 18, B and C). In C4-2B^ENZR^ subcutaneous xenografts, LX1606 alone reduced tumor growth and, importantly, resensitized resistant tumors to Enz, with the combination producing the greatest tumor suppression (Supplemental Figure 18, D–F). IHC revealed lower Ki-67^+^ cell percentages and CD56 expression in LX1606-treated tumors, with further decreases following combination treatment. Consistent with in vitro observations, LX1606 alone elevated AR and PSA expression, whereas combined treatment markedly reduced their expression in tumors (Supplemental Figure 18G). Revisiting LX1606-treated LASCPC-01 and LuCaP 49 NEPC tumors, we also demonstrated increased AR and PSA expression in treated tumors (Supplemental Figure 18H). Collectively, these results suggest that TPH1 inhibitors like LX1606 have strong potential to treat NEPC, including t-NEPC, both as monotherapy and in combination with existing therapies, such as ARSIs.

## Discussion

In this study, we identified TPH1 as a key driver of NEPC and TPH1 inhibitors as potential therapeutics. These findings stem from the recognized activated serotonergic system in NEPC and the broader neurosynthesizing and neurosecretory features of NE tumors. We demonstrated strong association of TPH1 expression with a NEPC phenotype in experimental cell lines, GEMMs, and clinical samples, representing both de novo and therapy-induced NEPC. Consistent with our findings, recent scRNA-seq analysis of PC and CRPC specimens revealed that TPH1-expressing epithelial cells tend to cluster toward small cell/NEPC along evolutionary trajectories (50), suggesting TPH1’s potential as a predictor for NEPC. Although not specific to NEPC, some of our findings, such as TPH1’s procancer role, align with previous reports in PC (51) or other cancers (52, 53). Given the shared clinical implications and roles of serotonin, the principal downstream effector of TPH1, in NE tumors, our findings may also shed light on NE malignancies, such as small cell lung and pancreatic cancers. Together, our findings highlight TPH1 as a crucial operator and a promising therapeutic target in NEPC.

A salient mechanistic finding of our study is that TPH1 appears to promote NEPC through intracellular serotonin-dependent activation of mTOR. We identified serotonylation at Gln2453, a highly conserved residue, as a posttranslational mechanism regulating mTOR activity. Our findings show that Gln2453 serotonylation is linked to mTOR activation and downstream effectors, FOXM1 and E2F1, accompanied by enhanced NEPC differentiation and growth. Intriguingly, forced expression of WT but not Q2453A mutant mTOR rescued the proliferation of TPH1-KD LASCPC-01 cells, which lack serotonin, with the extent of proliferation correlated with mTOR phosphoprotein levels across groups. One possible explanation for the residual activity of WT mTOR under serotonin-deficient conditions could be that Gln2453 might undergo monoaminylation by other biogenic monoamines secondary to serotonin, such as histamine, which is produced in prostatic NE cells (54), thereby modulating mTOR activity. Nevertheless, additional studies are needed to establish a comprehensive understanding of mTOR regulation by protein monoaminylation. Additionally, serotonylation and potentially other forms of monoaminylation may represent an underappreciated mechanism for NEPC differentiation and growth and a potential therapeutic vulnerability, especially given the inverse relationship between TGM2, the primary enzyme catalyzing monoaminylation, and AR expression (55), which could exacerbate monoaminylation in NEPC more than in AdPC cells.

Although we demonstrate the function, mechanism, and therapeutic targeting potential of TPH1 in NEPC, we acknowledge the limitations of our study. First, we focused our mechanistic pursuit on mTOR signaling within the TPH1 context based on unbiased RNA-seq data. However, since serotonylation has been shown to occur on various proteins, such as histone H3 and GAPDH (56, 57), we cannot exclude the possibility that other serotonylated proteins may also be linked to TPH1 function in NEPC. Exploring additional serotonylatable protein effectors involved in NEPC with LC-MS/MS merits further investigation and could offer deeper mechanistic insights into TPH1’s role in NEPC. Second, recent studies have shown that tumor- or periphery-derived serotonin contributes to immune regulation in several cancer types, including NE cancers, which can be influenced by tumor or tumor microenvironmental TPH1 levels (58, 59). Despite these advances, it remains unclear whether and how tumor-intrinsic TPH1 influences the immune response in NEPC tumors. Further efforts using immunocompetent NEPC mouse models are required to examine TPH1’s role and potential as a therapeutic target, especially when combined with immune checkpoint blockade.

## Methods

### Sex as a biological variable.

Our study exclusively examined male mice because the disease modeled is relevant in males only.

### Clinical specimens.

The tissue specimens for evaluation of TPH1 expression and its association with NE markers in Figure 1, A and B, and Supplemental Figure 1A were obtained from the Biobank of Taipei General Veterans Hospital, as reviewed and approved by the Institutional Review Board (IRB) of Taipei General Veterans Hospital with written informed consent provided by patients. The LuCaP PDX TMA for TPH1 evaluation in Supplemental Figure 1B was obtained from the University of Washington site of the Prostate Cancer Biorepository Network, containing 38 cases of PDXs with each case arrayed in triplicate.

### Cell lines and cell culture.

The human PC LNCaP, VCaP, LASCPC-01, and NCI-H660, HEK293T, human breast cancer MCF-7, and mouse PC TRAMP-C2 cell lines were obtained from the American Type Culture Collection. The human PC C4-2 cell line was obtained from Leland W.K. Chung (Cedars-Sinai Medical Center, Los Angeles, California, USA). The Enz-resistant C4-2B cell line, C4-2B^ENZR^, was generated as described previously (60). All human cell lines were authenticated by short tandem repeat profiling, tested regularly for mycoplasma using the MycoProbe Mycoplasma Detection Kit (R&D Systems), and used with a number of cell passages below 10. LNCaP and C4-2 cells were cultured in RPMI-1640 medium (Corning) supplemented with 10% fetal bovine serum (FBS; Atlanta Biologicals) and 1% penicillin/streptomycin (Corning). Control and TPH1-OE LNCaP and C4-2 cells were cultured in tryptophan-free medium for 24 hours and then treated with 250 μM tryptophan before subsequent analyses, unless otherwise noted. VCaP, HEK293T, and MCF-7 cells were cultured in DMEM (Corning) supplemented with 10% FBS and 1% penicillin/streptomycin. C4-2B^ENZR^ cells were cultured further in the continuous presence of 20 μM Enz. LASCPC-01 and NCI-H660 cells were cultured in RPMI-1640 medium supplemented with 5% FBS, 1% penicillin/streptomycin, 5 μg/mL insulin (Sigma-Aldrich), 0.01 mg/mL transferrin (Sigma-Aldrich), 30 nM sodium selenite (Sigma-Aldrich), 10 nM hydrocortisone (Sigma-Aldrich), 10 nM β-estradiol (Sigma-Aldrich), and 4 mM l-glutamine (Corning). TRAMP-C2 cells were cultured in DMEM supplemented with 5% FBS, 5% Nu-Serum (Corning), 1% penicillin/streptomycin, 5 μg/mL insulin, and 10 nM R1881 (Sigma-Aldrich).

### Biochemical analyses.

Total RNA from PC and organoid cultures was isolated using a RNeasy Mini Kit (QIAGEN) and reverse-transcribed to cDNA by M-MLV reverse transcriptase (Promega) following the manufacturers’ instructions. qPCR was conducted using SYBR Green PCR Master Mix and run with the Applied Biosystems QuantStudio 3 Real-Time PCR System (Thermo Fisher Scientific). PCR conditions included an initial denaturation step of 10 minutes at 95°C, followed by 40 cycles consisting of 15 seconds at 95°C and 1 minute at 60°C. The PCR data were analyzed by the 2^-ΔΔCT^ method. Primer sequences for qPCR are provided in Supplemental Table 1. For immunoblots, cells were extracted with RIPA buffer in the presence of a protease/phosphatase inhibitor cocktail (Thermo Fisher Scientific). Blots were performed as described previously (61) using different primary antibodies as described in Supplemental Methods. Serotonin levels in cell culture media and tumor tissues were quantified by ELISA (Enzo Life Sciences). 17β-Estradiol levels in cell culture media were quantified by ELISA (RayBiotech). The TPH1 enzymatic activity assay was performed by a TPH1 Inhibitor Screening Assay Kit (Bioscience) following the manufacturer’s instructions.

### Generation of stable OE and KD cells.

Stable shRNA-mediated TPH1 KD was achieved by infecting cells with lentivirus expressing *TPH1* shRNA TRCN0000056626 (shTPH1#1, mainly used in this study and usually dubbed “shTPH1”) or TRCN0000417439 (shTPH1#2), followed by 2-week selection with 2 μg/mL puromycin to establish stable cell lines. A nontarget shCon was used as a control for stable KD cells. Lentivirus production was performed for stably overexpressing TPH1 or Dox-inducible *TPH1* shRNA in PC cells. Briefly, HEK293T cells were cotransfected with a *TPH1*- or Dox-inducible *TPH1* shRNA–expressing lentiviral construct, pCMV Δ R8.2 (Addgene), and pCMV-VSV-G (Addgene) in a 4:2:1 ratio using Lipofectamine 3000 reagent (Thermo Fisher Scientific) following the manufacturer’s instructions. Medium was changed 6 hours after transfection. Medium containing lentivirus was harvested 48 hours after transfection. PC cells were infected with lentivirus in the presence of 8 μg/mL polybrene followed by 2-week selection with 2 μg/mL puromycin. An empty lentiviral construct was used as a control for stable OE of TPH1 in cells.

### Analyses of cell proliferation, colony formation, invasion, sphere formation, and differentiation.

For cell proliferation assays, cells were seeded at 2,000 cells/well in 96-well plates and cultured under the indicated treatments for up to 14 days. Viability was assessed using the CellTiter-Glo assay (Promega) according to the manufacturer’s protocol. For manual counting, cells were plated at 1 × 10^4^ cells/well in 6-well plates, treated under different conditions, trypsinized, and counted with a hemocytometer. Anchorage-independent colony formation was assessed by suspending 200–400 cells/well in 0.3% agarose and plating them over a 0.6% agarose base layer in 6-well plates. Colonies were cultured under the indicated conditions until visible, then stained with nitrotetrazolium blue chloride (Abcam). For invasion assays, 6.5 mm Transwell inserts (8 μm pore size, Corning) coated with growth factor–reduced Matrigel were used. Cells were serum-starved overnight, seeded at 5 × 10^4^ cells/insert in serum-free medium, and cultured under the indicated treatments. After 24–48 hours, cells that migrated to the lower surface were fixed with 4% formaldehyde and stained with 1% crystal violet. Images were acquired on a Leica microscope, and invading cells were quantified by counting stained nuclei in 3 random fields per insert using ImageJ (NIH). Invasion of LASCPC-01 cells was assessed using Falcon FluoroBlok inserts, and invading cells were visualized for fluorescence after 48 hours with a ZEISS Axio Imager M2 microscope. For 3D invasion assays, 5 × 10^4^ cells in 10 μL of 100% Matrigel were seeded per well in 6-well plates, with medium replenished every 3 days. Gel drops were imaged on days 0 and 8 using a Leica inverted microscope, and invasive outgrowth was quantified by ImageJ (NIH). For tumorsphere formation assays, 1,000 cells/well were seeded in ultra-low-attachment, 24-well plates (Corning) in serum-free PrEBM medium (Lonza) supplemented with B-27 (Thermo Fisher Scientific), 20 ng/mL EGF (Thermo Fisher Scientific), 10 ng/mL bFGF (Thermo Fisher Scientific), 5 μg/mL insulin, and 0.4% BSA (Sigma-Aldrich). Medium was refreshed every 3 days, and spheres were allowed to form for 1–2 weeks. Colonies and spheres were imaged with a ChemiDoc Touch Imager (Bio-Rad Laboratories), and those containing at least 50 cells were quantified using ImageJ. For the assessment of cell differentiation, cells were seeded at 2 × 10^4^ cells/well in 6-well plates and subjected to different treatments. Morphological differentiation was evaluated from phase-contrast images acquired on days 1 and 5 after plating. Neurite number was quantified, and neurite length was manually measured using the tracing tool in ImageJ.

### Analyses of PDX-derived organoid growth.

The LuCaP 49, 93, and 173.1 NEPC PDX models were obtained from the University of Washington. The PDX tumor tissue expanded in male 4- to 6-week-old NSG mice (The Jackson Laboratory) was harvested for single-cell suspension preparation as described previously (62). Cells were suspended in complete organoid culture medium supplemented with *TPH1* shRNA–expressing lentivirus and 8 μg/mL polybrene, incubated overnight at 37°C, and embedded in growth factor–reduced Matrigel in 48-well plates for viability assays and imaging or in 12-well plates for RNA extraction. Puromycin at 2 μg/mL was added 24 hours postseeding and maintained for 3 days to select for transduced cells. The culture medium was replenished every 2–3 days. The viability of organoids was measured using a Calcein AM dye–based LIVE/DEAD Viability/Cytotoxicity Kit (Thermo Fisher Scientific) following the manufacturer’s instructions, and images were acquired on a ZEISS Axio Imager M2 microscope and analyzed with HALO software. The viability of organoids was also measured using a CellTiter-Glo 3D cell viability assay kit (Promega) according to the manufacturer’s instructions.

### Animal studies.

Male, 5-week-old NGS mice were purchased from The Jackson Laboratory, housed in the animal research facility at Washington State University (WSU), and fed a normal chow diet. For determining the effect of TPH1 on NEPC tumor development and growth, 4 × 10^6^ control and TPH1-KD PC cells were mixed 1:1 with Matrigel for bilateral subcutaneous injection into NSG mice. Tumor sizes were measured every 2–3 days by caliper after the formation of palpable tumors, and tumor volumes were calculated as 1/2 × length × width^2^. At the endpoints, tumors were collected, weighed, and processed for subsequent analyses. For determining the effect of LX1606 alone or in combination with Enz on NEPC tumor growth, 4 × 10^6^ PC cells were mixed 1:1 with Matrigel for bilateral subcutaneous injection into NSG mice. For PDX studies, the LuCaP tumor tissue was cut into ~5 to 10 mm^3^ pieces and implanted s.c. into both flanks of the mice. When tumors reached ~100 mm^3^, mice were randomly divided into groups (6–10 tumors per group) to receive vehicle (saline, p.o., daily), LX1606 (200 mg/kg, p.o., daily), Enz (10 mg/kg, p.o., daily), or combination treatment. Tumor volumes were measured every 2–3 days and calculated as described above. At the endpoints, tumors were collected, weighed, and processed for subsequent analyses.

### TGM2 monoaminylation assays.

Monoaminylation assays were performed by incubating 3 μg of human recombinant mTOR (aa 2,226–2,488) protein (LSBio) with 5 mM MDC and 0.25 μg of human recombinant TGM2 protein (Novus Biologicals) in monoaminylation buffer A (25 mM Tris-HCl, pH 8.0; 5 mM CaCl_2_; and protease inhibitors). The mixture was incubated at 37°C overnight in a total volume of 30 μL. The reaction was also treated with 4 mM TGM2 inhibitor cystamine or competed with excess 500 μM serotonin. The human recombinant fibrinogen protein (Sigma-Aldrich) served as a positive control in a parallel reaction. Following incubation, the reaction was quenched with 4× SDS loading buffer and analyzed by immunoblotting. The MDC-transamidated proteins were visualized under UV light, followed by Coomassie blue staining for protein loading.

### LC-MS/MS identification of monoaminylation and serotonylation on mTOR.

Transamidation of MDC or serotonin to 3 μg of human recombinant mTOR protein or 10 μg of mTOR peptide (aa 2,446–2,460) synthesized by GenScript was performed using the same procedures described above. Protein samples were reduced with 100 mM dithiothreitol (DTT) in 4% SDS buffer at 95°C for 10 minutes, alkylated with 200 mM iodoacetamide in the dark at room temperature for 30 minutes, and precipitated with cold acetone at –20°C overnight. Precipitated proteins were collected by centrifugation at 16,000*g* for 20 minutes at 4°C, washed with cold methanol, and centrifuged again under the same conditions. Protein pellets were resuspended in 50 mM ammonium bicarbonate buffer and digested with sequencing-grade trypsin at an enzyme-to-protein ratio of 1:30 at 37°C overnight. Digestion was quenched by adjusting the pH to 3–4 with formic acid, and peptides were subjected to LC-MS/MS analysis. Tryptic peptides were analyzed on an Easy-nLC 1200 system (Thermo Fisher Scientific) coupled to a Q Exactive Orbitrap mass spectrometer (Thermo Fisher Scientific). Separation was performed on a PepMap RSLC C18 column (25 cm × 50 μm, 2 μm, 100 Å) at 300 nL/min using a 70-minute linear gradient: 0–2 min, 0%–10% buffer B; 2–27 min, 10%–45% buffer B; 27–28 min, 45%–100% buffer B; and 45–70 min, 100% buffer B, where buffer A was 0.1% formic acid in water and buffer B was 80% acetonitrile in 0.1% formic acid. The mass spectrometer was operated in parallel reaction monitoring mode with a spray voltage of 1.7 kV and a capillary temperature of 300°C. MS1 spectra were acquired over an *m/z* range of 400–1,200 at a resolution of 120,000 with an AGC target of 3 × 10^6^ and maximum injection time of 200 ms, and MS2 spectra were acquired at a resolution of 30,000 with an AGC target of 2 × 10^5^, normalized collision energy of 27. Both unmodified and MDC/serotonin-modified peptides were included in the acquisition method. Raw data were processed using MaxQuant (v2.7.0.0) and Skyline-daily for peptide identification and confirmation.

### IHC and IF analyses.

IHC analysis of clinical specimens and xenograft tumors, multiplex IF analysis of clinical specimens, and IF analysis of cultured cells were performed using antibodies against TPH1 (Sigma-Aldrich, HPA022483), SYP (Santa Cruz Biotechnology, sc-17750), CD56 (Cell Signaling Technology, 99746), Ki-67 (Cell Signaling Technology, 9027), serotonin (Novus Biologicals, NB120-16007), AR (Cell Signaling Technology, 5153), or PSA (Santa Cruz Biotechnology, sc-7638) following our published protocol (63). Serial tissue sections were used for IHC staining of TPH1 and SYP separately as in Figure 1B. The IHC and IF data were quantitatively analyzed by assigning H-score, cell-based intensity, or percentage of Ki-67^+^ expression for individual staining by HALO software after areas of interest were defined using manual tissue segmentation.

### RNA-seq and bioinformatic analyses.

The total RNA of control and TPH1-KD C4-2B^ENZR^ and LASCPC-01 cells and control and TPH1-OE LNCaP cells was extracted by a RNeasy Mini Kit and underwent DNase digestion following the manufacturer’s instructions. RNA-seq was performed on an Illumina HiSeq 4000 at Novogene. Bowtie 2 v2.1.0 was used for mapping to the human genome hg19 transcript set. RSEM v1.2.15 was used to calculate the count and estimate the gene expression levels. Trimmed mean of M-values method in the edge R package was used for gene expression normalization. GSEA v4.3.3 was used to evaluate the enrichment of different gene sets from the Molecular Signatures Database (MSigDB v2023.2 Hs) or curated based on related studies (62). The IPA (QIAGEN) was used to identify the upstream regulators for the transcriptomic changes in RNA-seq data. The human PC (Beltran 2016 [ref. 15], GSE74685, GSE32967, GSE126078, GSE90891, and GSE197780) datasets of gene expression used for different correlation studies or examination of *TPH1* expression between different disease states were downloaded from cBioPortal and the National Center for Biotechnology Information (NCBI) Gene Expression Omnibus (GEO).

### Statistics.

Data are presented as mean ± SEM as indicated in the figure legends. Correlations between groups were determined by Pearson’s correlation. All other comparisons were analyzed by paired and unpaired 2-tailed Student’s *t* test, 1-way ANOVA with Tukey’s or Dunnett’s multiple-comparison test, or 2-way ANOVA with Holm-Šídák multiple-comparison test. A *P* value less than 0.05 was considered statistically significant.

### Study approval.

All animal studies received prior approval from the Institutional Animal Care and Use Committee (IACUC) at WSU and complied with IACUC recommendations. Human tissue specimens were obtained from the University of Washington site of the Prostate Cancer Biorepository Network and the Biobank of Taipei Veterans General Hospital under approved protocols from their local IRBs and with written informed consent received from patients.

### Data availability.

The RNA-seq data generated in this study are available in the NCBI GEO with accession number GSE302430. Detailed data values for all graphs and values behind any reported means of the study are available in a separate Supporting Data Values XLS file.

## Manuscript information

### Author contributions.

J Wei and BJW conceived the study and wrote the manuscript. J Wei, J Wang, JC, MZ, CHC, TP, AO, and SH performed experiments and data analysis. EC, TPL, and ACG provided material support. BJW provided project administration, supervised the study, and acquired funding.

### AI disclosure.

The authors declare that no AI tools were used in the original research or writing of this article.

### Conflict of interest.

EC served as a paid consultant to DotQuant and received institutional sponsored research funding from AstraZeneca, AbbVie, Gilead, Sanofi, Zenith Epigenetics, Bayer Pharmaceuticals, Forma Therapeutics, Genentech, GlaxoSmithKline, Janssen Research, Kronos Bio, Foghorn Therapeutics, K36 Therapeutics, and MacroGenics.

### Funding.

This study was supported by the following:

- NIH grants R01CA279528, R37CA233658, and R01CA258634 to BJW.
- Department of Defense Prostate Cancer Research Program grant W81XWH-21-1-0218 to J Wei.
- Department of Defense Prostate Cancer Research Program grants W81XWH-18-2-0013, W81XWH-18-2-0015, W81XWH-18-2-0016, W81XWH-18-2-0017, W81XWH-18-2-0018, and W81XWH-18-2-0019 to the Prostate Cancer Biorepository Network.
- The Pacific Northwest Cancer SPORE P50CA097186, the Department of Defense Prostate Cancer Biorepository Network W81XWH-14-2-0183, and NIH grant P01CA163227 for the establishment and characterization of LuCaP PDX models.

This work is the result of NIH funding, in whole or in part, and is subject to the NIH Public Access Policy. Through acceptance of this federal funding, the NIH has been given a right to make the work publicly available in PubMed Central.

### Present addresses.

J. Wang’s present address is: Department of Medical Oncology, Dana-Farber Cancer Institute, Boston, Massachusetts, USA. TP’s present address is: Integrated Genomics Operation, Memorial Sloan Kettering Cancer Center, New York, New York, USA.

## Supplementary Material

Supplemental data

Unedited blot and gel images

Supporting data values

## Figures and Tables

**Figure 1:**
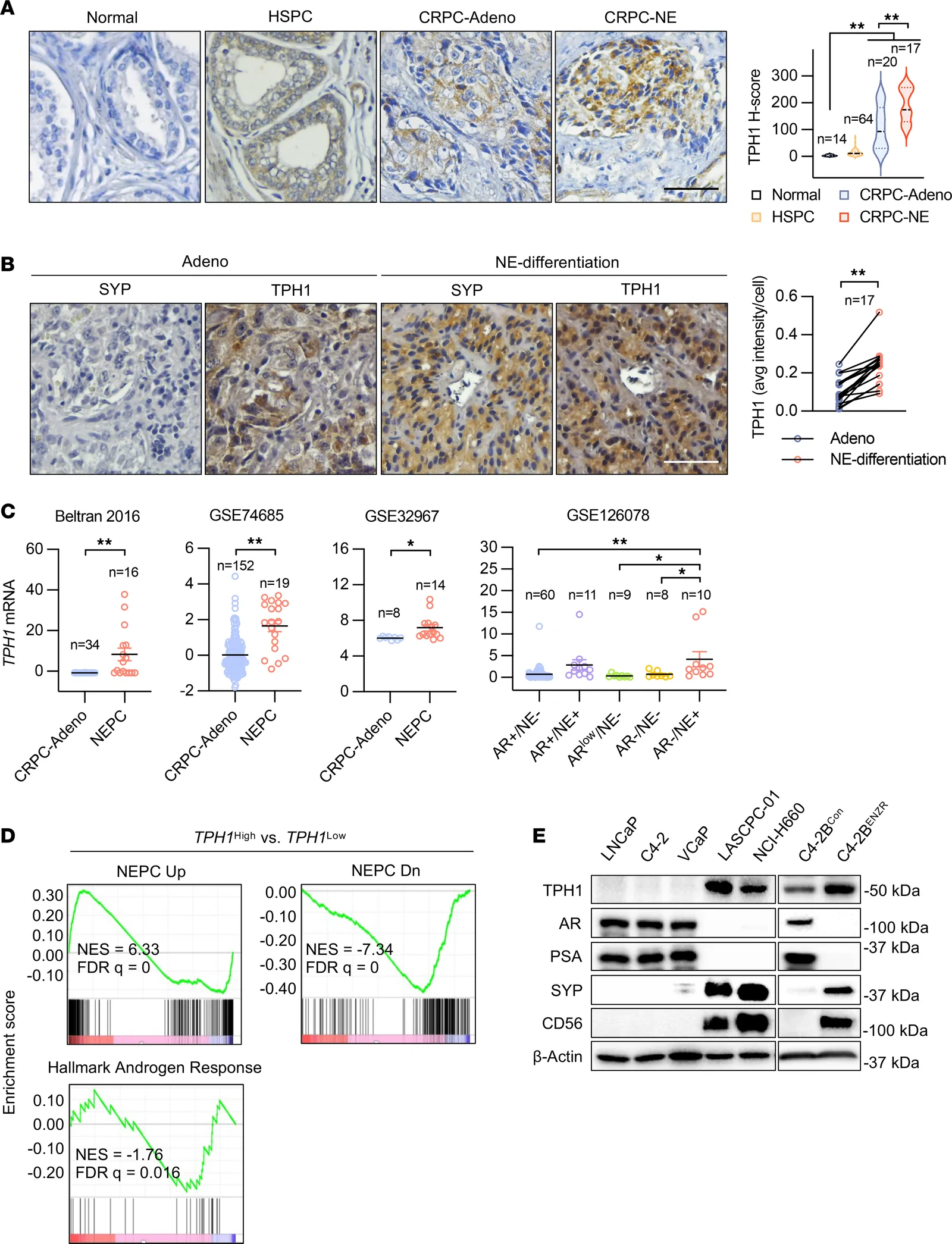
TPH1 is highly expressed in human NEPC. (**A**) Representative images and quantification of TPH1 IHC staining in normal prostates (*n* = 14), HSPC (*n* = 64), CRPC-Adeno (*n* = 20), and CRPC-NE (*n* = 17). Scale bar: 50 μm. (**B**) Representative TPH1 IHC staining and quantification in SYP^+^ NE-differentiated versus SYP-Adeno areas of the same CRPC-NE samples (*n* = 17) in **A**. Scale bar: 50 μm. (**C**) *TPH1* mRNA in NEPC versus CRPC-Adeno (Beltran 2016 [ref. 15], GSE74685, and GSE32967), as well as in a PC cohort (GSE126078) categorized by AR and NE status. (**D**) Gene set enrichment analysis (GSEA) of NEPC and androgen response gene sets enriched in *TPH1*-high versus -low CRPC patient samples from the Beltran 2016 dataset. (**E**) Western blot of TPH1, AR, PSA, SYP, and CD56 protein expression in a panel of human PC cell lines. Data represent mean ± SEM. Statistics were calculated using 1-way ANOVA with Tukey’s test (**A** and **C**, right panel), paired 2-tailed Student’s *t* test (**B**), unpaired 2-tailed Student’s *t* test (**C**, left 3 panels), or permutation test (**D**). *P < 0.05, **P < 0.01.

**Figure 2:**
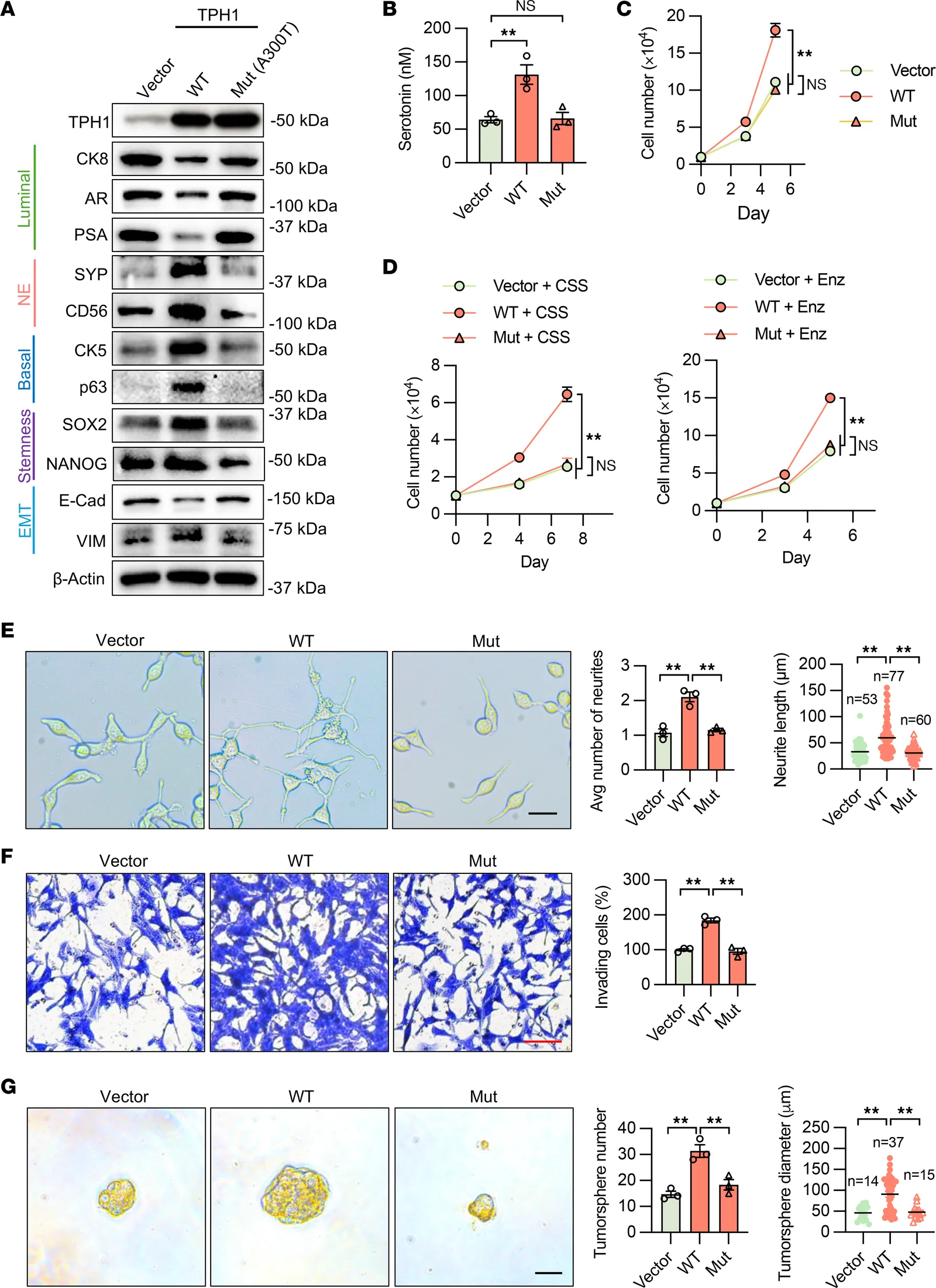
TPH1 confers a NE phenotype in AdPC cells. (**A**) Western blot of TPH1, luminal, NE, basal, stemness, and epithelial-mesenchymal transition (EMT) markers in control, WT, and mutant TPH1-OE LNCaP cells. (**B**) ELISA of serotonin levels in the culture media from control, WT, and mutant TPH1-OE LNCaP cells (*n* = 3). (**C**) Proliferation of control, WT, and mutant TPH1-OE LNCaP cells (*n* = 3). (**D**) Proliferation of control, WT, and mutant TPH1-OE LNCaP cells with 5% charcoal-stripped serum (CSS) or 10 μM Enz (*n* = 3). (**E**) Representative images of control, WT, and mutant TPH1-OE LNCaP cell morphology and quantification of per-cell neurite number and length in each group (*n* = 3, with >50 cells per group), representing 3 independent experiments. Scale bar: 20 μm. (**F**) Representative images and quantification of cell invasion by control, WT, and mutant TPH1-OE LNCaP cells (*n* = 3). Scale bar: 50 μm. (**G**) Representative images and quantification of the number and size of tumorspheres formed by control, WT, and mutant TPH1-OE LNCaP cells (*n* = 3). Scale bar: 50 μm. Data represent mean ± SEM. Statistics were calculated using 1-way ANOVA with Tukey’s test (**B**–**G**). **P < 0.01.

**Figure 3:**
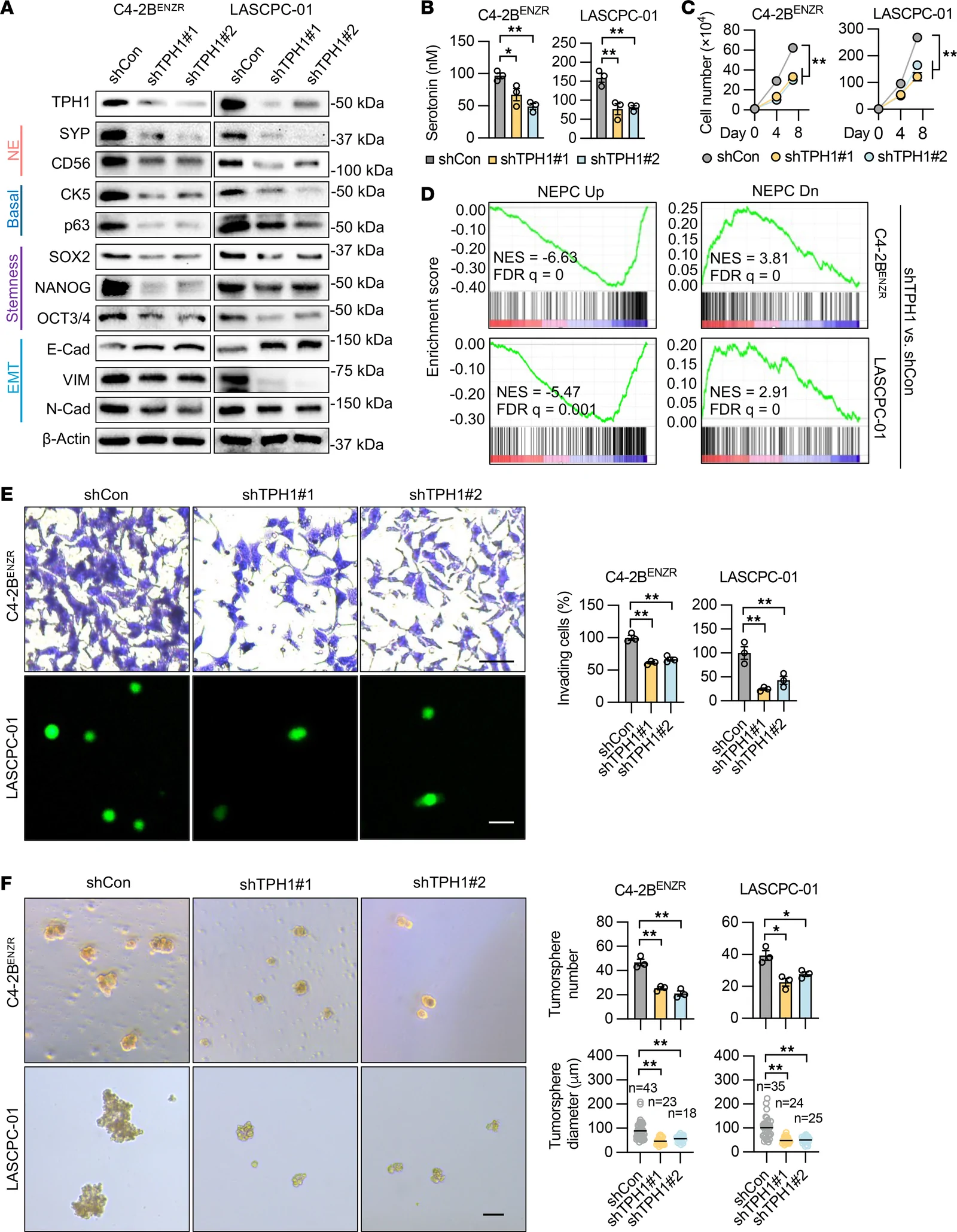
TPH1 silencing impairs NE plasticity and aggressive behavior in NEPC cells. (**A**) Western blot of TPH1, NE, basal, stemness, and EMT markers in control and TPH1-KD NEPC cells. (**B**) ELISA of serotonin levels in the culture media of control and TPH1-KD NEPC cells (*n* = 3). (**C**) Proliferation of control and TPH1-KD NEPC cells (*n* = 3). (**D**) GSEA of NEPC gene signatures enriched in TPH1-KD versus control NEPC cells. (**E**) Representative images and quantification of cell invasion by control and TPH1-KD NEPC cells (*n* = 3). Scale bars: 50 μm. (**F**) Representative images and quantification of the number and size of tumorspheres formed by control and TPH1-KD NEPC cells (*n* = 3). Scale bar: 50 μm. Data represent mean ± SEM. Statistics were calculated using 1-way ANOVA with Dunnett’s test (**B**, **C**, **E**, and **F**) or permutation test (**D**). *P < 0.05, **P < 0.01.

**Figure 4:**
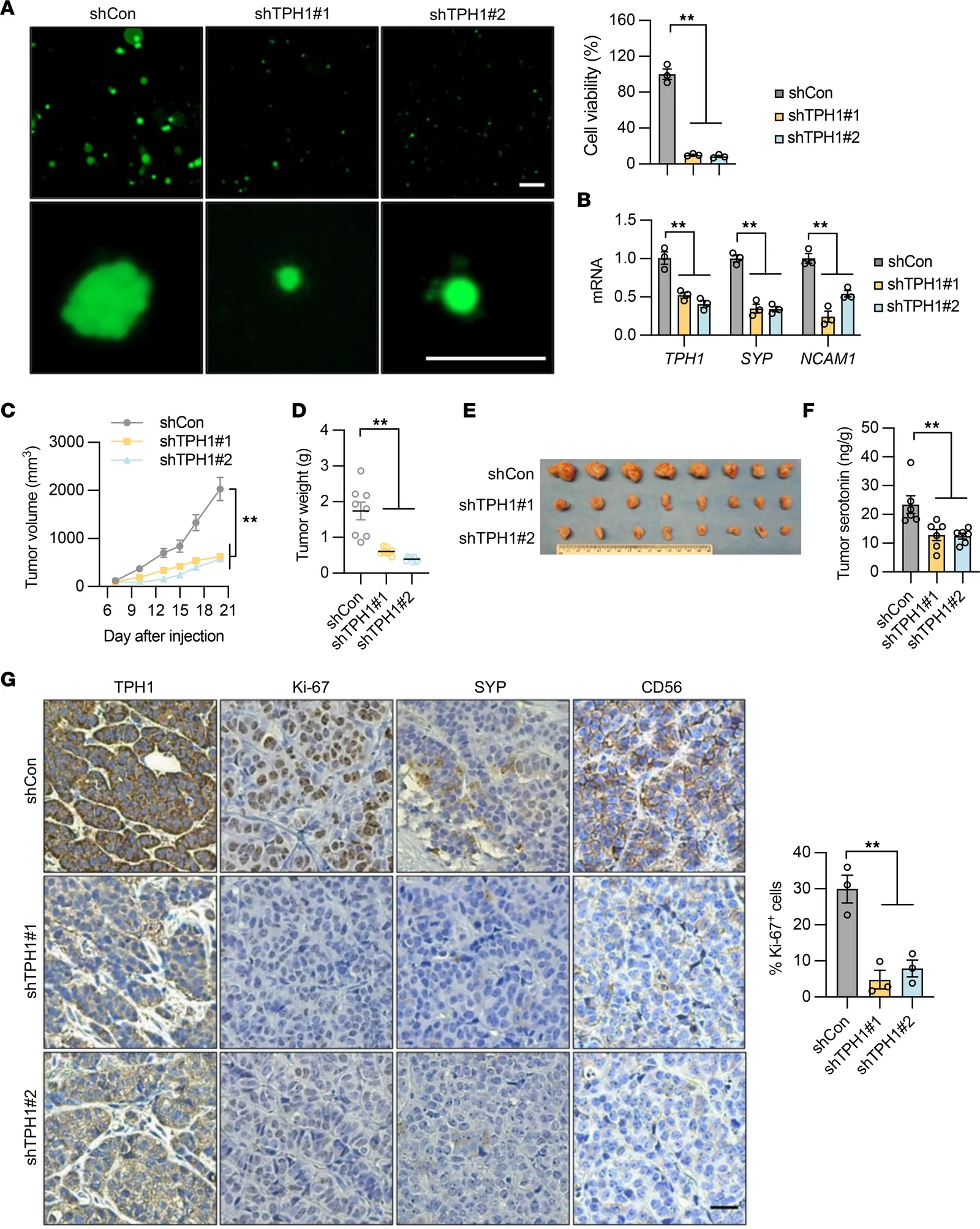
TPH1 depletion suppresses NEPC organoid and tumor growth. (**A**) Representative fluorescence microscopic images and viability of control and TPH1-KD LuCaP 49 PDX-derived organoids (*n* = 3). Scale bars: 100 μm. (**B**) qPCR of *TPH1*, *SYP*, and *NCAM1* in control and TPH1-KD organoids as in **A** (*n* = 3). (**C**–**E**) Tumor growth curves (**C**), endpoint tumor weights (**D**), and anatomical tumor image (**E**) of control (shCon) and TPH1-KD (shTPH1) LASCPC-01 xenografts grown s.c. in male NSG mice (*n* = 8). (**F**) ELISA of serotonin levels in control and TPH1-KD LASCPC-01 tumors (*n* = 6). (**G**) Representative IHC staining of TPH1, Ki-67, SYP, and CD56 and quantification of percentage of Ki-67^+^ cells (*n* = 3) in control and TPH1-KD LASCPC-01 tumors. Scale bar: 20 μm. Data represent mean ± SEM. Statistics were calculated using 1-way ANOVA with Dunnett’s test (**A**–**D**, **F**, and **G**). **P < 0.01.

**Figure 5:**
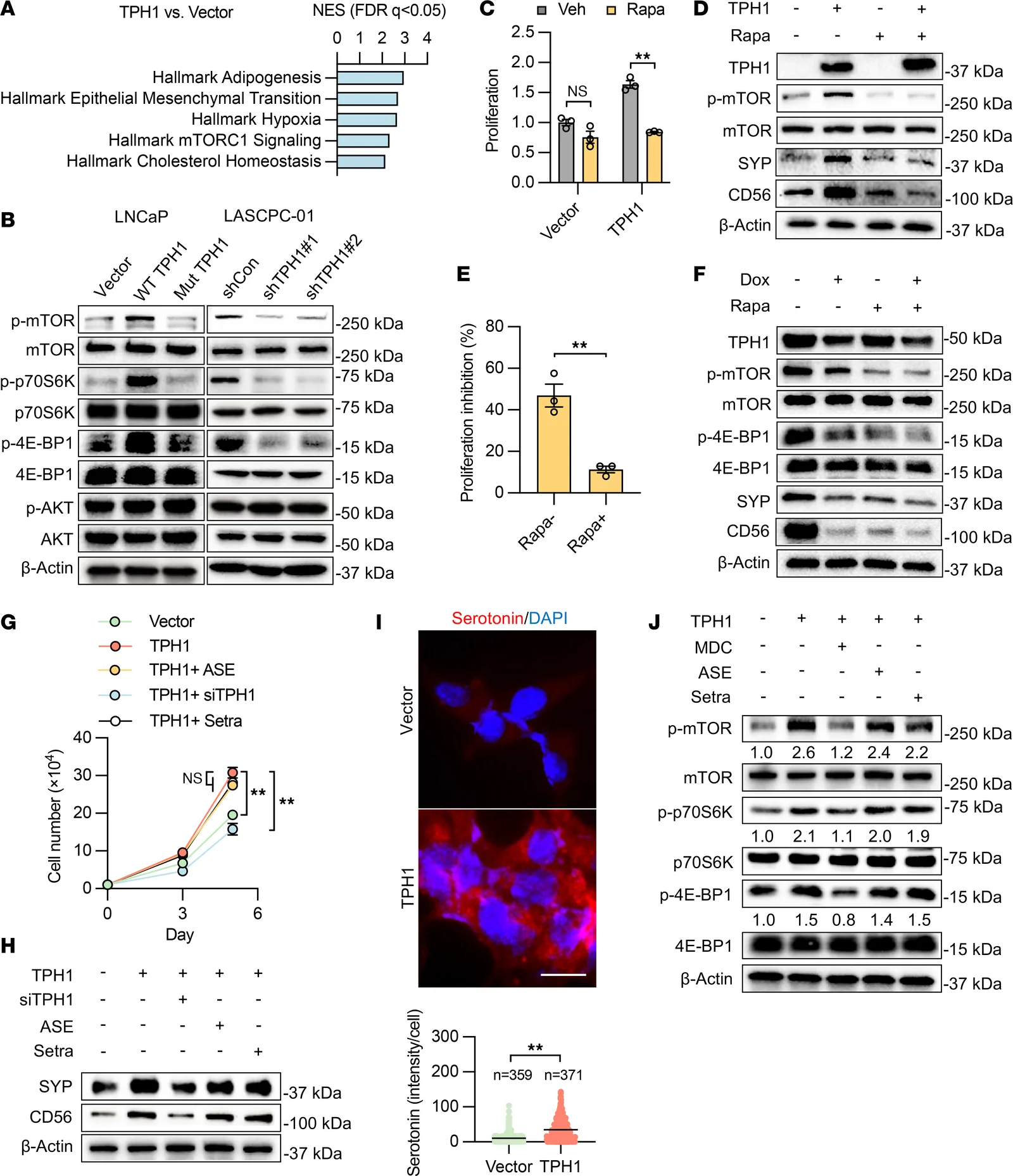
TPH1 activates mTOR through intracellular serotonin in NEPC cells. (**A**) GSEA of the top 5 Hallmark gene sets positively enriched in TPH1-OE LNCaP cells. (**B**) Western blot of mTOR and related phosphoproteins in control and TPH1-manipulated PC cells. p-, phosphorylated. (**C**) Proliferation of control and TPH1-OE LNCaP cells with rapamycin (25 nM, 3 days) (*n* = 3). (**D**) Western blot of TPH1, p-mTOR, and NE markers in control and TPH1-OE LNCaP with rapamycin for 48 hours. (**E**) Proliferation of Dox-inducible TPH1-KD LASCPC-01 cells pretreated with rapamycin for 24 hours followed by Dox stimulation (100 ng/mL, 3 days) (*n* = 3), with the percentage of proliferation inhibition shown. (**F**) Western blot of TPH1, p-mTOR, p-4E-BP1, and NE markers in LASCPC-01 cells as in **E**. (**G**) Proliferation of control and TPH1-OE LNCaP cells with 10 μM asenapine (ASE), 2 μM sertraline (Setra), or *TPH1* siRNA (*n* = 3). (**H**) Western blot of NE markers in control and TPH1-OE LNCaP cells under the same treatments as in **G** for 48 hours. (**I**) Representative IF staining and quantification of per-cell perinuclear intracellular serotonin in control and TPH1-OE LNCaP cells, with more than 350 cells per group. Scale bar: 20 μm. (**J**) Western blot of mTOR and downstream phosphoproteins in control and TPH1-OE LNCaP cells with ASE, Setra, or 200 μM MDC for 30 minutes. Data represent mean ± SEM. Statistics were calculated using 2-way ANOVA with Holm-Šídák test (**C**), unpaired 2-tailed Student’s *t* test (**E** and **I**), or 1-way ANOVA with Tukey’s test (**G**). **P < 0.01.

**Figure 6:**
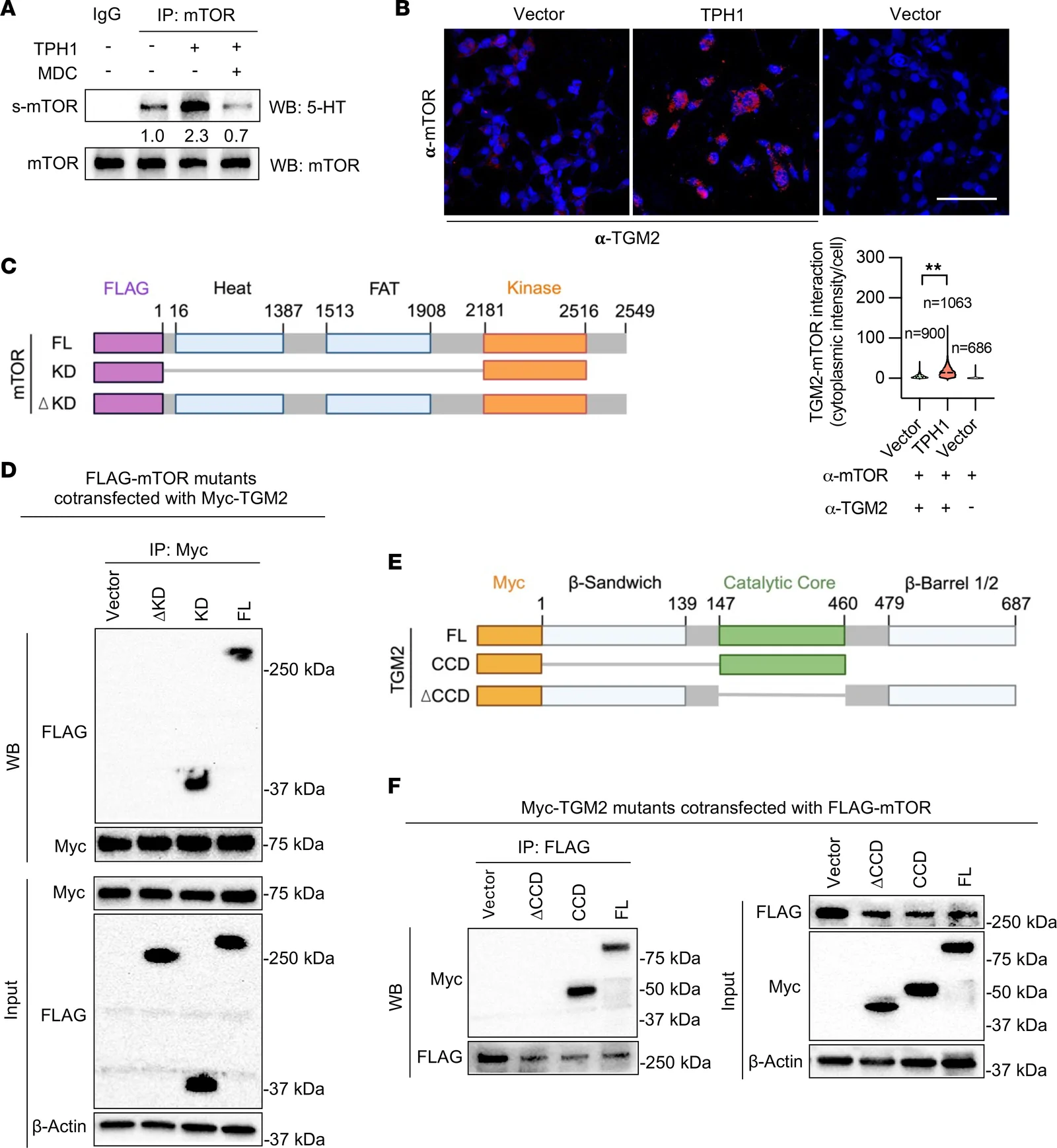
TPH1 enhances mTOR serotonylation and mTOR-TGM2 interaction. (**A**) Co-IP assay of mTOR-serotonin interaction in control and TPH1-OE LNCaP cells with MDC (200 μM, 24 hrs), with normalized serotonylated mTOR (s-mTOR) levels in untreated control cells set as 1. (**B**) Representative proximity ligation assay staining and quantification of mTOR-TGM2 interaction by per-cell cytoplasmic fluorescence intensity in control and TPH1-OE LNCaP cells. Scale bar: 50 μm. (**C** and **D**) Co-IP assay of FL or truncated FLAG-mTOR constructs (**C**) cotransfected with Myc-TGM2 construct in HEK293T cells (**D**). (**E** and **F**) Co-IP assay of FL or truncated Myc-TGM2 (**E**) cotransfected with FLAG-mTOR in HEK293T cells (**F**). Data represent mean ± SEM. Statistics were calculated using 1-way ANOVA with Dunnett’s test (**B**). **P < 0.01.

**Figure 7:**
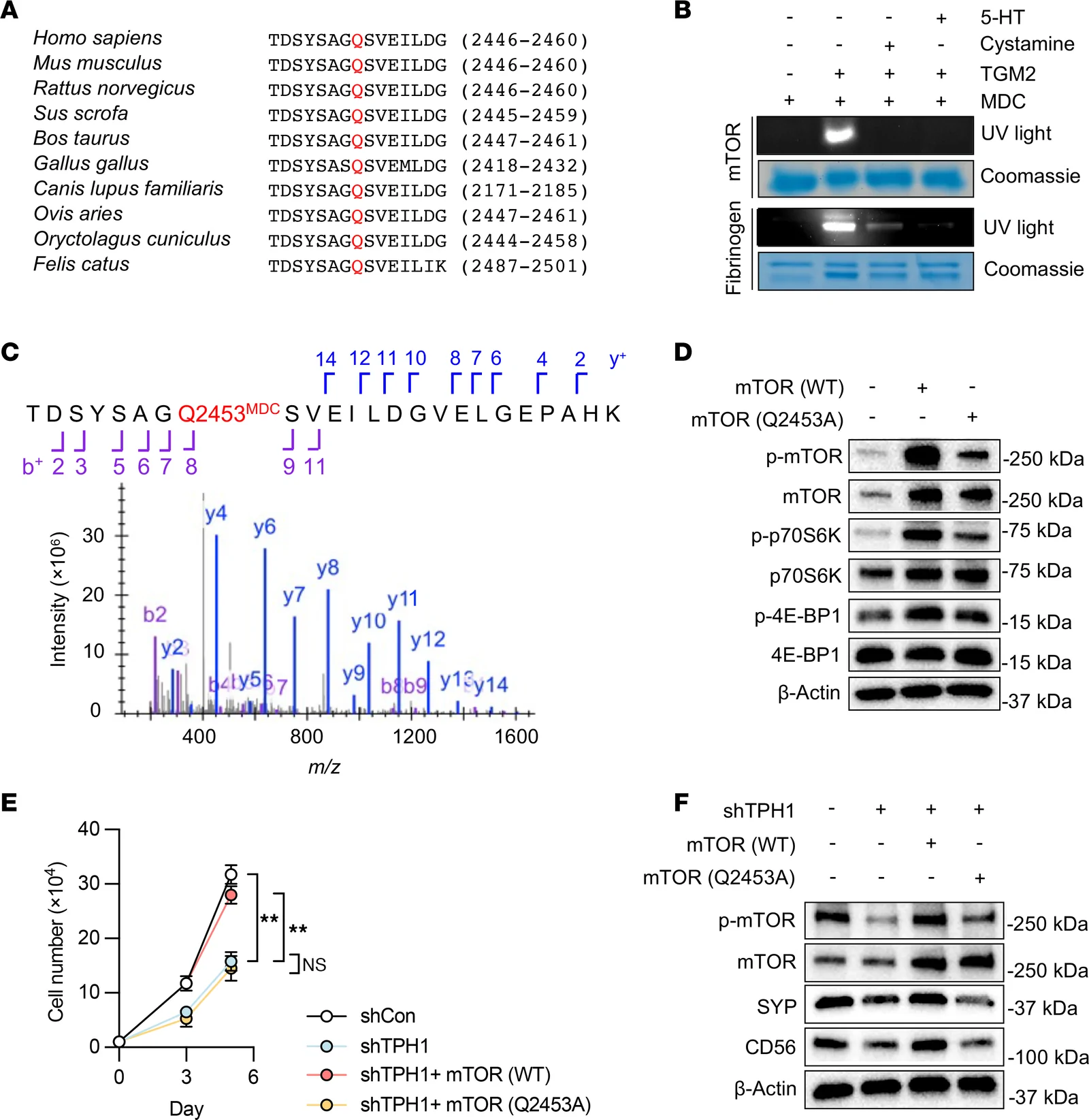
Serotonylation of Gln2453 promotes mTOR activity and TPH1 function in NEPC cells. (**A**) Conserved Gln2453 and flanking sequences on mTOR across different species. (**B**) TGM2 monoaminylation assays using human recombinant mTOR (aa 2,226–2,488) protein or fibrinogen protein as a control, under 4 mM cystamine or donor competition with excess 500 μM serotonin, shown as a representative of 3 independent experiments. (**C**) LC-MS/MS analysis of TGM2-transamidated MDC to recombinant mTOR protein (aa 2,226–2,488). (**D**) Western blot of mTOR and downstream phosphoproteins in LASCPC-01 cells transfected with WT or mutant (Q2453A) mTOR. (**E**) Proliferation of control and TPH1-KD LASCPC-01 cells expressing WT or mutant mTOR (*n* = 3). (**F**) Western blot of p-mTOR and NE markers in control and TPH1-KD LASCPC-01 cells expressing WT or mutant mTOR. Data represent mean ± SEM. Statistics were calculated using 1-way ANOVA with Tukey’s test (**E**). **P < 0.01.

**Figure 8:**
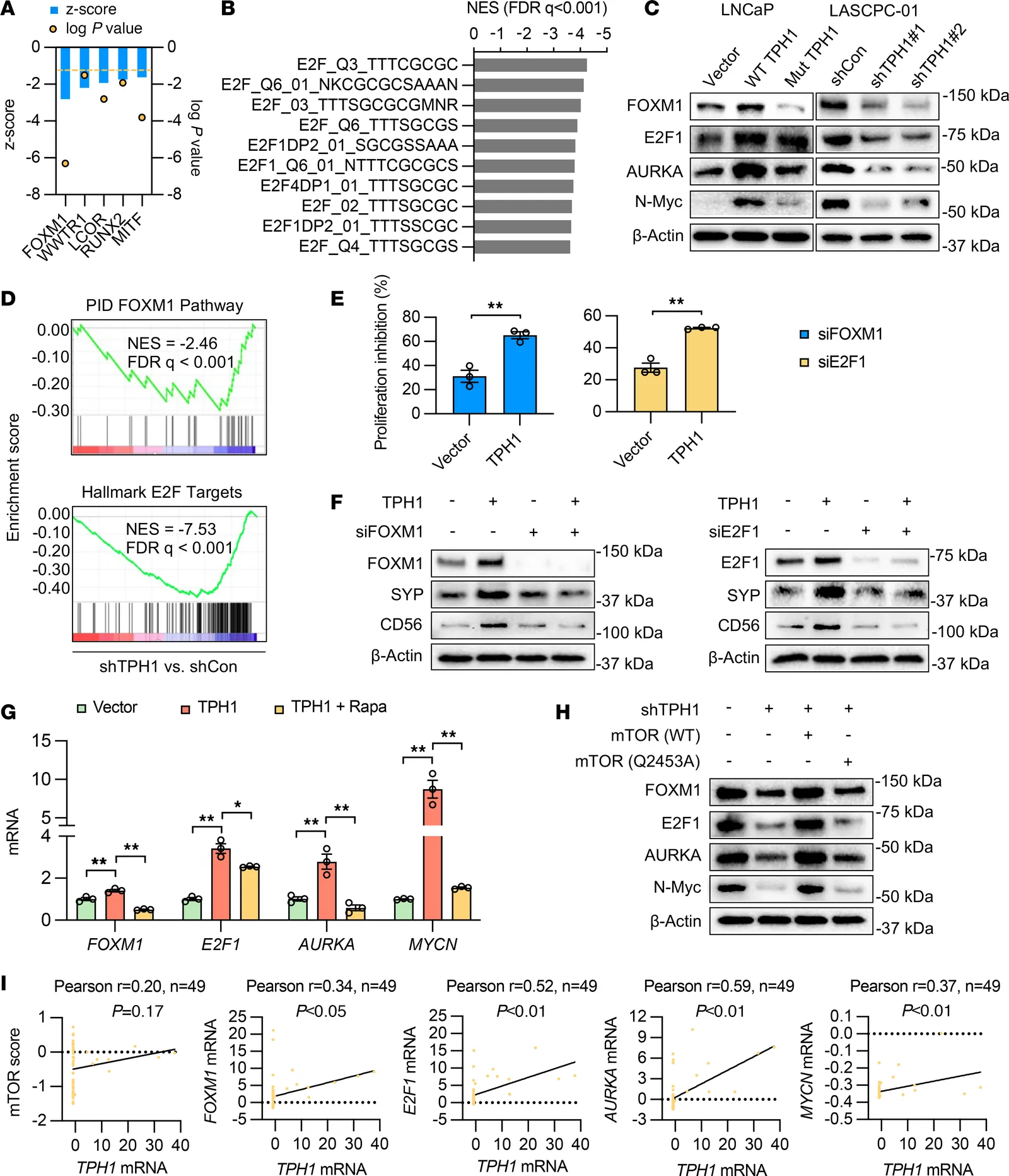
TPH1 induces FOXM1 and E2F1 via mTOR to function in NEPC cells. (**A**) Ingenuity Pathway Analysis (IPA; QIAGEN) upstream regulator analysis of RNA-seq data from TPH1-KD versus control C4-2B^ENZR^ cells, with significantly downregulated hits shown (*P* < 0.05). (**B**) GSEA of the top 10 enriched E2F-binding motifs in regions spanning 4 kb centered on the transcription start sites of genes differentially expressed in TPH1-KD versus control C4-2B^ENZR^ cells. (**C**) Western blot of FOXM1, E2F1, AURKA, and N-Myc in control and TPH1-manipulated PC cells. (**D**) GSEA of FOXM1 and E2F-directed gene sets enriched in TPH1-KD versus control C4-2B^ENZR^ cells. PID, Pathway Interaction Database. (**E**) Proliferation of control and TPH1-OE LNCaP cells with *FOXM1* or *E2F1* siRNAs for 3 days (*n* = 3), with percentage proliferation inhibition shown. (**F**) Western blot of FOXM1, E2F1, and NE markers in control and TPH1-OE LNCaP cells with *FOXM1* or *E2F1* siRNAs for 48 hours. (**G**) qPCR of *FOXM1*, *E2F1*, *AURKA*, and *MYCN* in control and TPH1-OE LNCaP cells with rapamycin (25 nM, 24 hrs) (*n* = 3). (**H**) Western blot of FOXM1, E2F1, AURKA, and N-Myc in control and TPH1-KD C4-2B^ENZR^ cells expressing WT or mutant (Q2453A) mTOR. (**I**) Pearson’s correlation analysis of *TPH1* mRNA with mTOR score and *FOXM1*, *E2F1*, *AURKA*, and *MYCN* mRNA in the Beltran 2016 dataset. Data represent mean ± SEM. Statistics were calculated using permutation test (**D**), unpaired 2-tailed Student’s *t* test (**E**), 1-way ANOVA with Tukey’s test (**G**), or Pearson’s correlation coefficient *t* test (**I**). *P < 0.05, **P < 0.01.

**Figure 9:**
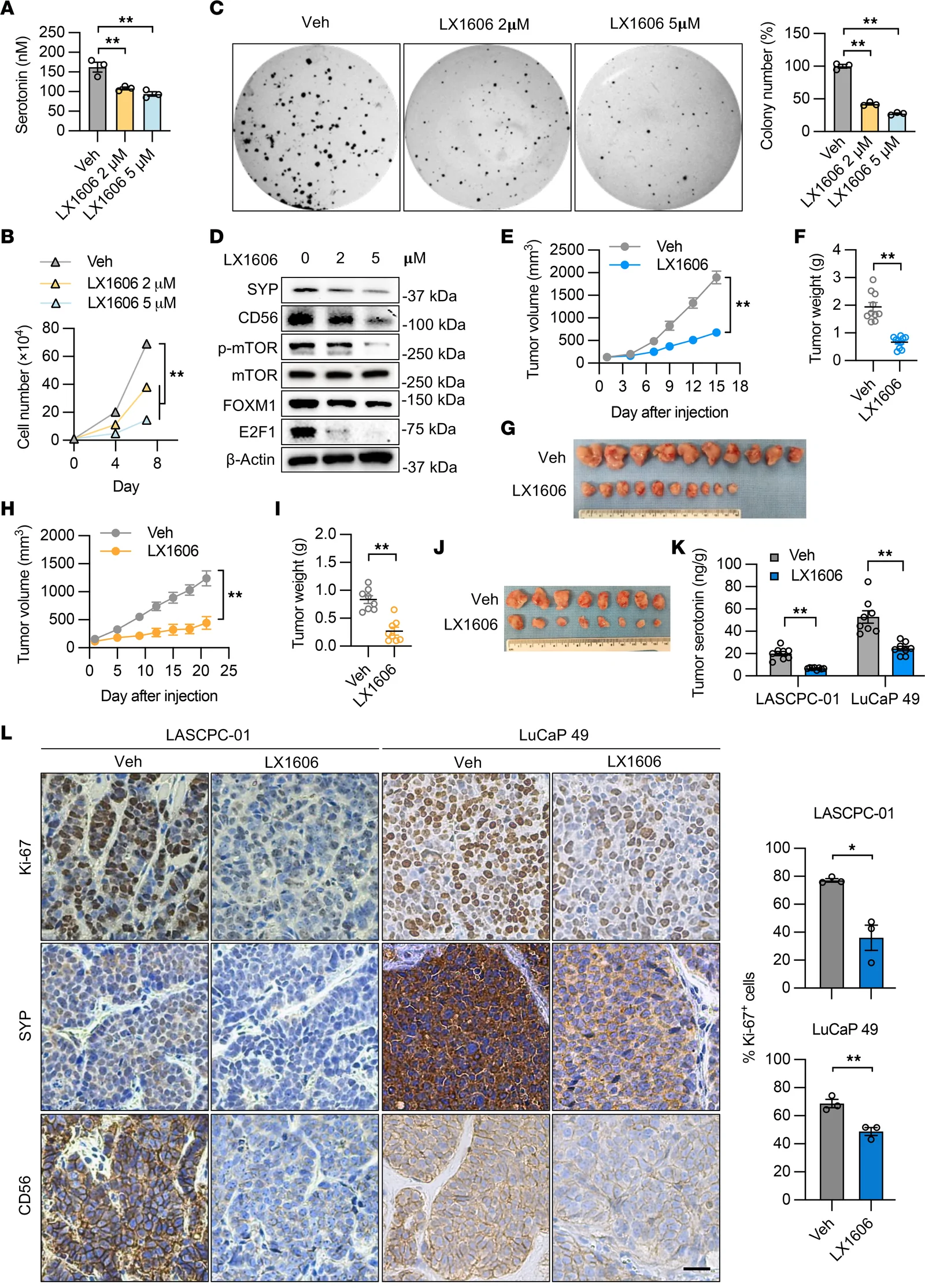
Pharmacological TPH1 inhibitor LX1606 restricts NEPC differentiation and growth. (**A**) ELISA of serotonin levels in the culture media of LASCPC-01 cells with LX1606 for 48 hours (*n* = 3). (**B**) Proliferation of LASCPC-01 cells with LX1606 (*n* = 3). (**C**) Representative images and quantification of soft-agar colony formation by LASCPC-01 cells with LX1606 (*n* = 3). (**D**) Western blot of SYP, CD56, p-mTOR, FOXM1, and E2F1 in LASCPC-01 cells with LX1606 for 3 days. (**E**–**G**) Tumor growth curves (**E**), endpoint tumor weights (**F**), and anatomic tumor image (**G**) of LASCPC-01 xenografts grown s.c. in male NSG mice receiving vehicle (Veh) or LX1606 (*n* = 10). (**H**–**J**) Tumor growth curves (**H**), endpoint tumor weights (**I**), and anatomic tumor image (**J**) of LuCaP 49 PDXs grown s.c. in male NSG mice receiving vehicle or LX1606 (*n* = 8). (**K**) ELISA of tumor serotonin levels in control and LX1606-treated groups (*n* = 8 or 10). (**L**) Representative IHC staining of Ki-67, SYP, and CD56 and quantification of percentage of Ki-67^+^ cells (*n* = 3) in control and LX1606-treated tumors. Scale bar: 20 μm. Data represent mean ± SEM. Statistics were calculated using 1-way ANOVA with Dunnett’s test (**A**–**C**) or unpaired 2-tailed Student’s *t* test (**E**, **F**, **H**, **I**, **K**, and **L**). *P < 0.05, **P < 0.01.

## Version 1 09/17/2026

In-Press Preview

## Version 2 11/16/2026

Electronic publication
