# Supplementary material for: Targeting tryptophan hydroxylase 1 restricts growth and suppresses plasticity in neuroendocrine prostate cancer
Source: J Clin Invest. 2026 Sep 17;136(22):e200391. doi: 10.1172/JCI200391 (PMC13767635; doi:10.1172/JCI200391)
Supplement: Supplementary file 1 [file jci-136-200391-s010.pdf]

Supplemental Material

Targeting Tryptophan Hydroxylase 1 Restricts Growth and Suppresses Plasticity in Neuroendocrine Prostate Cancer

Jing Wei, Jing Wang, Jingrui Chen, Michelle Zhang, Chia-Hui Chen, Tianjie Pu, Alivia O'Brien, Sephtis Hargrove, Eva Corey, Tzu-Ping Lin, Allen C. Gao, Boyang Jason Wu

Supplemental Figures 1-18
Supplemental Table 1
Supplemental Methods

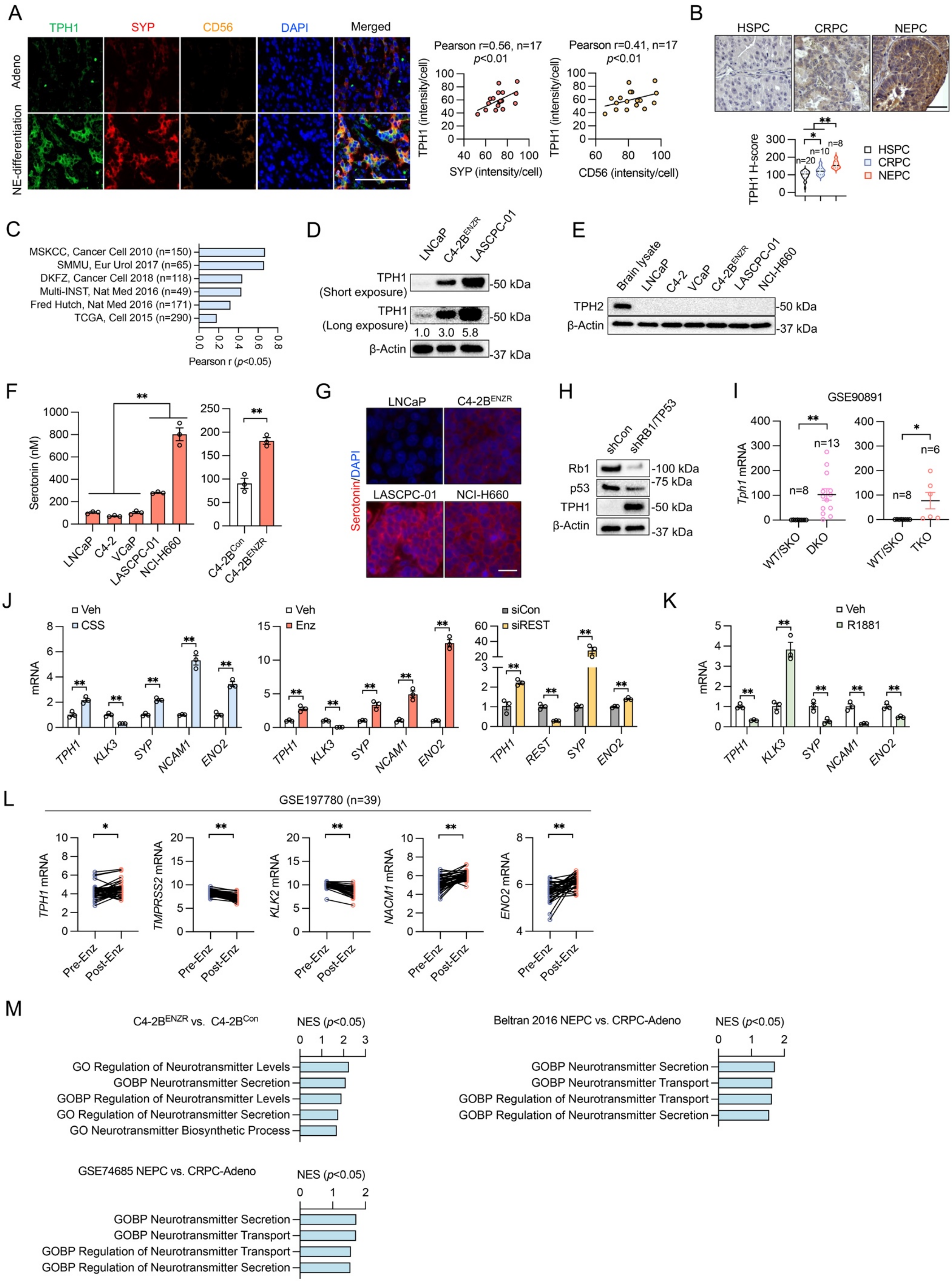

A
TPH1
SYP
CD56
DAPI
Merged
Adeno
NE-differentiation
Pearson r=0.56, n=17
p<0.01
TPH1 (intensity/cell)
SYP (intensity/cell)
Pearson r=0.41, n=17
p<0.01
CD56 (intensity/cell)
B
HSPC
CRPC
NEPC
TPH1 H-score
n=20
n=10
n=8
C
MSKCC, Cancer Cell 2010 (n=150)
SMMU, Eur Urol 2017 (n=65)
DKFZ, Cancer Cell 2018 (n=118)
Multi-INST, Nat Med 2016 (n=49)
Fred Hutch, Nat Med 2016 (n=171)
TCGA, Cell 2015 (n=290)
Pearson r (p<0.05)
D
LNCaP
C4-2B^ENZR
LASCPC-01
TPH1 (Short exposure)
TPH1 (Long exposure)
1.0 3.0 5.8
β-Actin
-50 kDa
-37 kDa
E
Brain lysate
LNCaP
C4-2
VCaP
C4-2B^ENZR
LASCPC-01
NCI-H660
TPH2
β-Actin
F
Serotonin (nM)
C4-2B^Con
G
Serotonin/DAPI
H
shCon
shRB1/TP53
Rb1
p53
TPH1
β-Actin
-100 kDa
-75 kDa
I
GSE90891
Tph1 mRNA
n=8
n=13
n=6
WT/SKO
DKO
TKO
J
Veh
CSS
Enz
siCon
siREST
mRNA
TPH1
KLK3
SYP
NCAM1
ENO2
REST
K
R1881
L
GSE197780 (n=39)
TPH1 mRNA
TMPRSS2 mRNA
KLK2 mRNA
NACM1 mRNA
ENO2 mRNA
Pre-Enz
Post-Enz
M
C4-2B^ENZR vs. C4-2B^Con
NES (p<0.05)
GO Regulation of Neurotransmitter Levels
GOBP Neurotransmitter Secretion
GOBP Regulation of Neurotransmitter Levels
GO Regulation of Neurotransmitter Secretion
GO Neurotransmitter Biosynthetic Process
Beltran 2016 NEPC vs. CRPC-Adeno
GOBP Neurotransmitter Transport
GOBP Regulation of Neurotransmitter Transport
GOBP Regulation of Neurotransmitter Secretion
GSE74685 NEPC vs. CRPC-Adeno

Supplemental Figure 1. TPH1 expression and associated serotonin levels are elevated in human NEPC. (A) Representative multiplex IF images and corresponding Pearson correlation analysis of TPH1 with SYP or CD56 expression using average per-cell staining intensity from a CRPC-NE patient cohort (n=17) as shown in Fig. 1B. IF staining in NE-differentiated vs. Adeno areas within a representative CRPC-NE patient sample is shown. Scale bar: 100 μm. **(B)** Representative images and quantification of TPH1 IHC staining in a LuCaP PDX TMA containing HSPC (n=20), CRPC (n=10), and NEPC (n=8). Scale bar: 50 μm. **(C)** Co-expression correlation between *TPH1* and *CHGA* mRNA in six independent cBioPortal datasets. **(D)** Western blot of TPH1 in LNCaP, C4-2B^{ENZR}, and LASCPC-01 cells under short and long exposures, with the normalized TPH1 protein expression in LNCaP cells set as 1. **(E)** Western blot of TPH2 in a panel of human PC cell lines as indicated, with mouse brain tissue lysate as a positive control. **(F)** ELISA of serotonin levels in the culture media of human PC cell lines corresponding to Fig. 1E (n=3). **(G)** Representative images of serotonin IF staining in LNCaP, C4-2B^{ENZR}, LASCPC-01, and NCI-H660 s.c. tumor xenografts. Scale bar: 20 μm. **(H)** Western blot of Rb1, p53, and TPH1 in control and Rb1/p53 DKD LNCaP cells. **(I)** *Tph1* mRNA in mouse prostate tumors harvested from WT, PBCre4:Pten$^{fl/fl}$ (single Pten KO, SKO), PBCre4:Pten$^{fl/fl}$:Rb1$^{fl/fl}$ (Pten/Rb1 double KO, DKO), and PBCre4:Pten$^{fl/fl}$:Rb1$^{fl/fl}$:Trp53$^{fl/fl}$ (Pten/Rb1/Trp53 triple KO, TKO) mice in GSE90891. **(J)** qPCR of *TPH1*, *KLK3*, and NE markers as indicated in LNCaP cells treated with 5% charcoal-stripped serum (CSS, 5 days), Enz (5 μM, 7 days), or *REST* siRNA (n=3). **(K)** qPCR of *TPH1, KLK3,* and NE markers in LNCaP cells treated with R1881 (10 nM, 24 hrs) (n=3). **(L)** Comparisons of *TPH1* and AR/NE marker mRNA levels in human high-grade prostate tumors collected longitudinally post- vs. pre-treatment with 3-month Enz (GSE197780, n=39). **(M)** GSEA of neurotransmitter-related gene sets enriched in C4-2B^{ENZR} vs. control cells, and in NEPC vs. CRPC-Adeno patient samples (Beltran 2016 and GSE74685). Data represent mean ± SEM. Statistics were calculated using Pearson's correlation coefficient *t*-test (A and C), one-way ANOVA with Tukey's test (B and F, left panel), unpaired two-tailed Student's *t*-test (F, right panel; I; J; and K), paired two-tailed Student's *t*-test (L), or permutation (M). *$p<0.05$, **$p<0.01$.

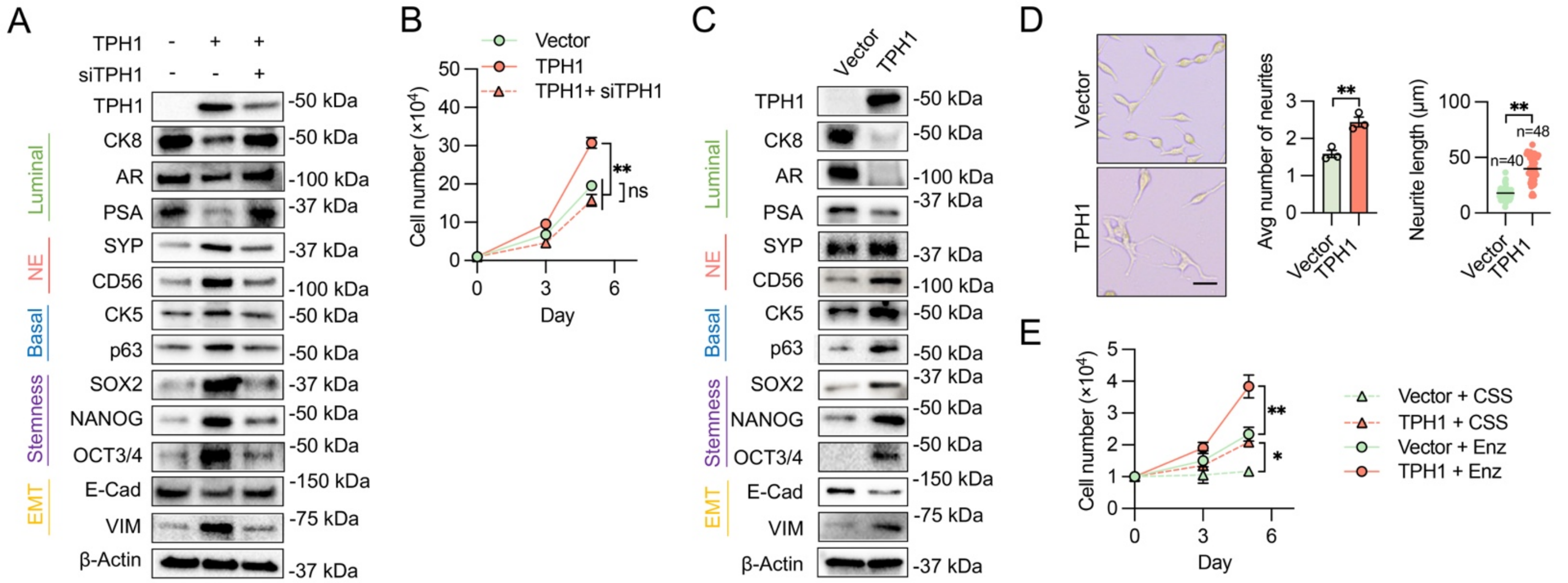

Supplemental Figure 2. TPH1 promotes NE plasticity in AdPC cells. (A) Western blot of TPH1, luminal, NE, basal, stemness, and EMT markers in control, TPH1-OE, and TPH1-OE/*TPH1* siRNA-transfected LNCaP cells. **(B)** Proliferation of control, TPH1-OE, and TPH1-OE/*TPH1* siRNA-transfected LNCaP cells for 5 days (n=3). **(C)** Western blot of TPH1, luminal, NE, basal, stemness, and EMT markers in control and TPH1-OE C4-2 cells. **(D)** Representative images of control and TPH1-OE C4-2 cell morphology and quantification of per-cell neurite number and length in each group (n=3, with >40 cells per group), representing three independent experiments. Scale bar: 20 µm. **(E)** Proliferation of control and TPH1-OE C4-2 cells treated with 5% CSS or 10 μM Enz for 5 days (n=3). Data represent mean ± SEM. Statistics were calculated using one-way ANOVA with Tukey's test (B), unpaired two-tailed Student's *t*-test (D), or two-way ANOVA with Sidak's test (E). ***p*<0.01; ns, not significant.

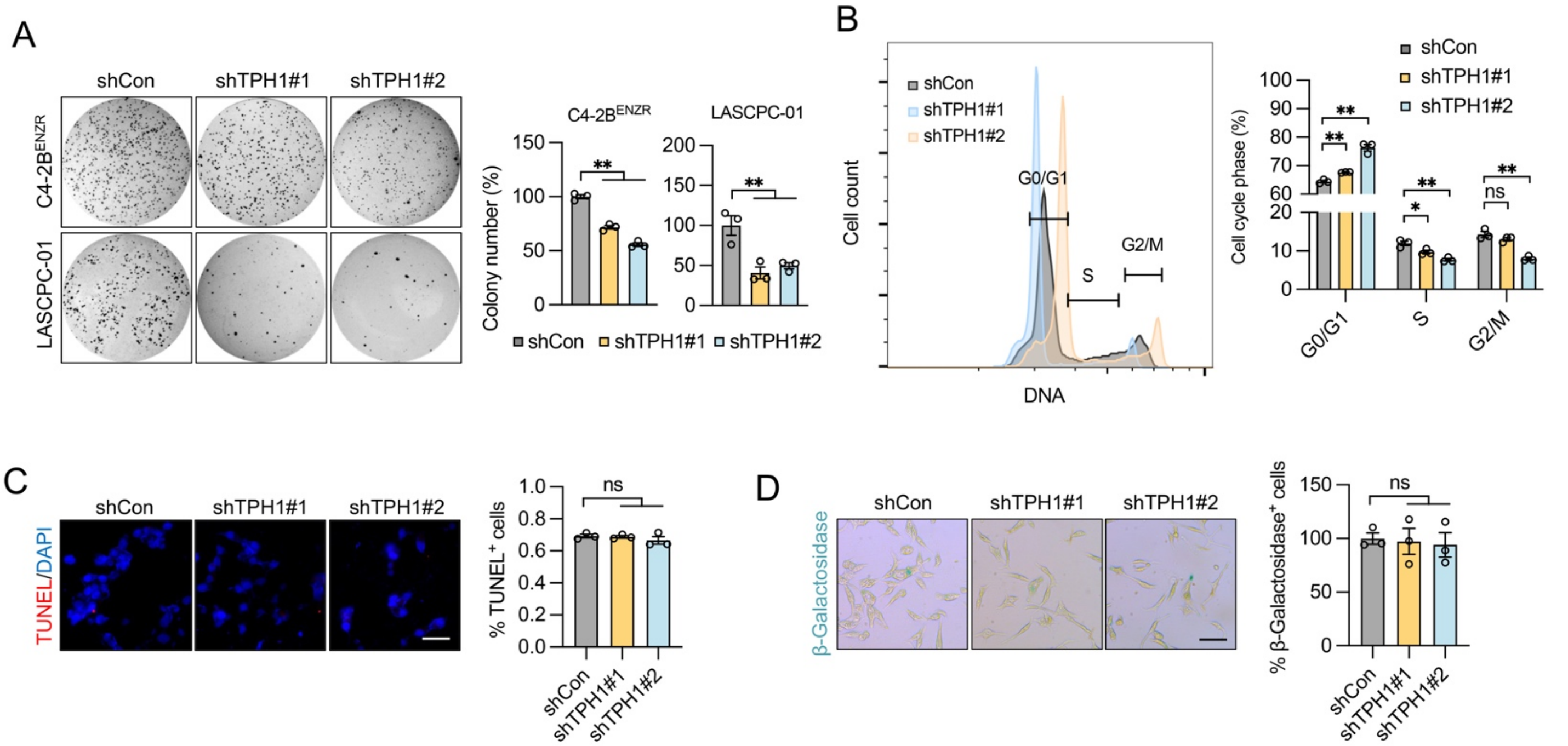

Supplemental Figure 3. TPH1 silencing inhibits colony formation and cell cycle progression but has no effect on apoptosis and senescence in NEPC cells. (A) Representative images and quantification of colony formation in soft agar by control and TPH1-KD NEPC cells (n=3). **(B)** Flow cytometric analysis of cell cycle phase distribution in control and TPH1-KD LASCPC-01cells (n=3). **(C)** Representative TUNEL staining images and quantification of TUNEL+ cells in control and TPH1-KD C4-2B^{ENZR} cells, with red fluorescence indicating apoptotic cells (n=3). Scale bar: 50 µm. **(D)** Representative β-galactosidase staining images and quantification of β-galactosidase+ cells in control and TPH1-KD C4-2B^{ENZR} cells, with cells stained in blue indicating senescent cells (n=3). Scale bar: 50 µm. Data represent mean ± SEM. Statistics were calculated using one-way ANOVA with Dunnett's test (A-D). *p<0.05, **p<0.01; ns, not significant.

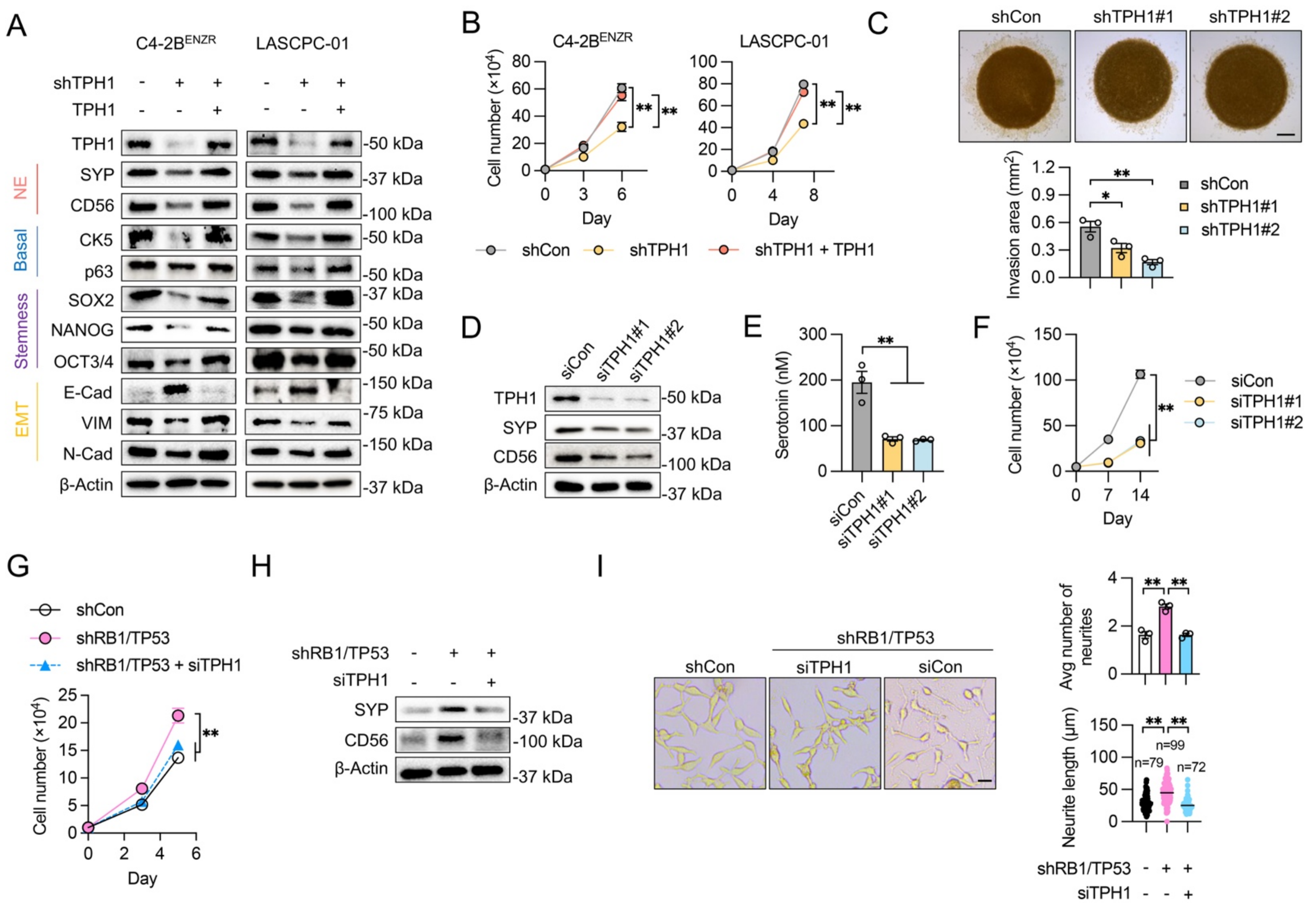

Supplemental Figure 4. TPH1 KD reduces NE marker expression and aggressive phenotypes in NEPC cells. (A) Western blot of TPH1, NE, basal, stemness, and EMT markers in control, TPH1-KD, and TPH1-KD/TPH1-reexpressing NEPC cells. **(B)** Proliferation of control, TPH1-KD, TPH1-KD/TPH1-reexpressing NEPC cells (n=3). **(C)** Representative images and quantification of 3D spheroid cell invasion by control and TPH1-KD C4-2B^{ENZR} cells (n=3). Scale bar: 400 μm. **(D)** Western blot of TPH1, SYP, and CD56 in control and *TPH1* siRNA-transfected NCI-H660 cells. **(E)** ELISA of serotonin levels in the culture media of control and *TPH1* siRNA-transfected NCI-H660 cells (n=3). **(F)** Proliferation of control and *TPH1* siRNA-transfected NCI-H660 cells for 14 days (n=3). **(G)** Proliferation of control and Rb1/p53 DKD LNCaP cells treated with *TPH1* siRNA for 5 days (n=3). **(H)** Western blot of SYP and CD56 in control and Rb1/p53 DKD LNCaP cells treated with *TPH1* siRNA. **(I)** Representative morphological images of control and Rb1/p53 DKD LNCaP cells treated with *TPH1* siRNA and quantification of per-cell neurite number and length in each group (n=3, with >70 cells per group), representing three independent experiments. Scale bar: 20 μm. Statistics were calculated using one-way ANOVA with Dunnett's test (B, C, E, F, and G) or Tukey's test (I). Data represent mean ± SEM. *p<0.05, **p<0.01.

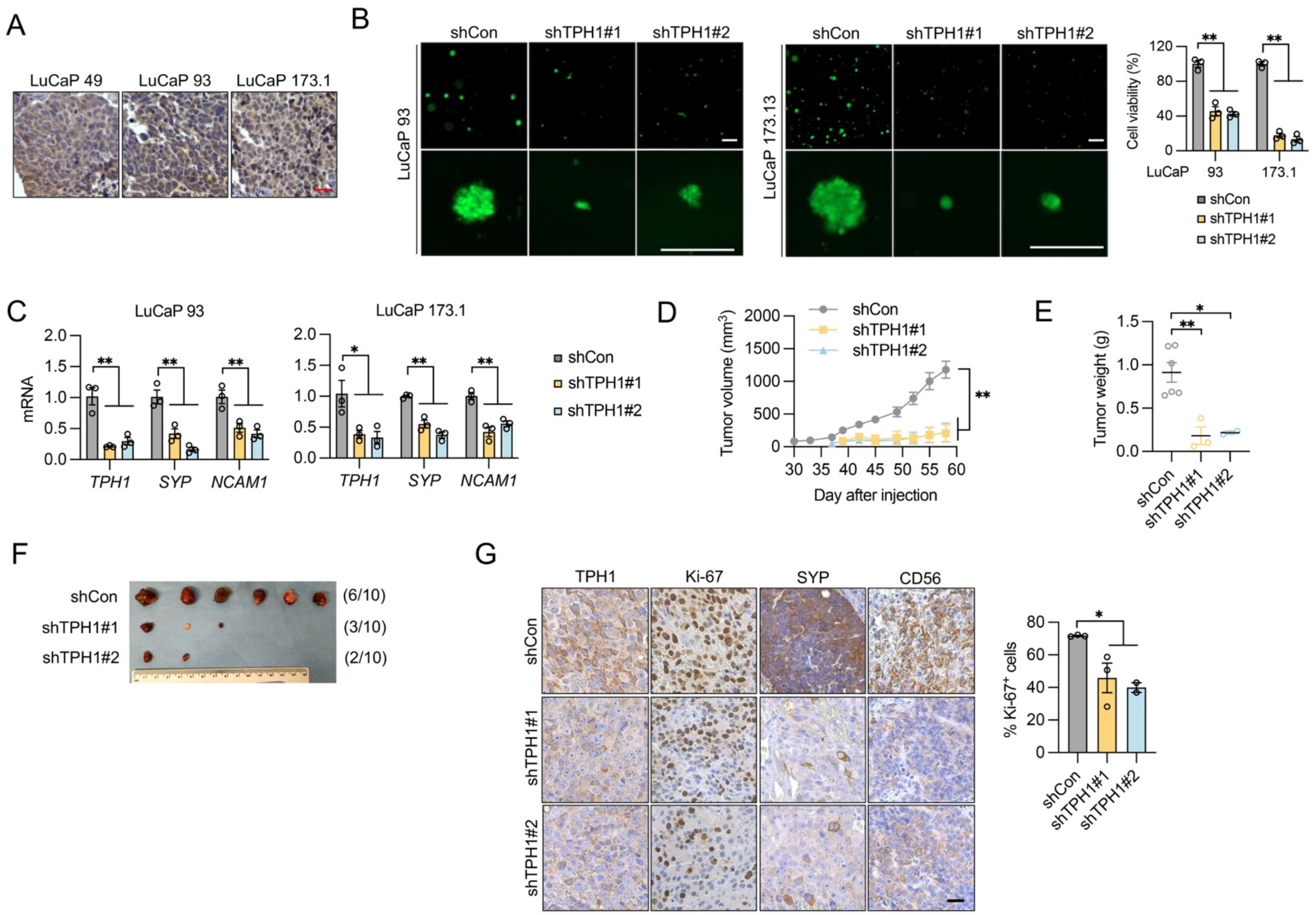

Supplemental Figure 5. TPH1 silencing inhibits the growth and NE marker expression in NEPC organoids and tumors. (A) Representative images of TPH1 IHC staining in LuCaP 49, 93, and 173.1 PDXs. Scale bar: 20 µm. **(B)** Representative fluorescence microscopic images and viability of control and TPH1-KD LuCaP 93 and 173.1 PDX-derived organoids (n=3). Scale bars: 100 µm. **(C)** qPCR of *TPH1, SYP,* and *NCAM1* in control and TPH1-KD organoids as in (B) (n=3). **(D-F)** Tumor growth curves (D), endpoint tumor weights (E), and anatomic tumor image (F) of control (shCon) and TPH1-KD (shTPH1) C4-2B^{ENZR} xenografts grown s.c. in male NSG mice (n=6, 3, and 2 tumors for shCon, shTPH1#1, and shTPH1#2 groups, respectively, with initial 10 tumor inoculations per group). **(G)** Representative IHC staining of TPH1, Ki-67, SYP, and CD56 and quantification of % of Ki-67+ cells (n=3) in TPH1-KD vs. control tumors. Scale bar: 20 µm. Data represent mean ± SEM. Statistics were calculated using one-way ANOVA with Dunnett's test (B-E and G). *p<0.05, **p <0.01.

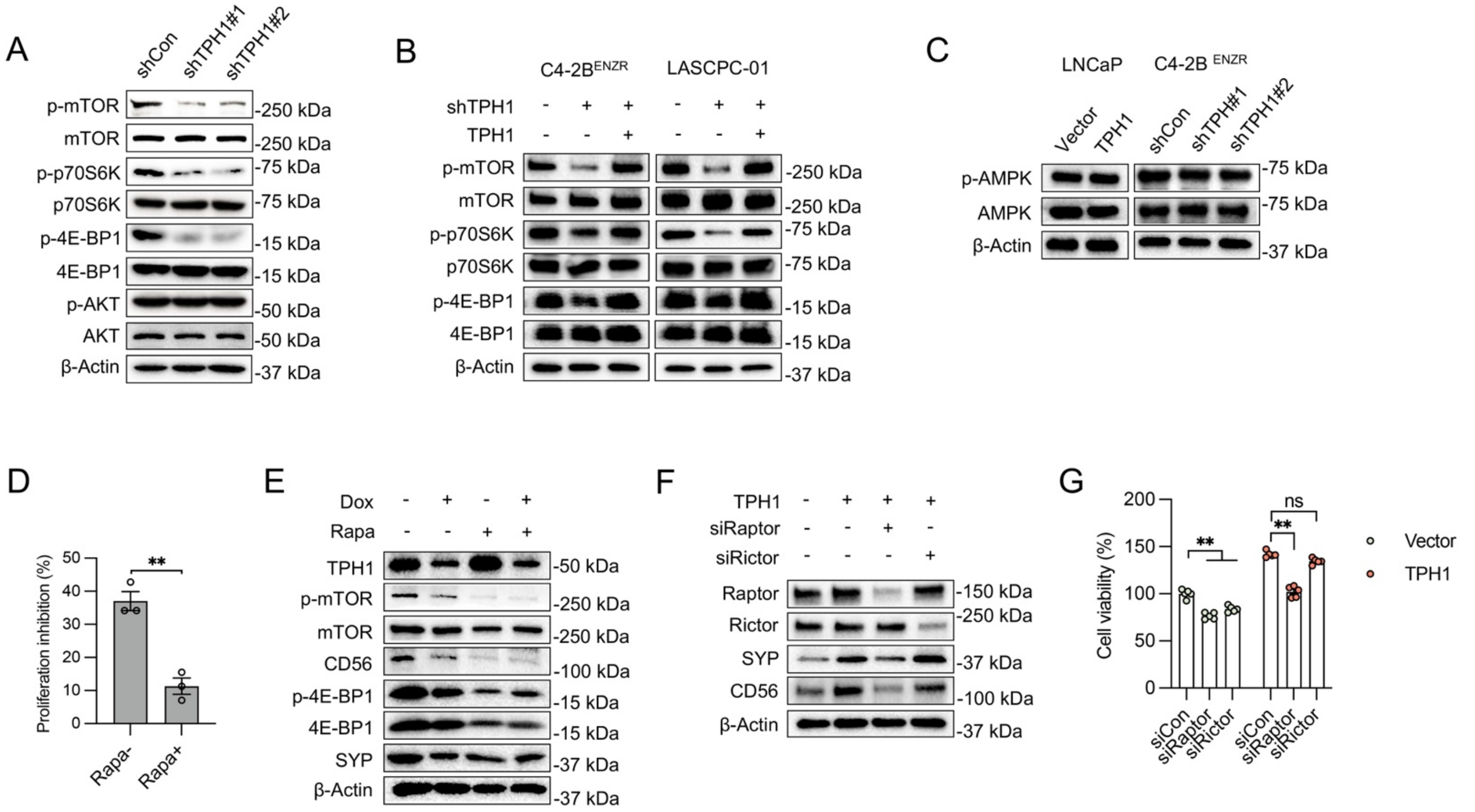

Supplemental Figure 6. TPH1 functions in NEPC cells via mTOR activation. (A) Western blot of TPH1, mTOR, and downstream phosphoproteins in control and TPH1-KD C4-2B^{ENZR} cells. **(B)** Western blot of TPH1, mTOR, and downstream phosphoproteins in control, TPH1-KD, and TPH1-KD/TPH1-reepxressing NEPC cells. **(C)** Western blot of p-AMPK in control and TPH1-manipulated PC cells under glucose-deprived conditions. **(D)** Proliferation of Dox-inducible TPH1-KD C4-2B^{ENZR} cells pre-treated with rapamycin for 24 hrs followed by Dox stimulation (100 ng/ml, 3 days) (n=3). **(E)** Western blot of TPH1, p-mTOR, p-4E-BP1, and NE markers in C4-2B^{ENZR} cells as in (D). **(F)** Western blot of Raptor, Rictor, SYP, and CD56 in control and TPH1-OE LNCaP cells treated with siRNAs against Raptor or Rictor. **(G)** Proliferation of control and TPH1-OE LNCaP cells treated with siRNAs against Raptor or Rictor for 48 hrs (n=5). Data represent mean ± SEM. Statistics were calculated using unpaired two-tailed Student's *t*-test (D) or one-way ANOVA with Dunnett's test (G). ***p*<0.01; ns, not significant.

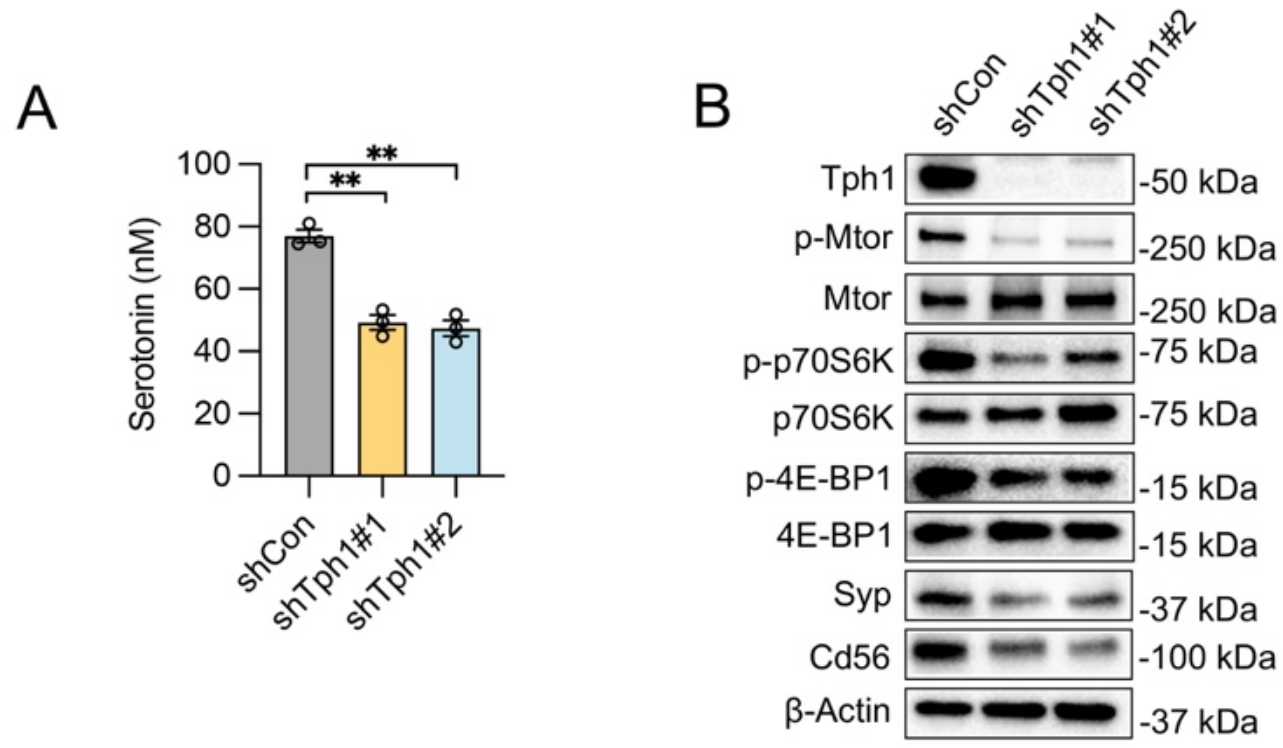

Supplemental Figure 7. Tph1 maintains Mtor signaling and NE marker expression in mouse NEPC cells. **(A)** ELISA of serotonin levels in the culture media of control and Tph1-KD TRAMP-C2 cells (n=3). **(B)** Western blot of Tph1, Mtor and downstream phosphoproteins, and NE markers in control and Tph1-KD TRAMP-C2 cells. Data represent mean ± SEM. Statistics were calculated using one-way ANOVA with Dunnett's test (A). ***p*<0.01.

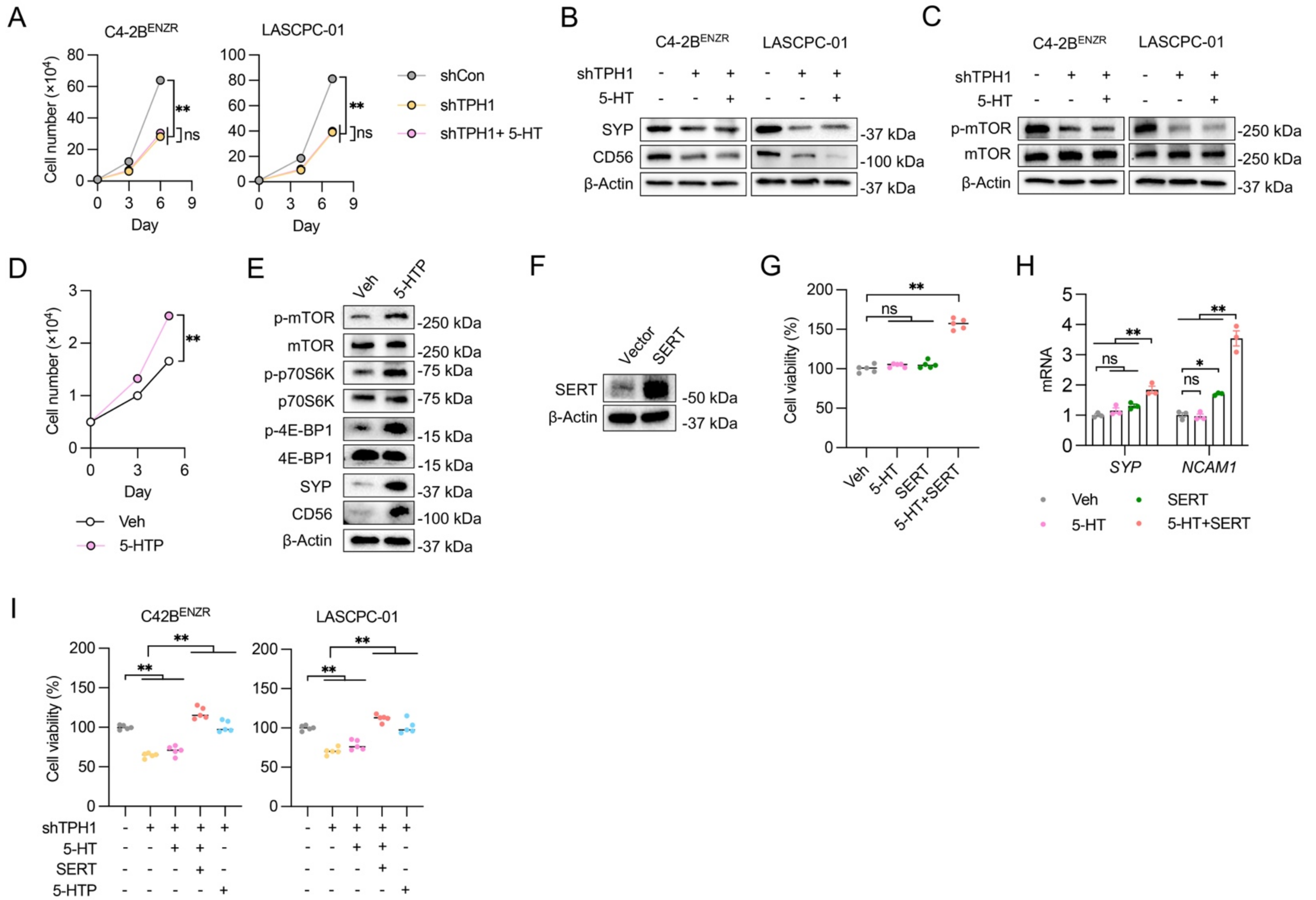

Supplemental Figure 8. TPH1's effects in NEPC cells are phenocopied by 5-HTP or by SERT-facilitated exogenous serotonin. (A) Proliferation of control and TPH1-KD NEPC cells with 10 μM 5-HT (n=3). **(B)** Western blot of NE markers in control and TPH1-KD NEPC cells with 5-HT for 48 hrs. **(C)** Western blot of p-mTOR in control and TPH1-KD NEPC cells with 5-HT for 30 min. **(D)** Proliferation of LNCaP cells with 5 μM 5-HTP (n=3). **(E)** Western blot of mTOR and downstream phosphoproteins and NE markers in LNCaP cells treated with 5-HTP for 48 hrs. **(F)** Western blot of SERT in control and SERT-transfected LNCaP cells. **(G)** Proliferation of LNCaP cells treated with 5-HT for 3 days, in the absence or presence of transfected SERT expression (n=5). **(H)** qPCR of *SYP* and *NCAM1* in LNCaP cells under the treatment as in (G) (n=3). **(I)** Proliferation of control and TPH1-KD NEPC cells treated with 5-HTP or 5-HT for 3 days, in the absence or presence of transfected SERT expression. Data represent mean ± SEM. Statistics were calculated using one-way ANOVA with Tukey's test (A and G-I) or unpaired two-tailed Student's *t*-test (D). *$p<0.05$, **$p<0.01$; ns, not significant.

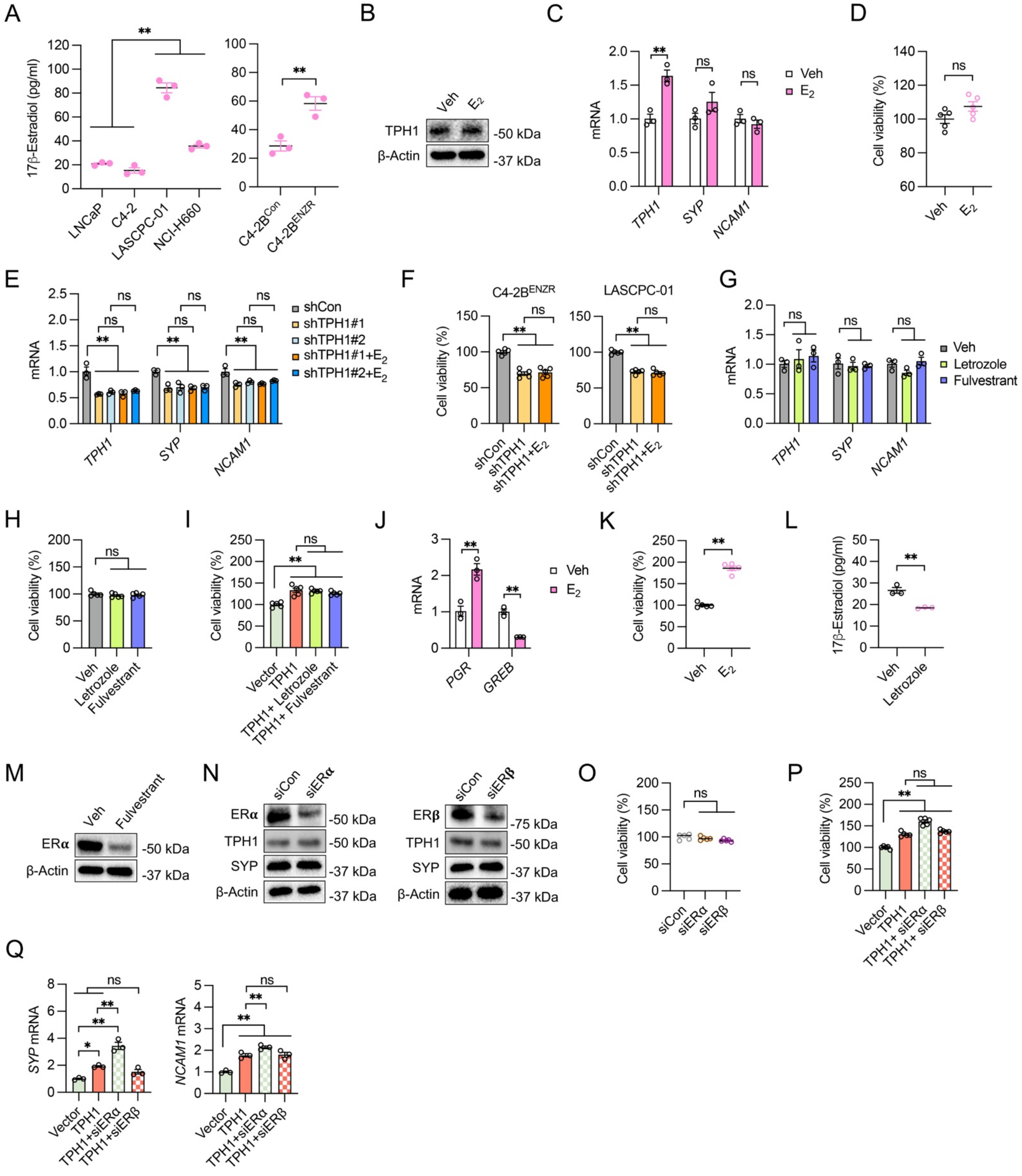
A
17β-Estradiol (pg/ml)
LNCaP
C4-2
LASCPC-01
NCI-H660
C4-2B^Con
C4-2B^ENZR
B
Veh
E2
TPH1
β-Actin
-50 kDa
-37 kDa
C
mRNA
TPH1
SYP
NCAM1
Veh
E2
D
Cell viability (%)
E
shCon
shTPH1#1
shTPH1#2
shTPH1#1+E2
shTPH1#2+E2
F
C4-2B^ENZR
LASCPC-01
shTPH1
shTPH1+E2
G
Letrozole
Fulvestrant
H
I
Vector
TPH1+ Letrozole
TPH1+ Fulvestrant
J
PGR
GREB
K
L
M
ERα
N
siCon
siERα
siERβ
ERβ
SYP
-75 kDa
O
P
TPH1+ siERα
TPH1+ siERβ
Q
SYP mRNA
NCAM1 mRNA
ns
*
**

Supplemental Figure 9. TPH1 appears to function in NEPC cells independent of estrogen signaling. (A) ELISA of 17β-estradiol levels in the culture media of human PC cell lines as indicated (n=3). **(B)** Western blot of TPH1 in LNCaP cells treated with E_2 (10 nM, 48 hrs). **(C)** qPCR of *TPH1*, *SYP*, and *NCAM1* in LNCaP cells treated with E_2 for 48 hrs (n=3). **(D)** Proliferation of LNCaP cells treated with E_2 for 3 days (n=5). **(E)** qPCR of *TPH1*, *SYP*, and *NCAM1* in control and TPH1-KD C4-2B^{ENZR} cells treated with E_2 for 3 days (n=3). **(F)** Proliferation of control and TPH1-KD NEPC cells treated with E_2 for 3 days (n=5). **(G)** qPCR of *TPH1*, *SYP*, and *NCAM1* in C4-2B^{ENZR} cells treated with 100 nM letrozole or 100 nM fulvestrant for 48 hrs (n=3). **(H)** Proliferation of C4-2B^{ENZR} cells treated with letrozole or fulvestrant for 3 days (n=5). **(I)** Proliferation of control and TPH1-OE LNCaP cells treated with letrozole or fulvestrant for 3 days (n=5). **(J)** qPCR of *PGR* and *GREB* in MCF-7 cells treated with E_2 for 48 hrs (n=3). **(K)** Proliferation of MCF-7 cells treated with E_2 for 3 days (n=5). **(L)** ELISA of 17β-estradiol levels in the culture media of MCF-7 cells treated with letrozole for 48 hrs (n=3). **(M)** Western blot of ERα in MCF-7 cells treated with fulvestrant for 48 hrs. **(N)** Western blot of ERα, ERβ, TPH1, and SYP in C4-2B^{ENZR} cells treated with siRNAs against ERα or ERβ. **(O)** Proliferation of C4-2B^{ENZR} cells treated with siRNAs against ERα or ERβ (n=5). **(P)** Proliferation of control and TPH1-OE LNCaP cells treated with siRNAs against ERα or ERβ (n=5). **(Q)** qPCR of *SYP* and *NCAM1* in control and TPH1-OE LNCaP cells treated with siRNAs against ERα or ERβ (n=3). Data represent mean ± SEM. Statistics were calculated using one-way ANOVA with Tukey's test (A, left panel; E; F; I; P; and Q), unpaired two-tailed Student's *t*-test (A, right panel; C; D; J; K; and L), or one-way ANOVA with Dunnett's test (G, H, and O). *p<0.05, **p <0.01; ns, not significant.

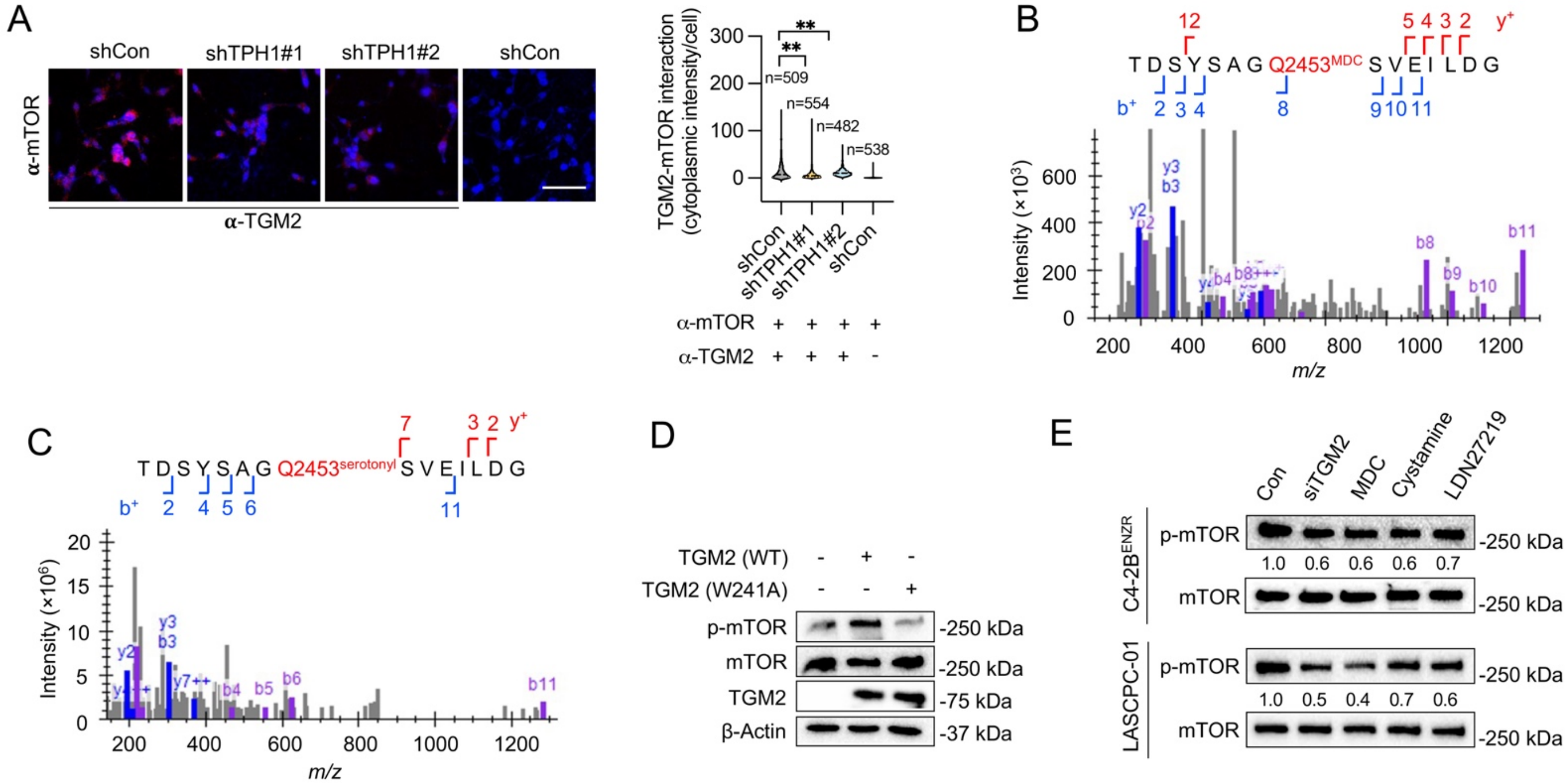

Supplemental Figure 10. mTOR is activated via TGM2-mediated serotonylation. (A) Representative proximity ligation assay staining and quantification of mTOR-TGM2 interaction by per-cell cytoplasmic fluorescence intensity in control and TPH1-KD C4-2B^{ENZR} cells. Scale bar: 50 μm. **(B, C)** LC-MS/MS analysis of TGM2-transamidated MDC (B) or serotonin (C) to mTOR peptide (aa 2446-2460). **(D)** Western blot of p-mTOR and TGM2 in TPH1-OE LNCaP cells expressing WT or mutant (W241A) TGM2. **(E)** Western blot of p-mTOR in NEPC cells with *TGM2* siRNA (48 hrs), MDC (200 μM, 1 hr), cystamine (500 μM, 1 hr), or LDN27219 (1 μM, 1 hr), with the normalized p-mTOR levels in untreated cells set as 1. Data represent mean ± SEM. Statistics were calculated using one-way ANOVA with Dunnett's test (A). $^{**}p<0.01$.

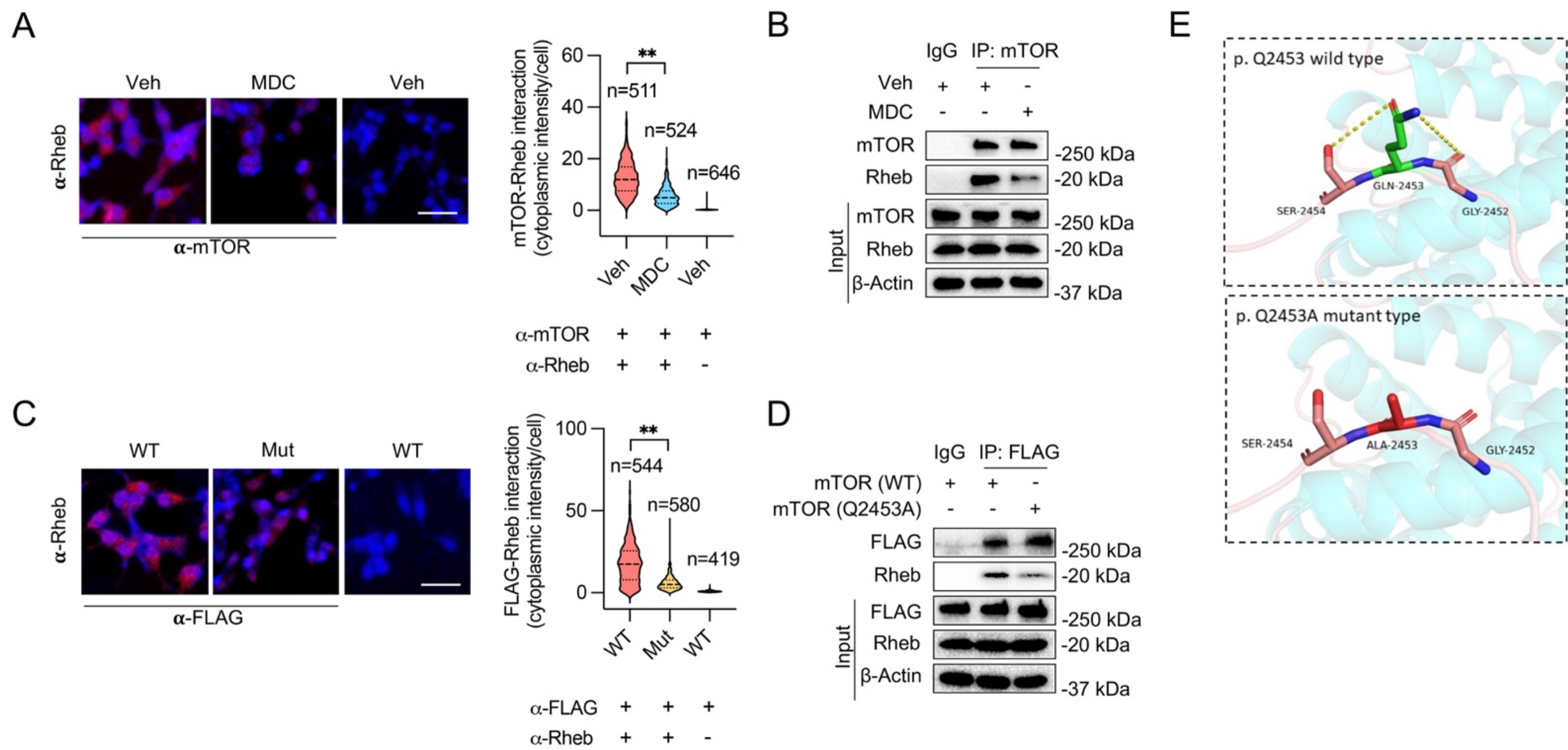

Supplemental Figure 11. TPH1 enhances Rheb-mTOR interaction via serotonylation at Gln2453. (A) Representative proximity ligation assay staining and quantification of Rheb-mTOR interaction by per-cell cytoplasmic fluorescence intensity in control and MDC (200 μM, 24 hrs)-treated C4-2B^{ENZR} cells. Scale bar: 50 μm. **(B)** Co-IP assay of Rheb-mTOR interaction in control and MDC-treated LNCaP cells under 5 μM 5-HTP stimulation. **(C)** Representative proximity ligation assay staining and quantification of Rheb interaction with transfected FLAG-tagged WT or mutant (Q2453A) mTOR by per-cell cytoplasmic fluorescence intensity in C4-2B^{ENZR} cells. Scale bar: 50 μm. **(D)** Co-IP assay of Rheb interaction with transfected FLAG-tagged WT or mutant mTOR in LASCPC-01 cells. **(E)** 3D PyMOL visualization of regional conformations of WT and mutant mTOR (aa 2452-2454), with hydrogen bonds shown as dashed lines in the WT model. Data represent mean ± SEM. Statistics were calculated using one-way ANOVA with Dunnett's test (A and C). ***p*<0.01.

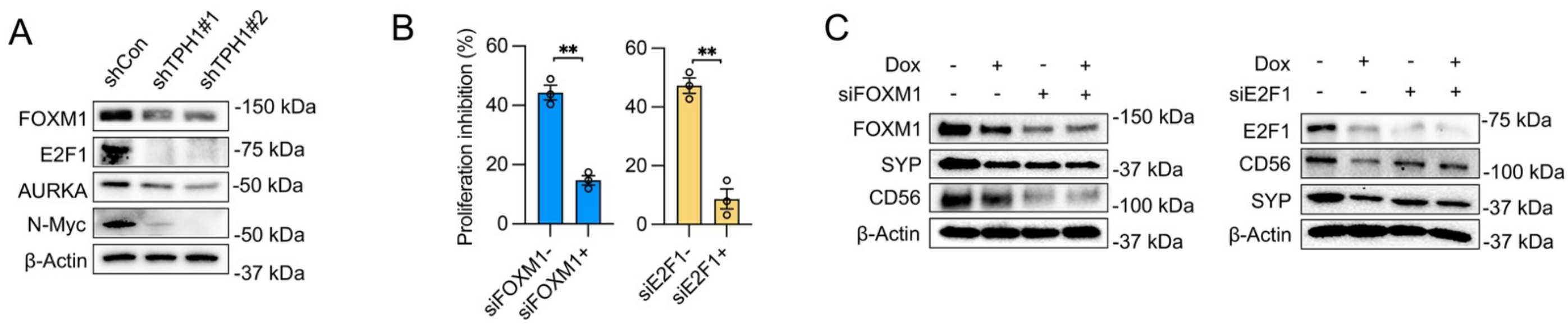

Supplemental Figure 12. FOXM1 and E2F1 mediate TPH1 function in NEPC cells. (A) Western blot of FOXM1, E2F1, AURKA, and N-Myc in control and TPH1-KD C4-2B^{ENZR} cells. **(B)** Proliferation of Dox-inducible TPH1-KD C4-2B^{ENZR} cells pre-treated with *FOXM1* or *E2F1* siRNAs for 24 hrs followed by Dox stimulation (100 ng/ml, 3 days) (n=3), with the % proliferation inhibition shown. **(C)** Western blot of FOXM1, E2F1, SYP, and CD56 in Dox-inducible TPH1-KD C4-2B^{ENZR} cells under the treatment as in (B). Data represent mean ± SEM. Statistics were calculated using unpaired two-tailed Student's *t*-test (B). $^{**}p<0.01$.

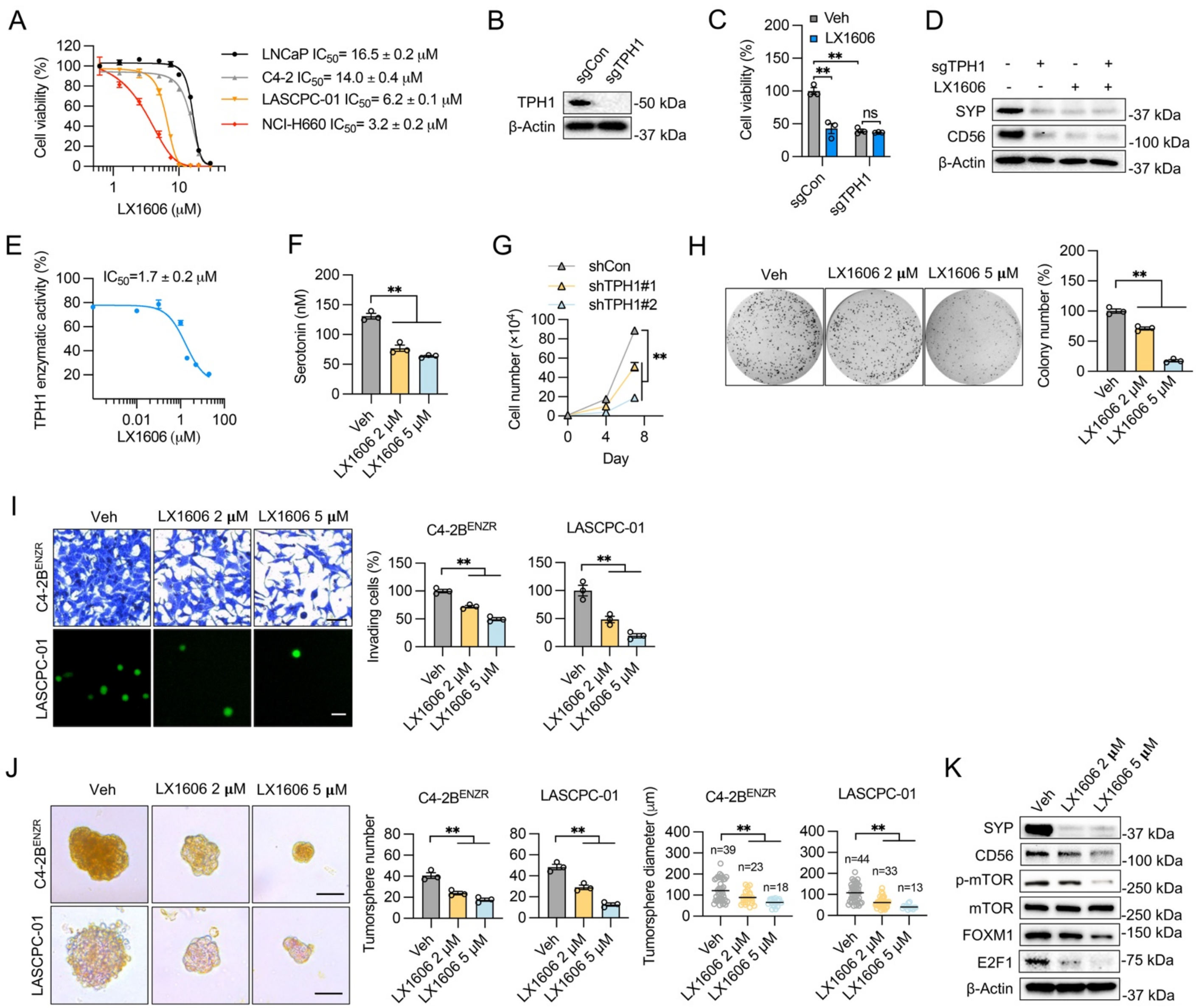

Supplemental Figure 13. LX1606 demonstrates TPH1-specific targeting and inhibits the aggressive behavior of NEPC cells. (A) MTS cell viability assays of the indicated PC cell lines treated with LX1606 at various concentrations for 3 days (n=3), with IC_{50} values denoted. **(B)** Western blot of TPH1 in control (sgCon) and TPH1-KO (sgRNA) pooled C4-2B^{ENZR} cells. **(C)** Proliferation of control and TPH1-KO C4-2B^{ENZR} cells treated with LX1606 (5 µM, 3 days) (n=3). **(D)** Western blot of SYP and CD56 in control and TPH1-KO C4-2B^{ENZR} cells under the same treatment in (C). **(E)** TPH1 enzymatic activity assay of inhibitory efficacy of LX1606 at various concentrations, with IC_{50} value denoted (n=3). **(F)** ELISA of serotonin levels in the culture media of C4-2B^{ENZR} cells treated with LX1606 for 48 hrs (n=3). **(G)** Proliferation of C4-2B^{ENZR} cells treated with LX1606 (n=3). **(H)** Representative images and quantification of soft-agar colony formation by C4-2B^{ENZR} cells treated with LX1606 (n=3). **(I)** Representative images and quantification of cell invasion by control and TPH1-KD NEPC cells treated with LX1606 (n=3). Scale bars: 50 µm. **(J)** Representative images and quantification of the number and size of tumorspheres formed by NEPC cells treated with LX1606 (n=3). Scale bars: 100 µm. **(K)** Western blot of SYP, CD56, p-mTOR, FOXM1, and E2F1 in C4-2B^{ENZR} cells treated with LX1606 for 3 days. Data represent mean ± SEM. Statistics were calculated using two-way ANOVA with Sidak's test (C) or one-way ANOVA with Dunnett's test (F-J). ***p*<0.01; ns, not significant.

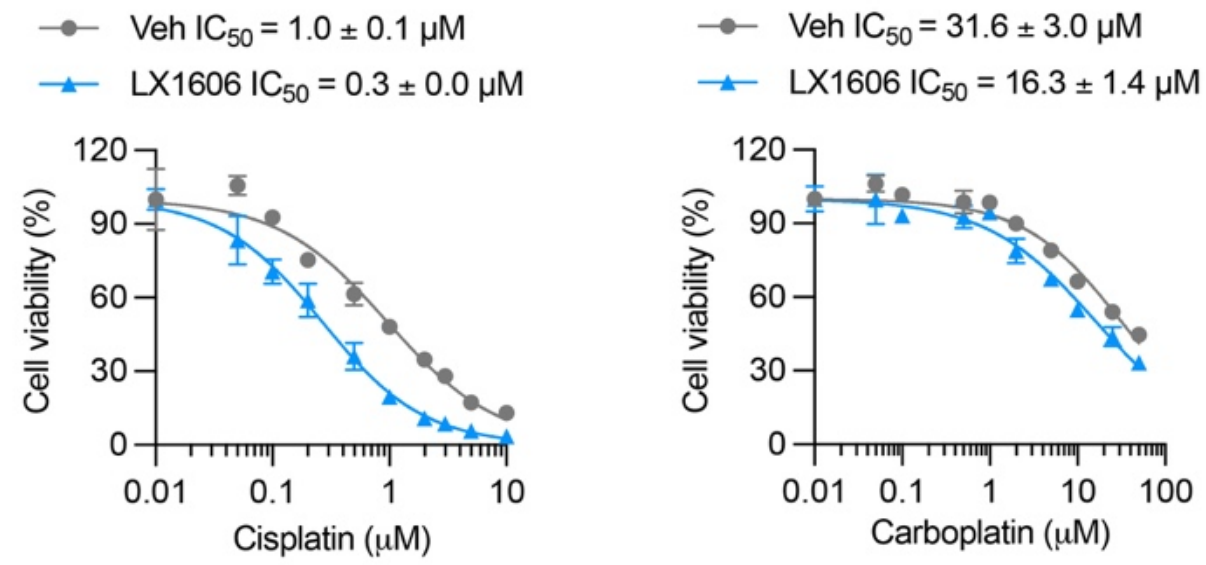

Supplemental Figure 14. LX1606 enhances the efficacy of platinum-based agents in NEPC cells. MTS cell viability assays of LASCPC-01 treated with cisplatin (left) or carboplatin (right) at various concentrations in the absence or presence of 2 μM LX1606 for 3 days (n=3), with cisplatin or carboplatin IC_{50} values denoted.

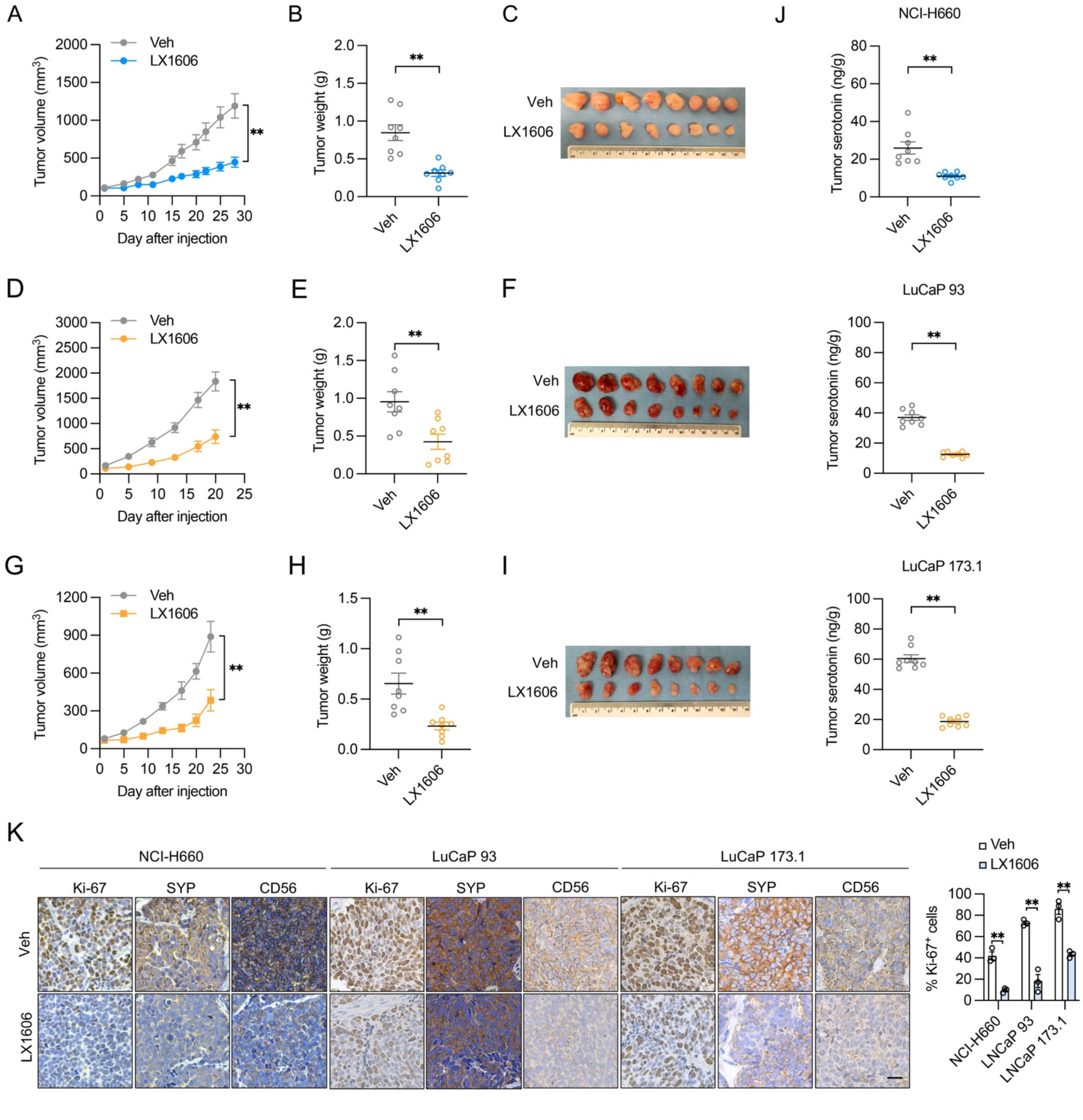

Supplemental Figure 15. LX1606 inhibits growth and NE marker expression in NEPC xenografts in mice. (A-C) Tumor growth curves (A), endpoint tumor weights (B), and anatomic tumor image (C) of NCI-H660 xenografts grown s.c. in male NSG mice receiving vehicle (Veh) or LX1606 (n=8). **(D-F)** Tumor growth curves (D), endpoint tumor weights (E), and anatomic tumor image (F) of LuCaP 93 NEPC PDXs grown s.c. in male NSG mice receiving vehicle or LX1606 (n=8). **(G-I)** Tumor growth curves (G), endpoint tumor weights (H), and anatomic tumor image (I) of LuCaP 173.1 NEPC PDXs grown s.c. in male NSG mice receiving vehicle or LX1606 (n=8). **(J)** ELISA of tumor serotonin levels in control and LX1606-treated NEPC xenografts (n=8). **(K)** Representative IHC staining of Ki-67, SYP, and CD56 and quantification of % of Ki-67+ cells (n=3) in control and LX1606-treated NEPC xenografts. Scale bar: 20 μm. Data represent mean ± SEM. Statistics were calculated using unpaired two-tailed Student's *t*-test (A, B, D, E, G, H, J, and K). ***p*<0.01.

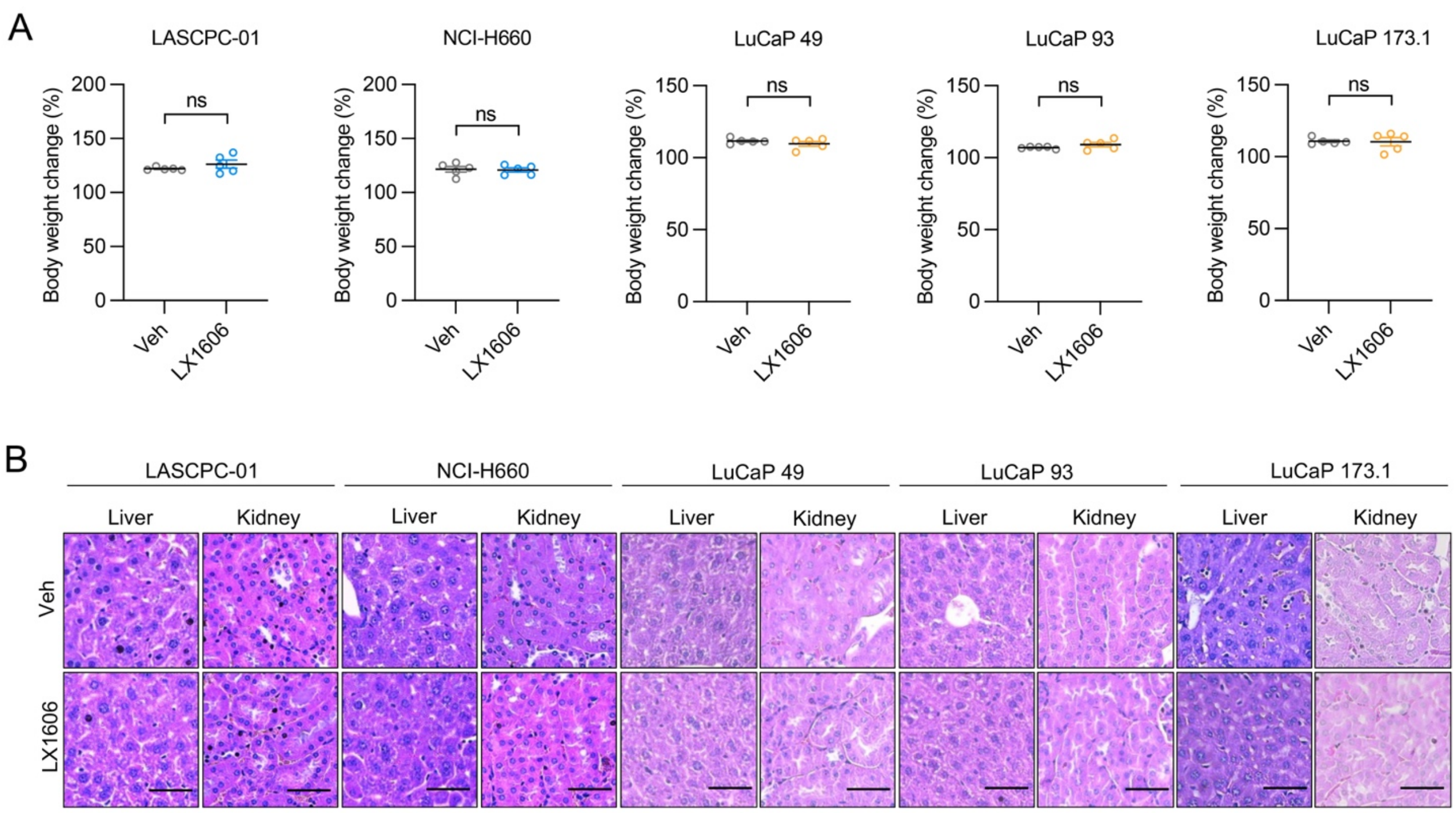

Supplemental Figure 16. LX1606 does not cause apparent toxicity to xenografted mice. (A) Percentage changes in mouse body weight at endpoints relative to pre-treatment baselines in control and LX1606-treated mice bearing NEPC tumors (n=5-8) as shown in Fig. 9 and Supplemental Fig. 15. **(B)** Representative H&E images of liver and kidneys from control and LX1606-treated mice as described in (A). Scale bars: 50 μm. Data represent mean ± SEM. Statistics were calculated using unpaired two-tailed Student's *t*-test (A). Ns, not significant.

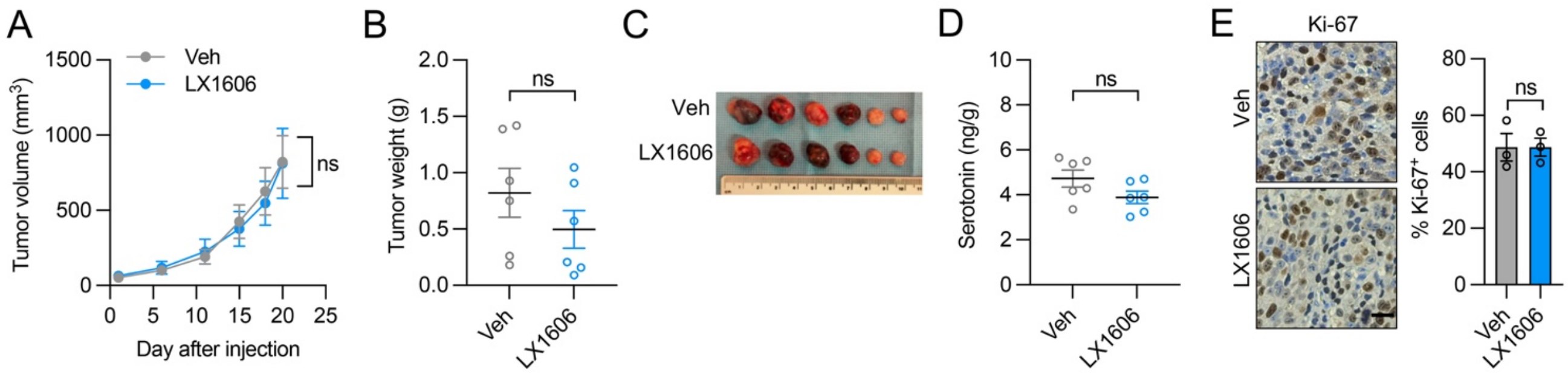

Supplemental Figure 17. LX1606 has minimal effect on the growth of TPH1-low LNCaP AdPC xenografts in mice. (A-C) Tumor growth curves (A), endpoint tumor weights (B), and anatomic tumor image (C) of LNCaP xenografts grown s.c. in male NSG mice receiving vehicle or LX1606 (n=6). **(D)** ELISA of tumor serotonin levels in control and LX1606-treated LNCaP tumors (n=6). **(E)** Representative Ki-67 IHC staining and quantification of % of Ki-67+ cells (n=3) in control and LX1606-treated LNCaP tumors. Scale bar: 20 μm. Data represent mean ± SEM. Statistics were calculated using unpaired two-tailed Student’s *t*-test (A, B, D, and E). Ns, not significant.

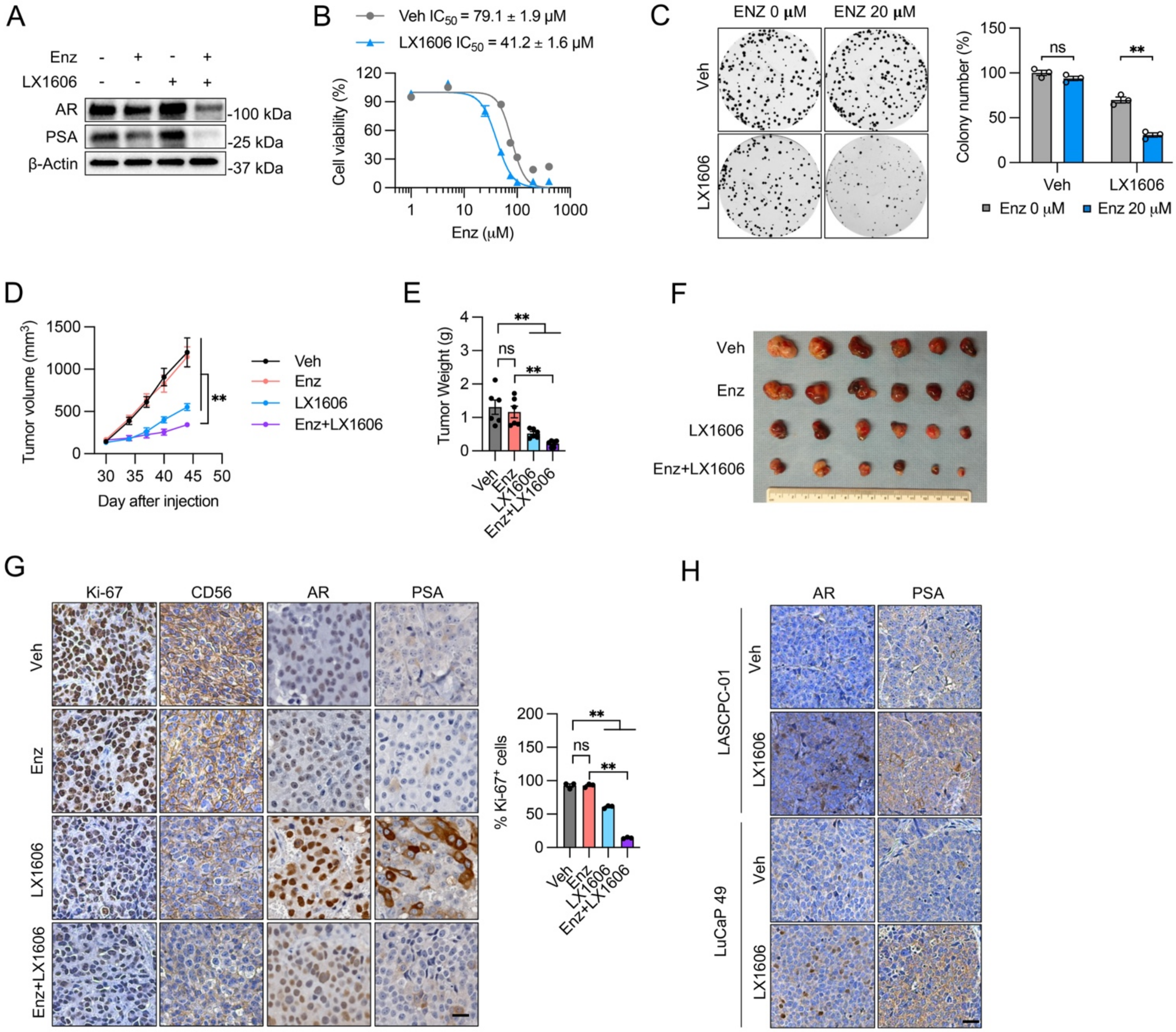

Supplemental Figure 18. LX1606 restores Enz efficacy in C4-2B^{ENZR} t-NEPC cells and xenografts in mice. (A) Western blot of AR and PSA in C4-2B^{ENZR} cells treated with 20 μM Enz and/or 2 μM LX1606 for 48 hrs. **(B)** MTS cell viability assay of C4-2B^{ENZR} with Enz at various concentrations in the absence or presence of 2 μM LX1606 for 5 days (n=3), with Enz IC_{50} values denoted. **(C)** Representative images and quantification of soft-agar colony formation by C4-2B^{ENZR} cells with 20 μM Enz and/or 2 μM LX1606 (n=3). **(D-F)** Tumor growth curves (D), endpoint tumor weights (E), and anatomic tumor image (F) of C4-2B^{ENZR} xenografts grown s.c. in male NSG mice receiving vehicle, Enz, LX1606, or both (n=6). **(G)** Representative IHC staining of Ki-67, CD56, AR, and PSA and quantification of % of Ki-67+ cells in C4-2B^{ENZR} tumors across different groups. Scale bar: 20 μm. **(H)** Representative IHC staining of AR and PSA in control and LX1606-treated NEPC tumors as shown in Fig. 9. Scale bar: 20 μm. Data represent mean ± SEM. Statistics were calculated using two-way ANOVA with Sidak's test (C) or one-way ANOVA with Tukey's test (D, E, and G). ***p*<0.01; ns, not significant.

Gene	Forward	Reverse
TPH1	TCTACCCAACCCATGCTTGC	GCCCAGACCTCCGAATTGAT
REST	TGGGGCCTGCTCCCACAGAG	GCAGGCCGTATCTGGGCACC
KLK3	CCAGAGGAGTTCTTGACCCCAAA	CCCCAGAATCACCCGAGCAG
SYP	CTACCAGCCTGACTATGG	GGGCTTCACTGACCAGAC
NCAM1	GTCCTGCTCCTGGTGGTTGT	TGACCGCAATGCACATGAA
ENO2	GCCAAAGGTCTTTTCCGG	CCTTCAGGACACCTTTGC
FOXM1	GTTTAAGCAGCAGCAGAAACGA	TCACCGGGAACTGGATAGGT
E2F1	CACAGATCCCAGCCAGTCTCTA	GAGAAGTCCTCCCGCACATG
AURKA	TACAGCTAGAGGCATCATGGAC	ACGAGAACACGTTTTGGACCT
MYCN	GAGGACACCCTGAGCGATTC	TTGGTGTTGGAGGAGGAACG
PGR	CTATCTCCCTGGACGGGCTA	AAGCGGGAATCTTCCTTGGG
GREB	TGGACAGCTGAAGACGACAC	GTGCAGCTGGAAAGGGTTTG

Supplemental Table 1. Sequences of qPCR primers.

Supplemental Methods

Plasmids and reagents.

A human *TPH1* lentiviral expression construct was generated by inserting the *TPH1* coding region into the *Xho*I/*BamH*I sites of the pLVX-AcGFP1-N1 vector (Clontech) containing a puromycin-resistance gene. A doxycycline (Dox)-inducible *TPH1* shRNA construct was generated by cloning the human *TPH1* shRNA sequence (TRCN0000056626) into the *Nhe*I/*EcoR*I sites of the EZ-Tet-pLKO-Puro vector (Addgene) containing a puromycin-resistance gene. Primer sequences for constructing *TPH1* shRNA oligomers were forward 5′-CTAGCCTGTGAATCTACCAGATAATT TACTAGTAATTATCTGGTAGATTCACAGTTTTTG-3′ and reverse 5′-AATTCAAAAACTGTGAATCTACCAGATAATTACTAGTAAATTATCTGGTAGATTCACAGG-3′. The human *TPH1* pSpCas9(BB)-2A-Puro-sgRNA construct for CRISPR/Cas9-based KO was generated by the Gene Editing Reagent Core (GERC) at Washington State University (WSU). The sgRNA target sequence was 5′-GCCAACAGAGTTCTGATGTATGG-3′, with the PAM sequence underlined. The human pcDNA3-Flag mTOR construct was a gift from Jie Chen (University of Illinois at Urbana-Champaign, Champaign, IL, USA), obtained from Addgene (plasmid # 26603), and used as a template to generate PCR fragments encoding full-length mTOR (FL, aa 1-2549) or its kinase domain (KD, aa 2181-2516). Truncated mTOR derivatives were amplified using internal *EcoR*I and *Kpn*I sites and cloned into the pCMV vector with an N-terminal 3×FLAG tag (Sigma-Aldrich). Similarly, the human pLVX-TGM2 construct, originally prepared from the pHRST-IRES-TGM2 construct provided by Jong Bae Park (National Cancer Center, Goyang, Korea), was used as a template to generate PCR fragments encoding full-length TGM2 (FL, aa 1-688) or its catalytic core domain (CCD, aa 140-454). These fragments were cloned into the *Hind*III/*Xho*I sites of the pCMV-3Tag-7 vector with an N-terminal 3×Myc tag (Agilent Technologies). Constructs lacking the mTOR kinase domain (ΔKD) or TGM2 catalytic domain (ΔCCD) in respective vectors were generated by the GERC at WSU. The human pcDNA3-SERT construct was a gift from Randy Blakely (Vanderbilt University, Nashville, TN, USA) and obtained from Addgene (plasmid # 15483). Human *TPH1* and nontarget control shRNA lentiviral particles were purchased from Sigma-Aldrich. Human *TPH1* siRNA (SR304909) and *FOXM1* siRNA (SR320176) were purchased from Origene. Human *REST* siRNA pool (sc-38129), *E2F1* siRNA pool (sc-29297), *RAPTOR* siRNA pool (sc-44069), *RICTOR* siRNA pool (sc-61478), *ESR1* siRNA pool (sc-29305), *ESR2* siRNA pool (sc-35325), and nontarget control siRNA (sc-37007) were purchased from Santa Cruz Biotechnology. Rapamycin was purchased from Selleck Chemicals. Dox, serotonin, asenapine, monodansylcadaverine (MDC), 5-hydroxytryptophan (5-HTP), and E_2 were purchased from Sigma-Aldrich. Enzalutamide (Enz), sertraline, cystamine, LDN27219, LX1606, letrozole, fulvestrant, cisplatin, and carboplatin were purchased from MedChemExpress.

Antibodies.

The antibodies used in immunoblots are listed below: TPH1 (Abcam, ab52954), AR (Cell Signaling Technology, 5153), PSA (Santa Cruz Biotechnology, sc-7638), CK8 (Developmental Studies Hybridoma Bank at the University of Iowa, TROMA-I-c), SYP (Santa Cruz Biotechnology, sc-17750), CD56 (Cell Signaling Technology, 99746), CK5 (Santa Cruz Biotechnology, sc-32721), p63 (Santa Cruz Biotechnology, sc-25268), SOX2 (Santa Cruz Biotechnology, sc-365823), NANOG (Santa Cruz Biotechnology, sc-134218), OCT3/4 (Santa Cruz Biotechnology, sc-5279), E-cadherin (Santa Cruz Biotechnology, sc-8426), vimentin (Proteintech, 103666-1-AP), N-cadherin (Santa Cruz Biotechnology, sc-7939), p-mTOR (Ser2448) (Cell Signaling Technology, 5536), mTOR (Cell Signaling Technology, 2983), p-p70S6K (Thr389) (Cell Signaling Technology, 9234), p70S6K (Cell Signaling Technology, 9202), p-4E-BP1 (Thr37/46) (Cell Signaling Technology, 2855), 4E-BP1 (Cell Signaling Technology, 9644), p-AKT (Ser473) (Cell Signaling Technology, 4060), AKT (Cell Signaling Technology, 4691), TGM2 (Proteintech, 15100-1-AP), FOXM1 (Santa Cruz Biotechnology, sc-376471), E2F1 (Cell Signaling Technology, 3742), AURKA (Cell Signaling Technology, 14475), N-Myc (Sigma-Aldrich, MABE333), TPH2 (Novus Biologicals, NB100-74555), Rb1 (Cell Signaling Technology, 9309), p53 (Cell Signaling Technology, 9282), Rheb (Cell Signaling Technology, 13879), Raptor (Cell Signaling Technology, 2280), Rictor (Cell Signaling Technology, 2114), p-AMPK (Thr172) (Cell Signaling Technology, 2535), AMPK (Cell Signaling Technology, 5831), ERα (Cell Signaling Technology, 8644), ERβ (Cell Signaling Technology, 89954), SERT (Proteintech, 19559-1-AP), and β-actin (Santa Cruz Biotechnology, sc-69879).

Analyses of cell cycle, cell apoptosis, and cell senescence.

For the cell cycle assay, cells were harvested and fixed in 70% pre-chilled ethanol at 4°C overnight. Fixed cells were then rinsed with cold phosphate-buffered saline (PBS) and incubated with 0.5 ml of propidium

iodide/ribonuclease staining buffer (BD Biosciences) at room temperature for 15 min, followed by flow cytometric analysis of cell cycle phase distribution. For the assessment of cell apoptosis, the terminal deoxynucleotidyl transferase dUTP nick end labeling (TUNEL) assay was performed. Cells were cultured on chamber slides for 24 hrs, fixed in 4% formaldehyde, permeabilized with 0.5% Triton X-100, and subjected to TUNEL staining using a fluorescein-based In Situ Cell Death Detection Kit (Roche). Nuclei were counterstained with DAPI, and images were acquired on a Zeiss Axio Imager M2 microscope and analyzed with HALO software (Indica Labs). For the cell senescence assay, cells were seeded at 5×10^4 cells/well in 24-well plates. After 48 hrs, the expression of β-galactosidase was detected using the Senescence β-galactosidase Staining Kit (Cell Signaling) following the manufacturer's instructions. The images were taken by a Leica microscope, and the percentage of SA-β-galactosidase+ cells was quantified by counting the number of stained cells by ImageJ.

Proximity ligation assays.
Cells were seeded on chamber slides, fixed with 4% formaldehyde at room temperature for 10 min, washed twice with PBS, and permeabilized with 0.5% Triton X-100/PBS solution (blocking solution) at room temperature for 30 min. Primary antibodies against mTOR, TGM2 (Proteintech, 68006-1-Ig), Rheb, or FLAG (Sigma-Aldrich, F1804) were incubated in blocking solution at 4°C overnight. Assays were then performed with the Duolink In Situ Red Starter Kit Mouse/Rabbit (Duolink, Sigma-Aldrich) according to the manufacturer's instructions using anti-mouse MINUS and anti-rabbit PLUS proximity ligation assay probes. Images were acquired by a Zeiss Axio Imager M2 microscope and analyzed for cytoplasmic fluorescence per cell with HALO software.

Site-directed mutagenesis.
The human *TPH1* (A300T) and *TGM2* (W241A) mutant lentiviral expression constructs were generated using a QuickChange II Site-Directed Mutagenesis Kit (Agilent Technologies) following the manufacturer's instructions, with the respective WT constructs as templates. The primer sequences for *TPH1* and *TGM2* mutagenesis are 5′-TTAAGACCTGGGGAACCATATTCCAAGAGCTCAAC-3′ and 5′-GTGCTGCTGGGACGCGCGGACAACAACTACGG-3′, respectively (mutated nucleotides underlined). The mTOR (Q2453A) mutant lentiviral expression construct was generated by VectorBuilder Inc. Mutated nucleotides were verified by DNA sequencing prior to experimental use.

Co-immunoprecipitation assays.
Co-IP assays were performed using the Pierce Co-Immunoprecipitation Kit (Thermo Fisher Scientific) following the manufacturer's protocol. Cells after different treatments or co-transfections were lysed in ice-cold IP lysis/wash buffer, pre-cleared with control agarose resin at 4°C for 30 min, and incubated at 4°C overnight with serotonin (Novus Biologicals, NB120-16007), FLAG, Myc (Proteintech, 60003-2-Ig), mTOR, or control IgG antibodies pre-immobilized on AminoLink Plus coupling resin. Immunoprecipitates from 500 µg of total protein lysates were washed six times with lysis buffer, eluted with elution buffer, and analyzed by immunoblotting with antibodies against mTOR, FLAG, Myc, or Rheb, with 10% of total protein lysates used as an input control.

Conformational analysis of mTOR (Q2453A)
Structural analysis of the mTOR Q2453A mutation was conducted using the PyMOL Molecular Graphics System (version 2.0, Schrödinger, LLC). The WT mTOR structure was retrieved from the RCSB Protein Data Bank (PDB ID: 6BCU). The Q2453A mutant model was generated using the Mutagenesis Wizard in PyMOL. Hydrogen-bond interactions and local residue contacts surrounding position 2453 were evaluated by comparative structural visualization of the WT and mutant models.
