# Supplementary material for: Targeting tryptophan hydroxylase 1 restricts growth and suppresses plasticity in neuroendocrine prostate cancer
Source: J Clin Invest. 2026 Sep 17;136(22):e200391. doi: 10.1172/JCI200391 (PMC13767635; doi:10.1172/JCI200391)

Full unedited blots for Figure 1E

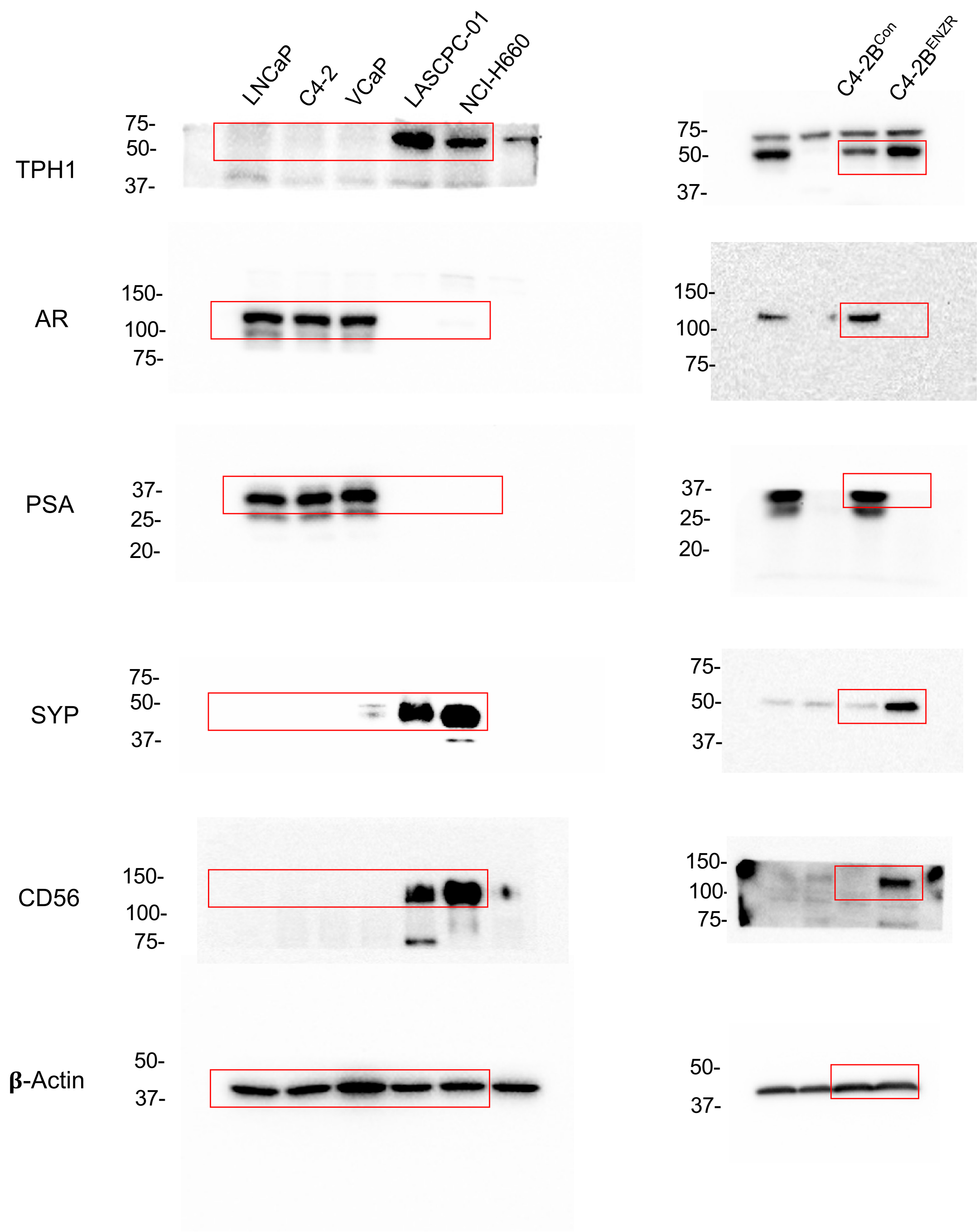

Full unedited blots for Figure 2A

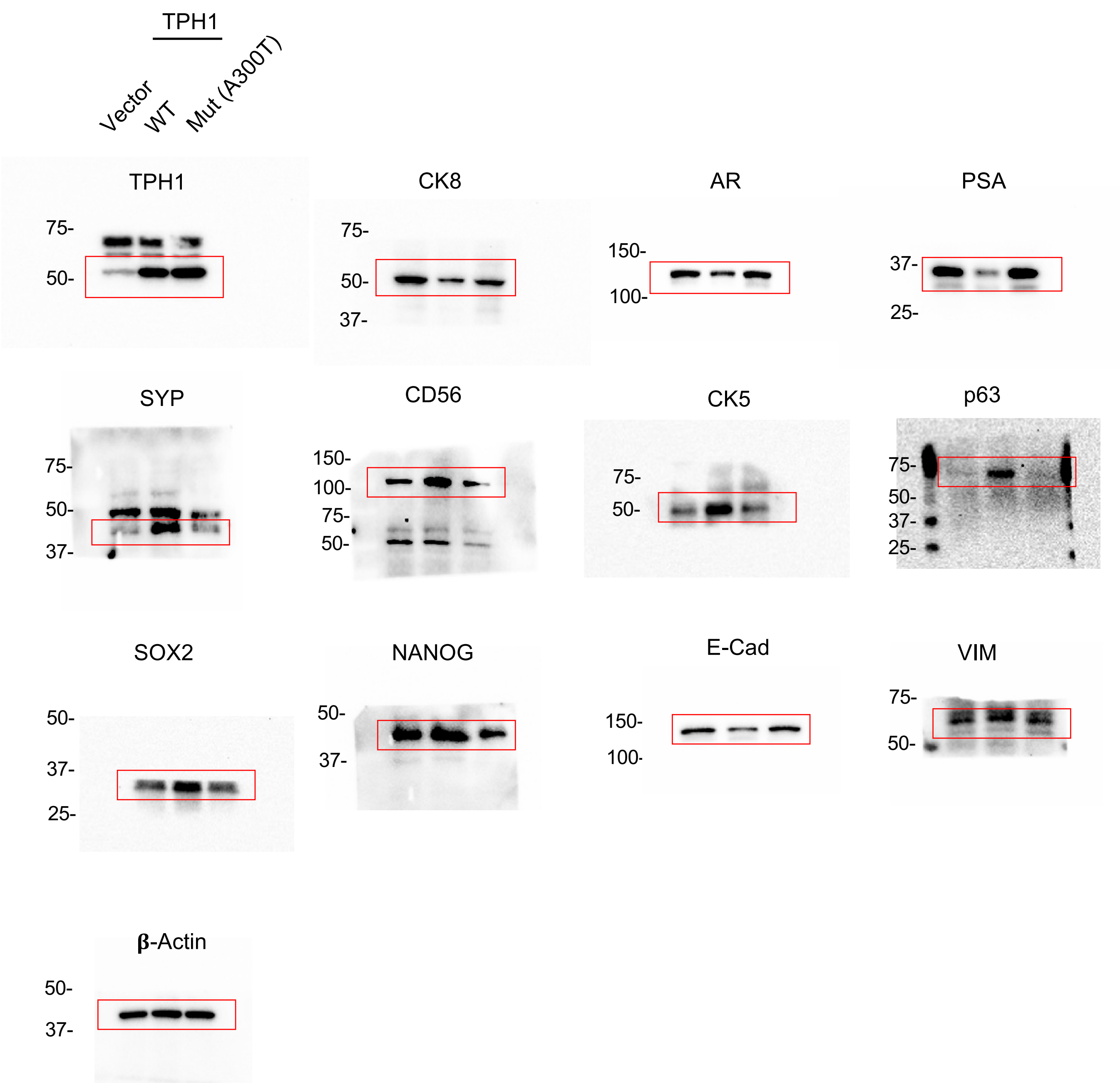

Full unedited blots for Figure 3A

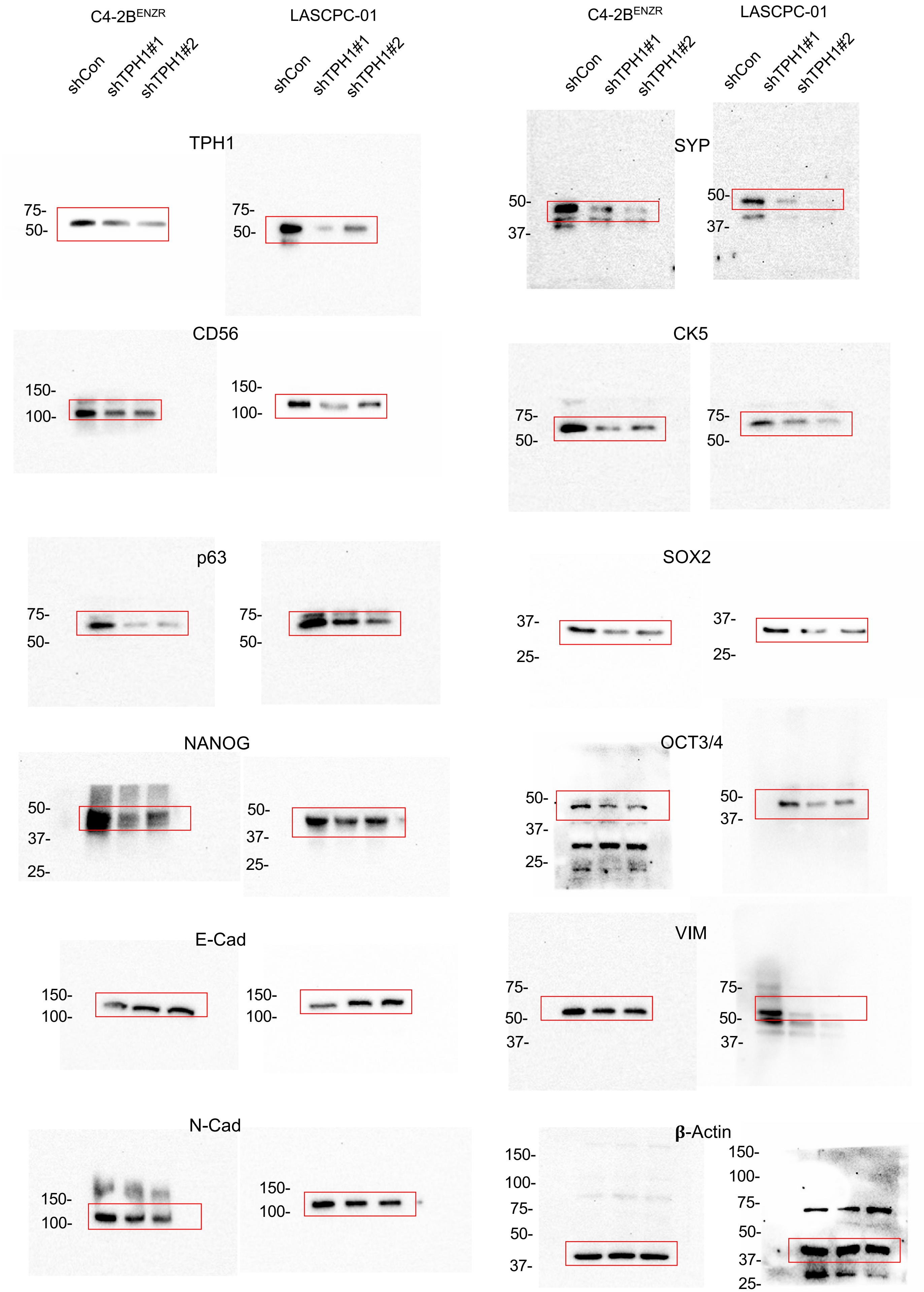

Full unedited blots for Figure 5B

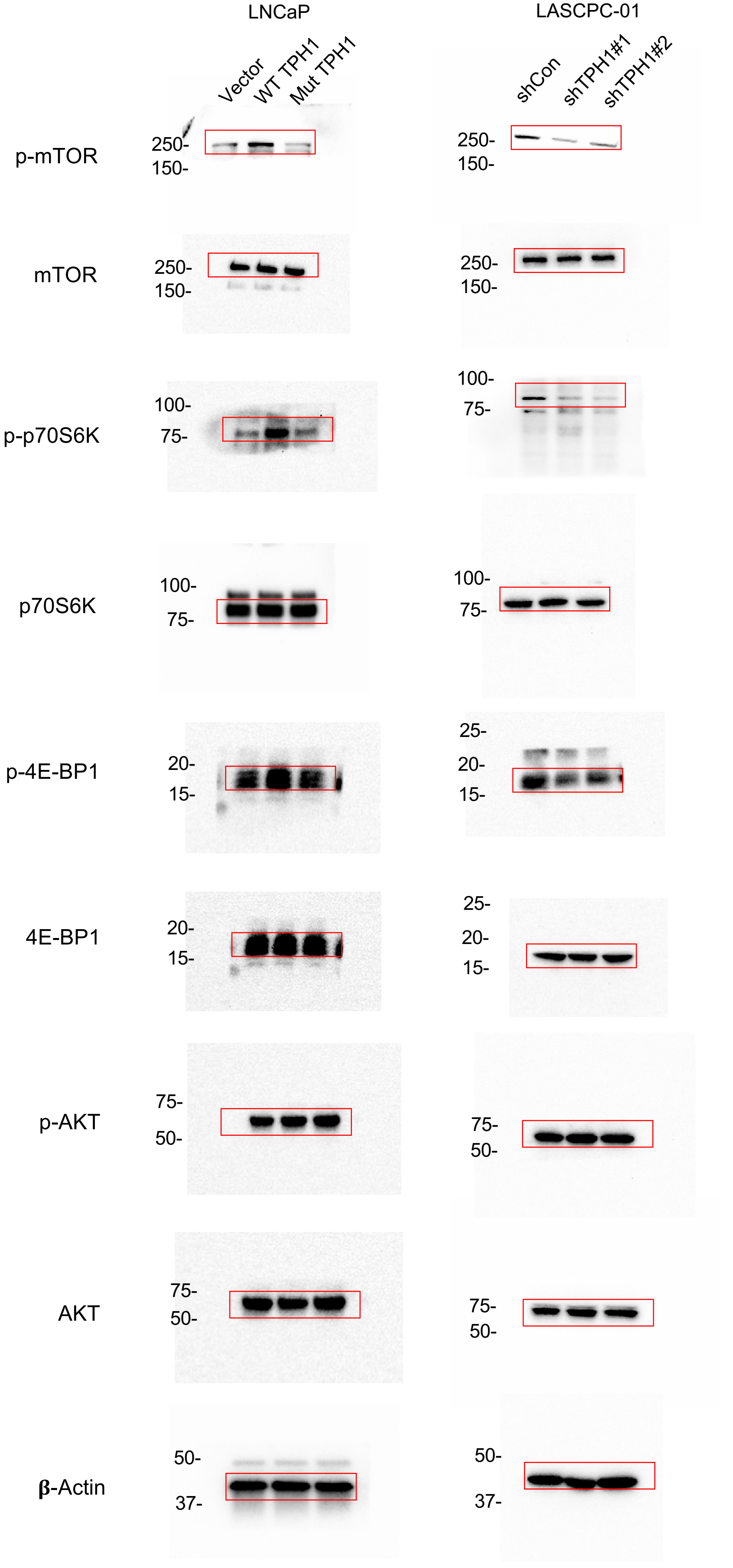

Full unedited blots for Figure 5D

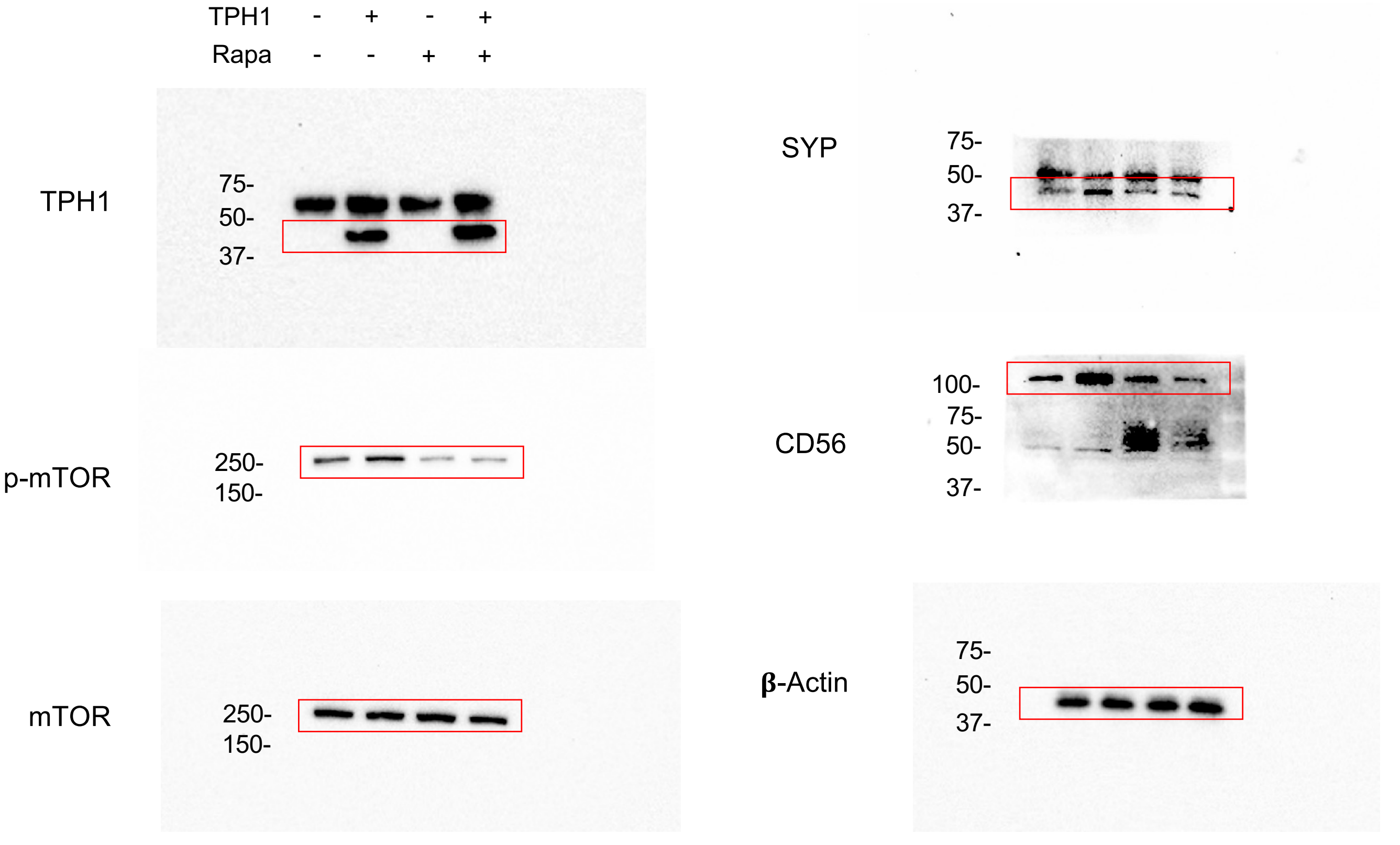

Full unedited blots for Figure 5F

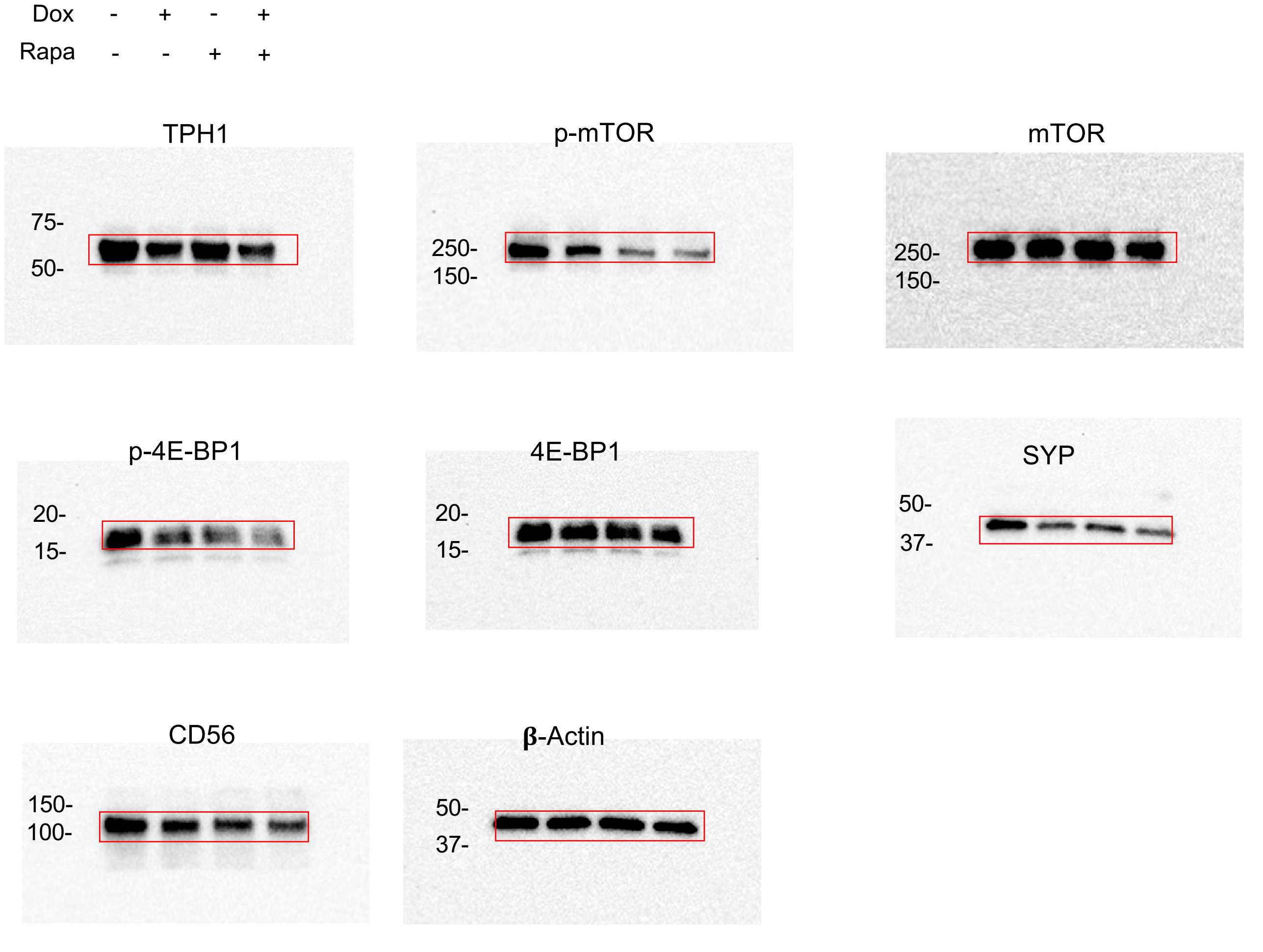

Full unedited blots for Figure 5H

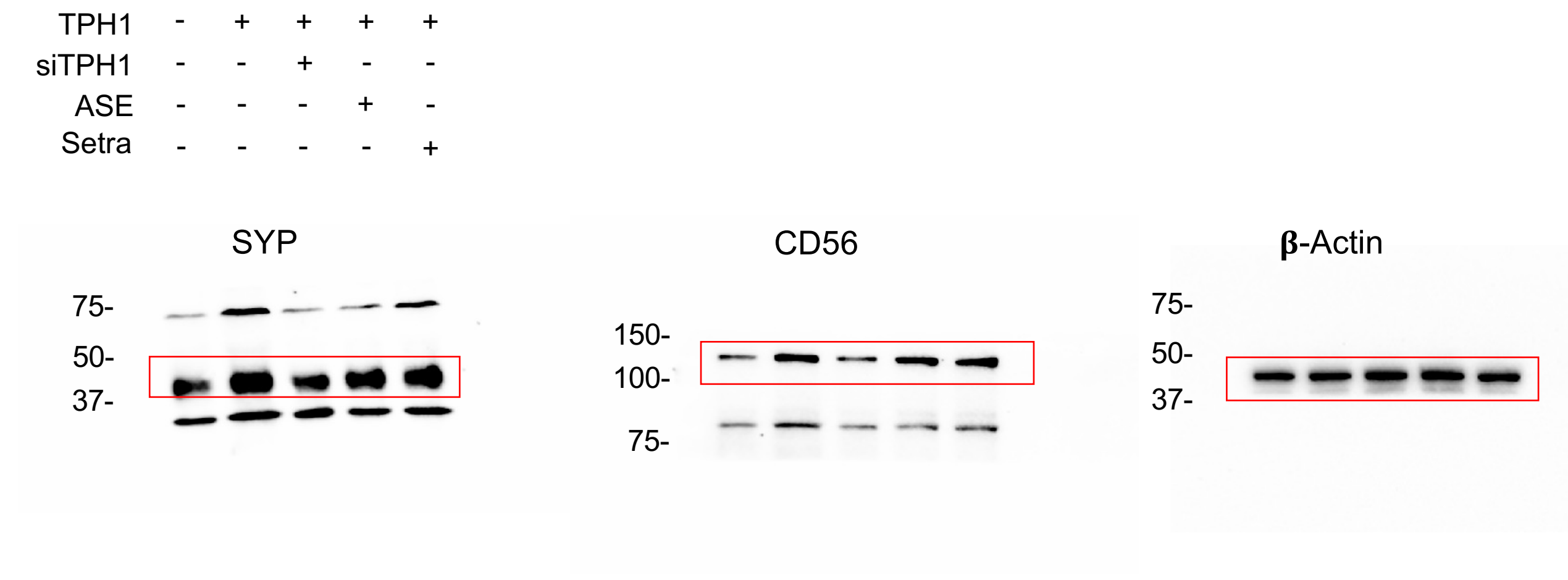

Full unedited blots for Figure 5J

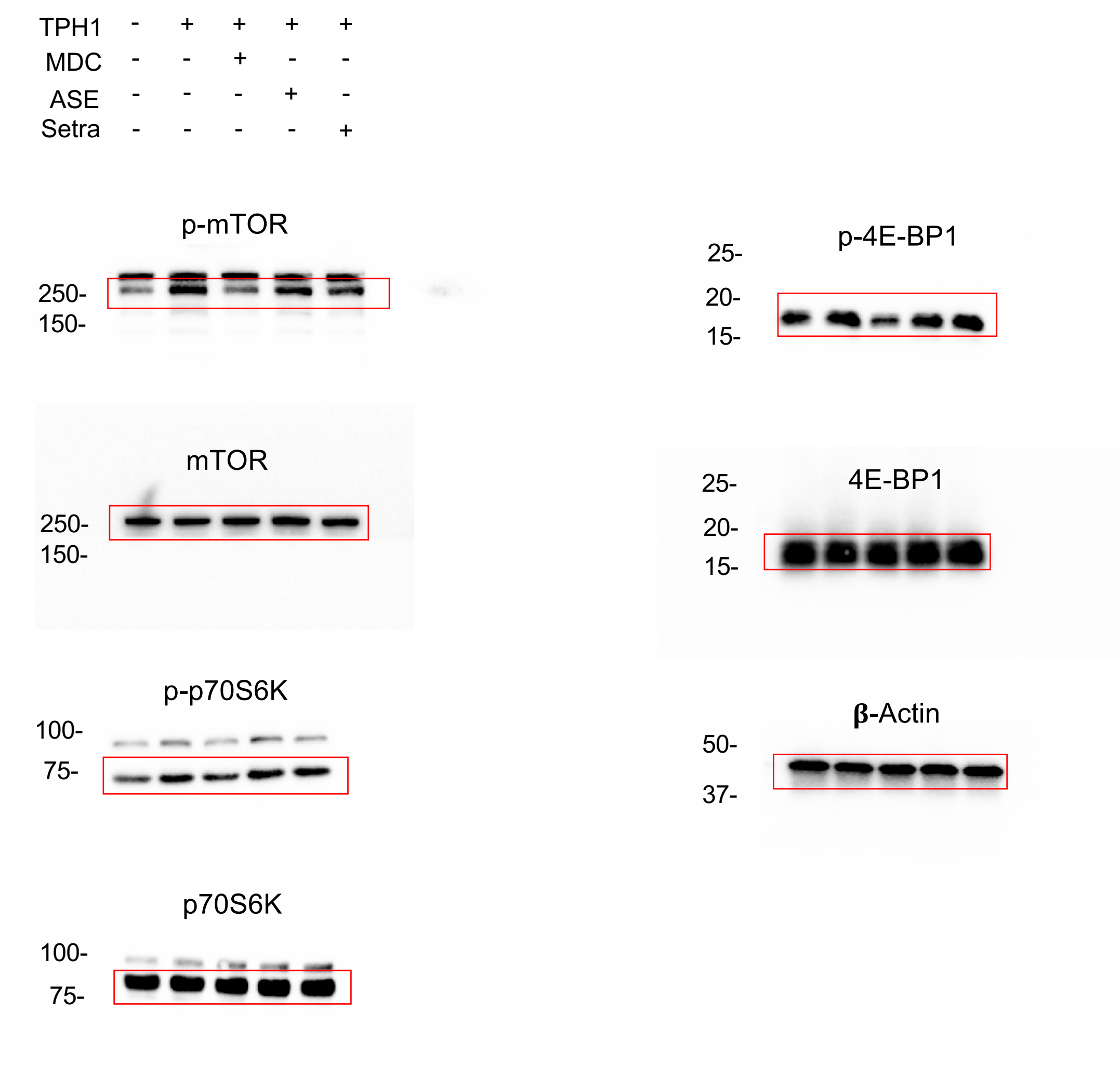

Full unedited blots for Figure 6A

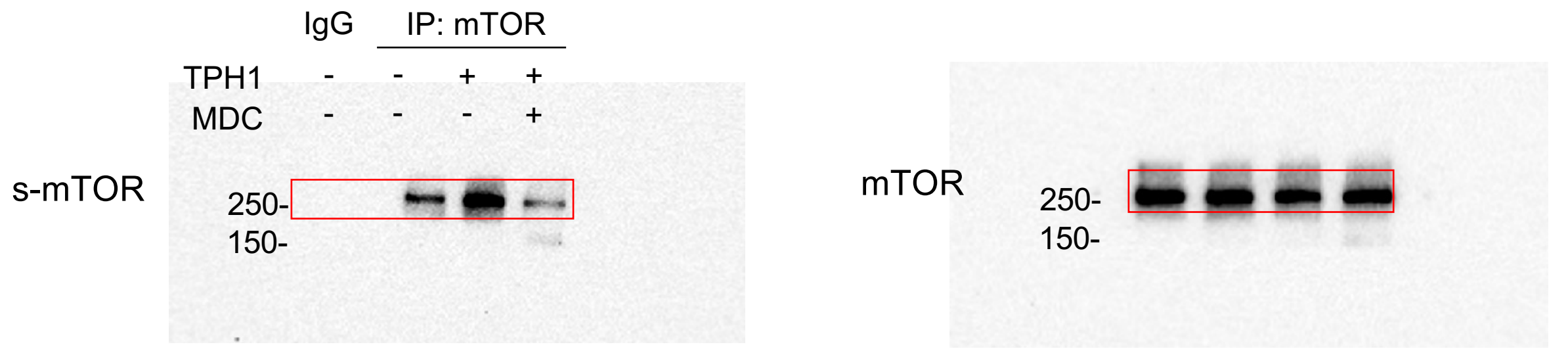

Full unedited blots for Figure 6D

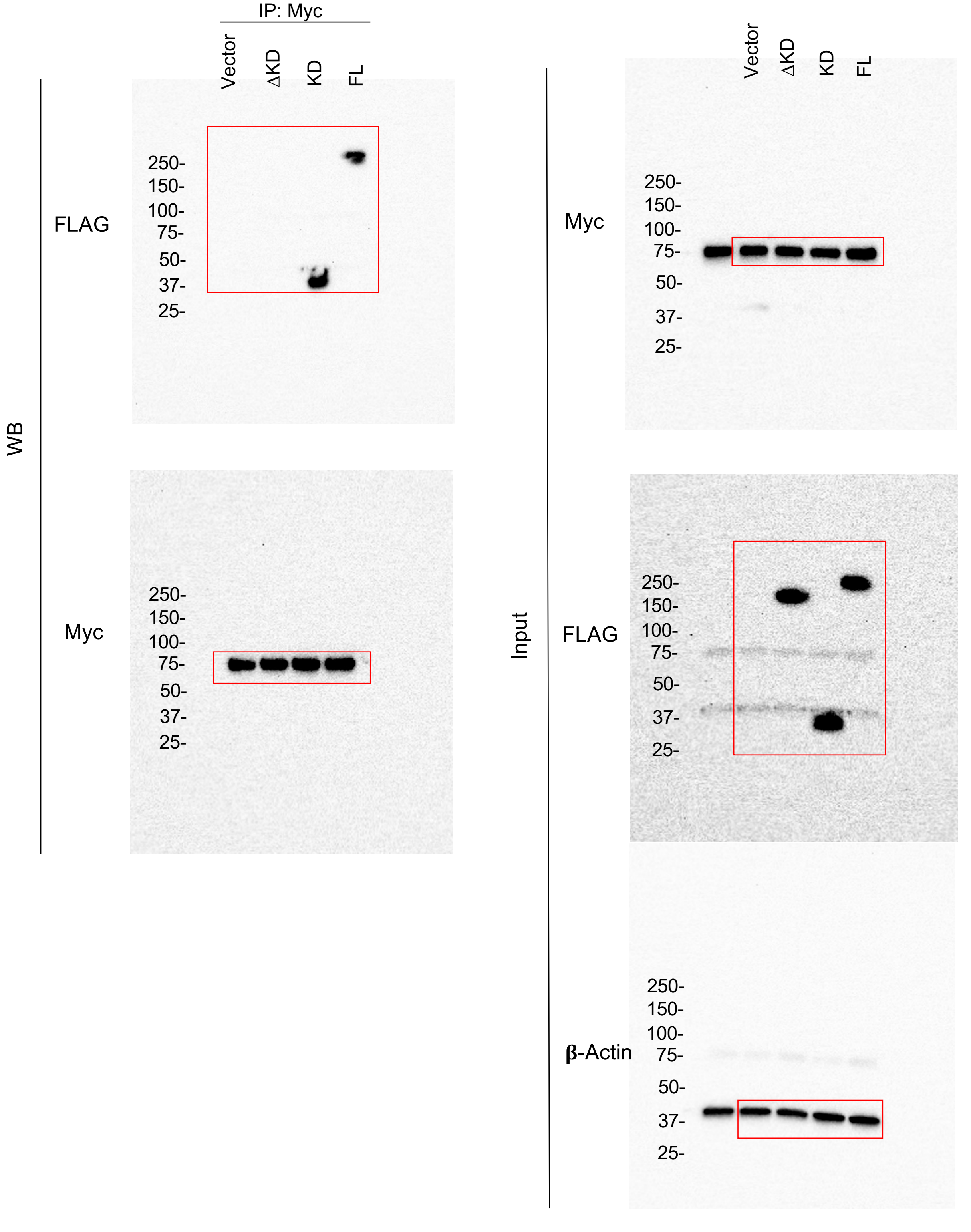

Full unedited blots for Figure 6F

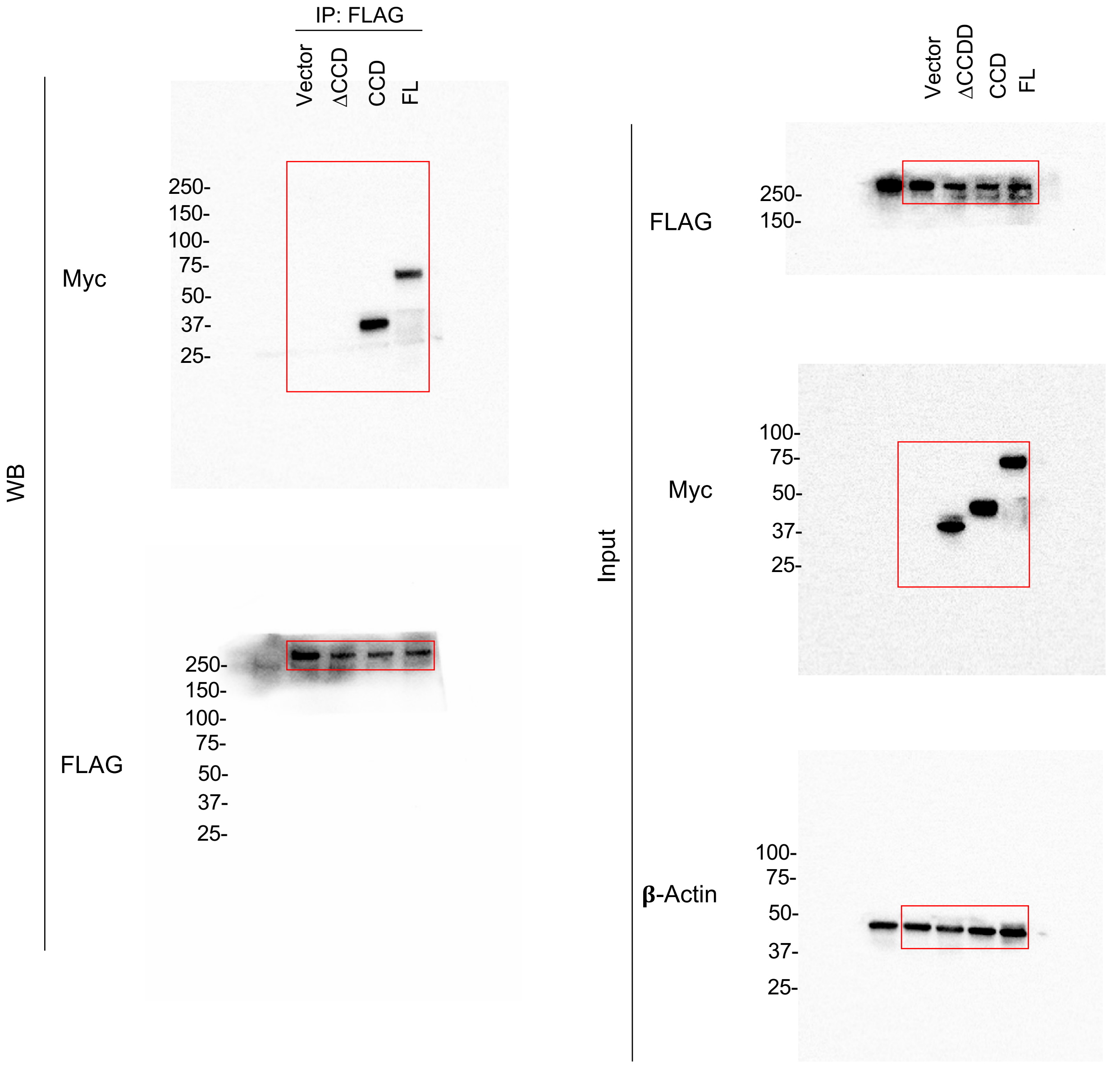

Full unedited blots for Figure 7B

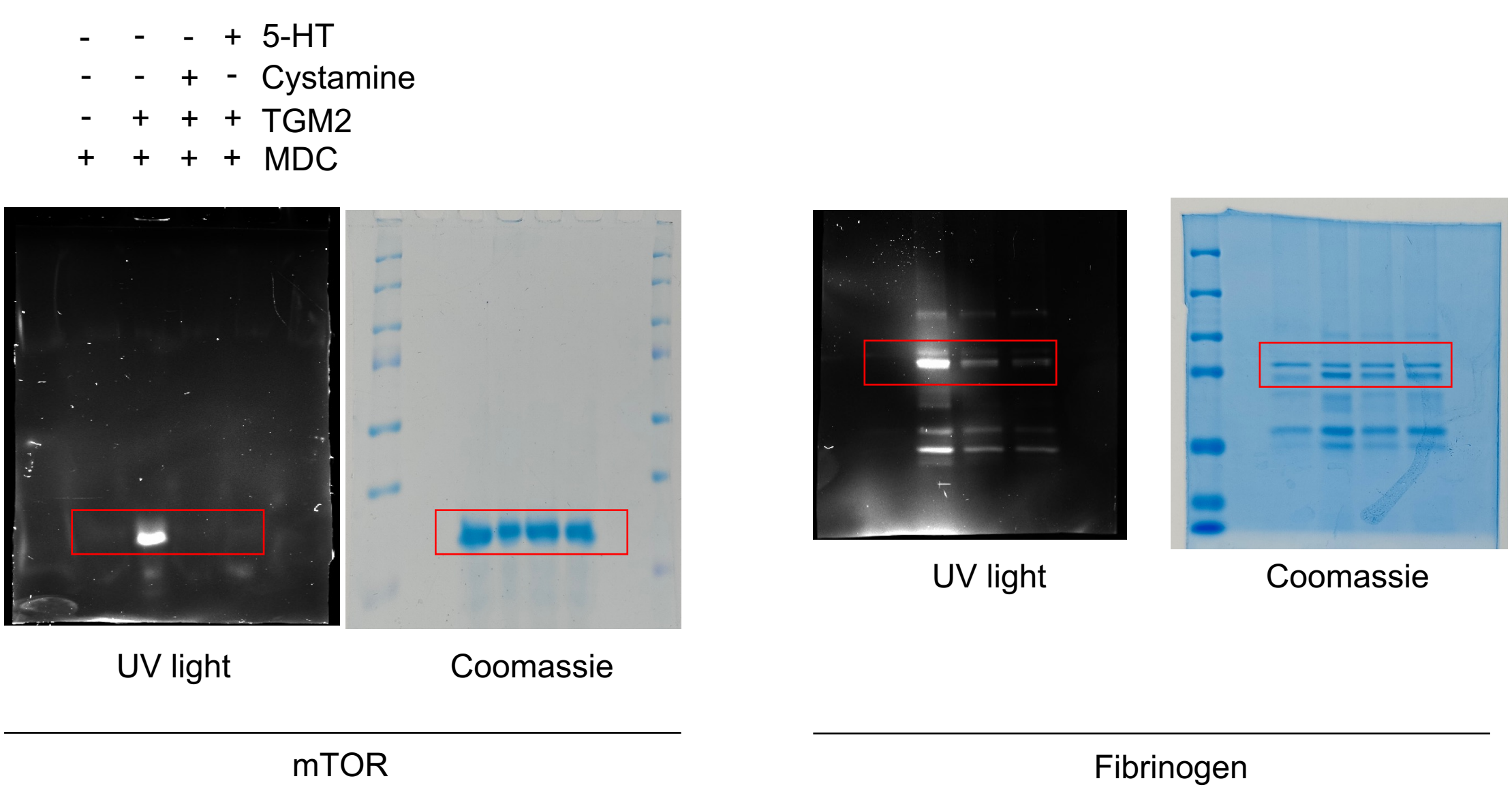

Full unedited blots for Figure 7D

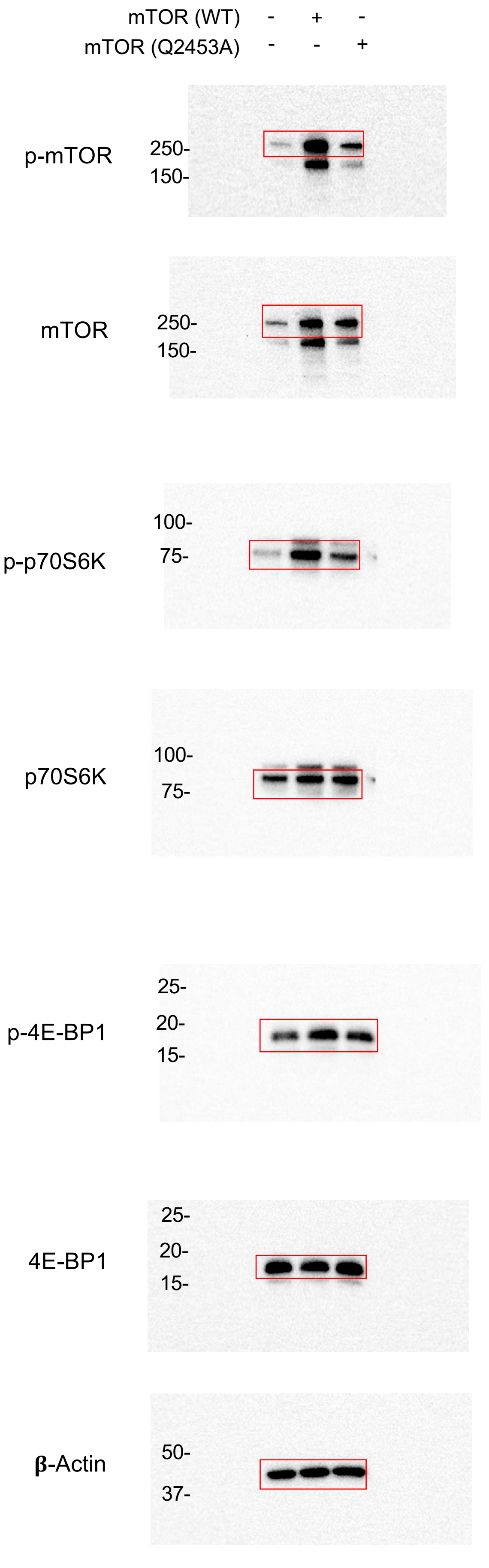

Full unedited blots for Figure 7F

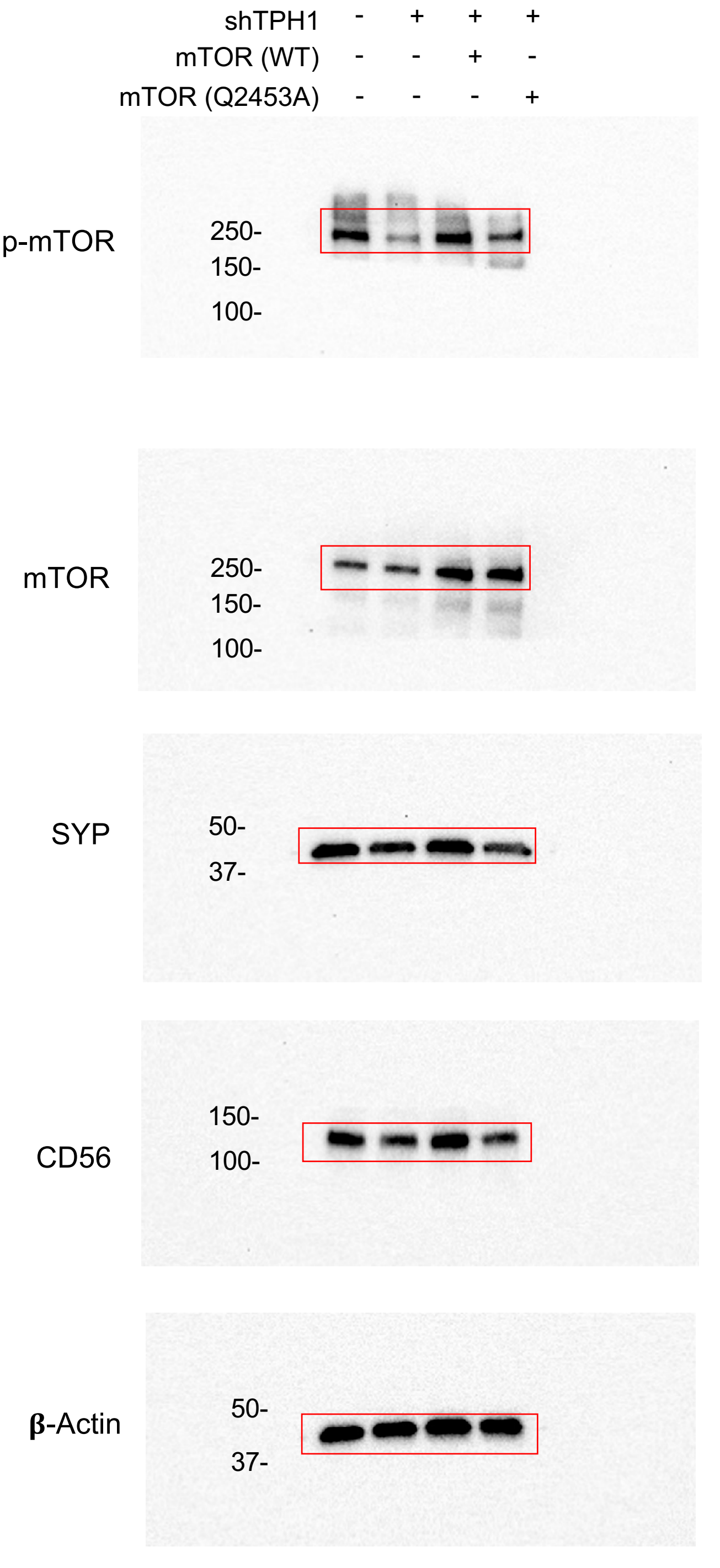

Full unedited blots for Figure 8C

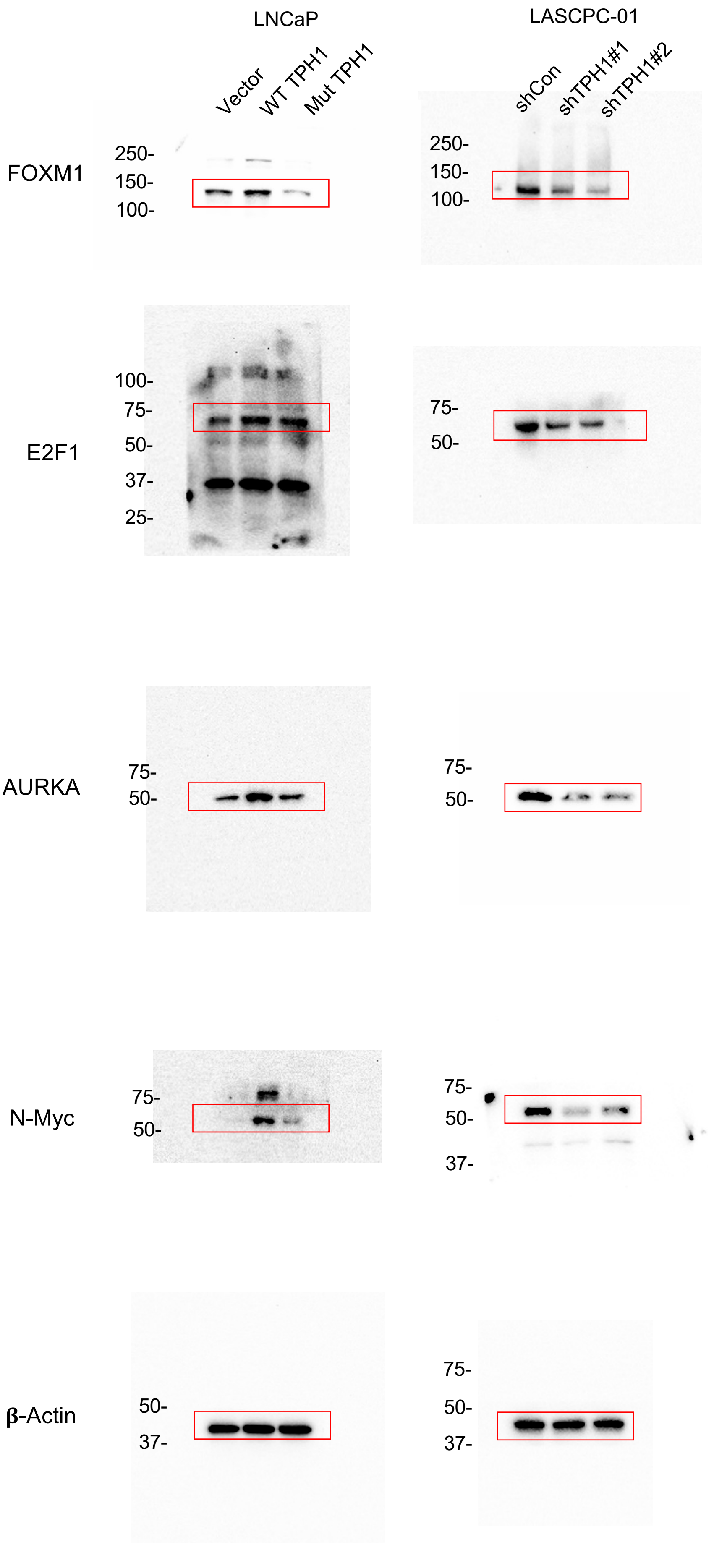

Full unedited blots for Figure 8F

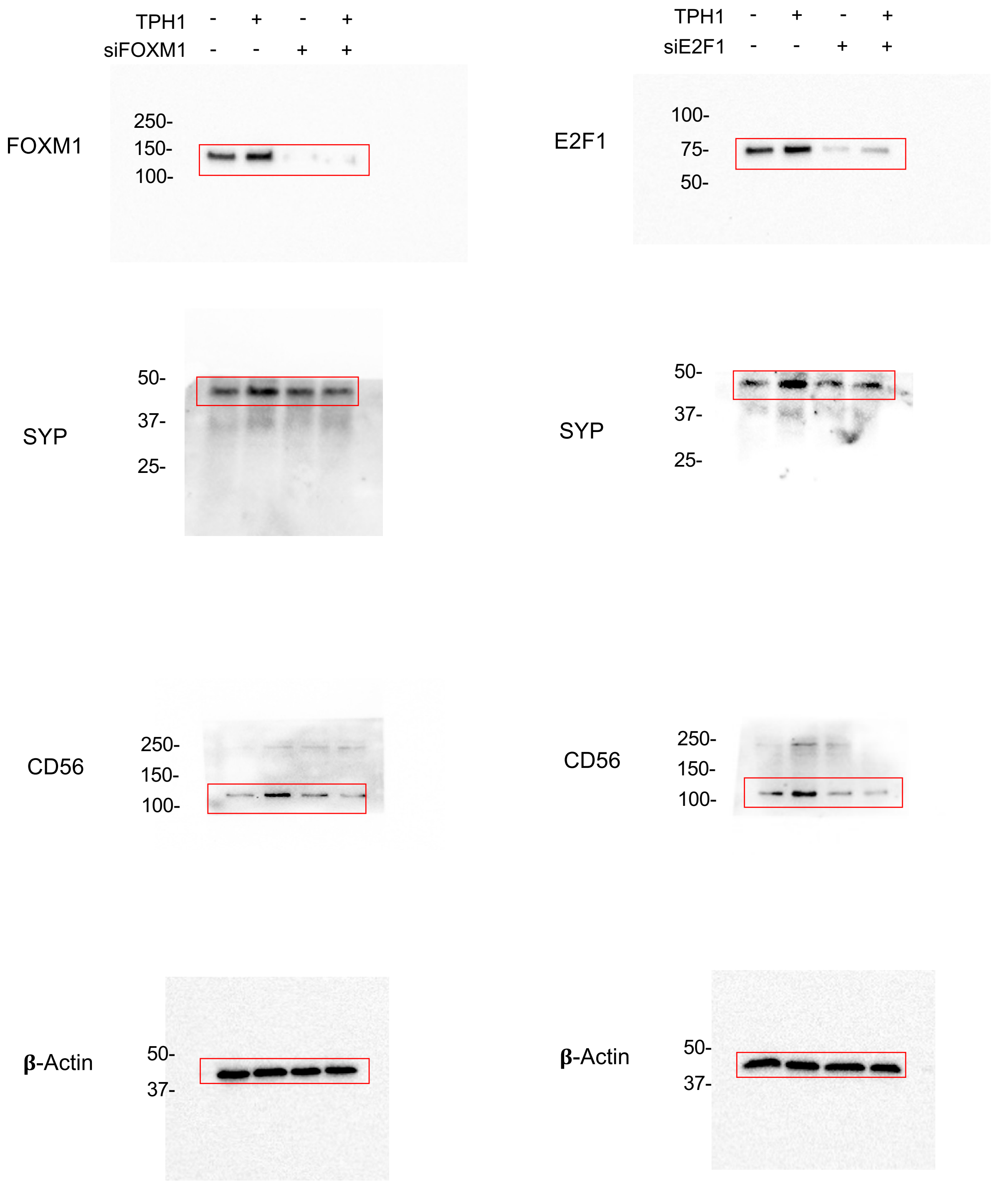

Full unedited blots for Figure 8H

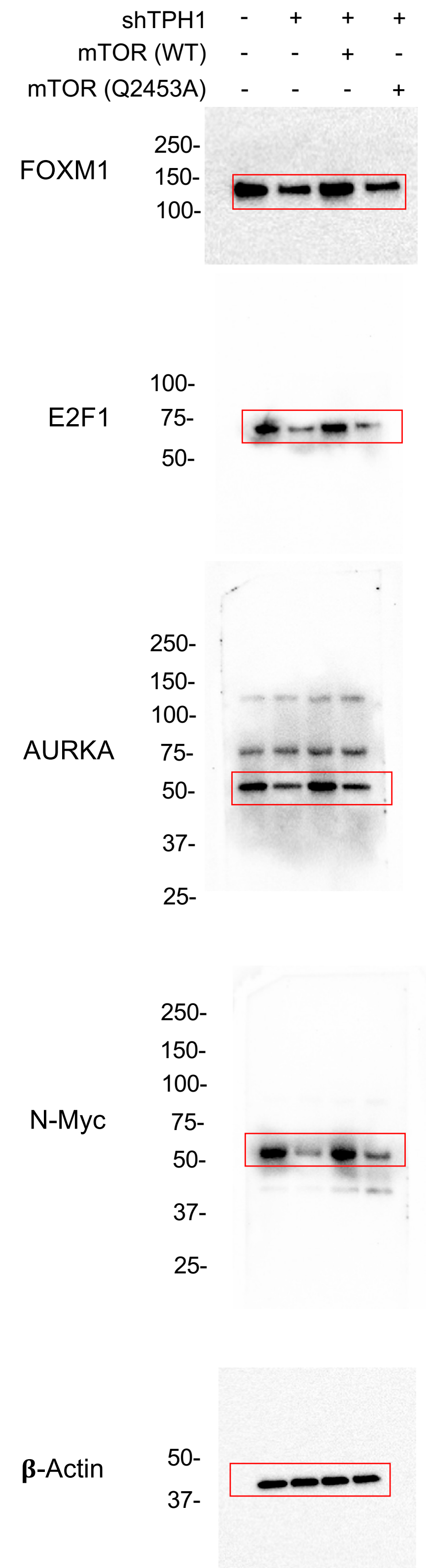

Full unedited blots for Figure 9D

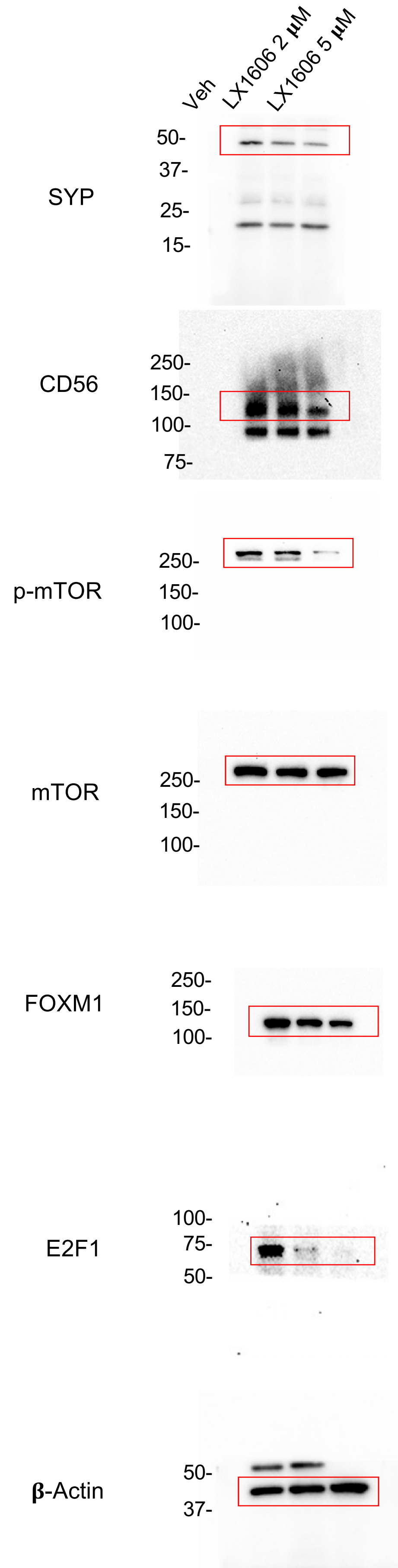

Full unedited blots for Supplemental Figure 1D

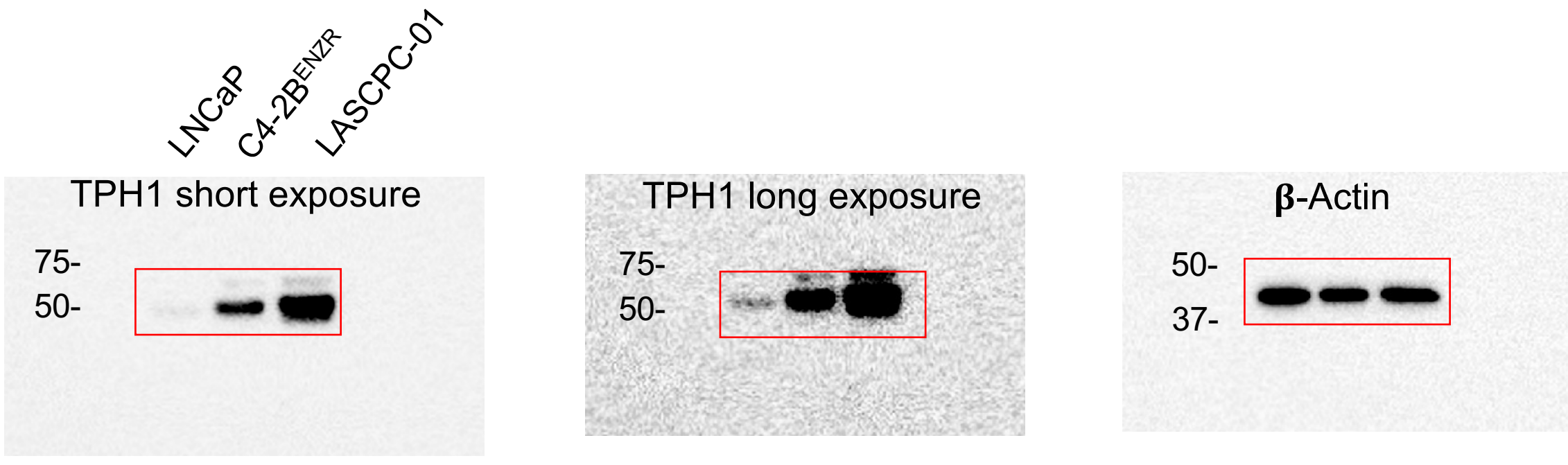

Full unedited blots for Supplemental Figure 1E

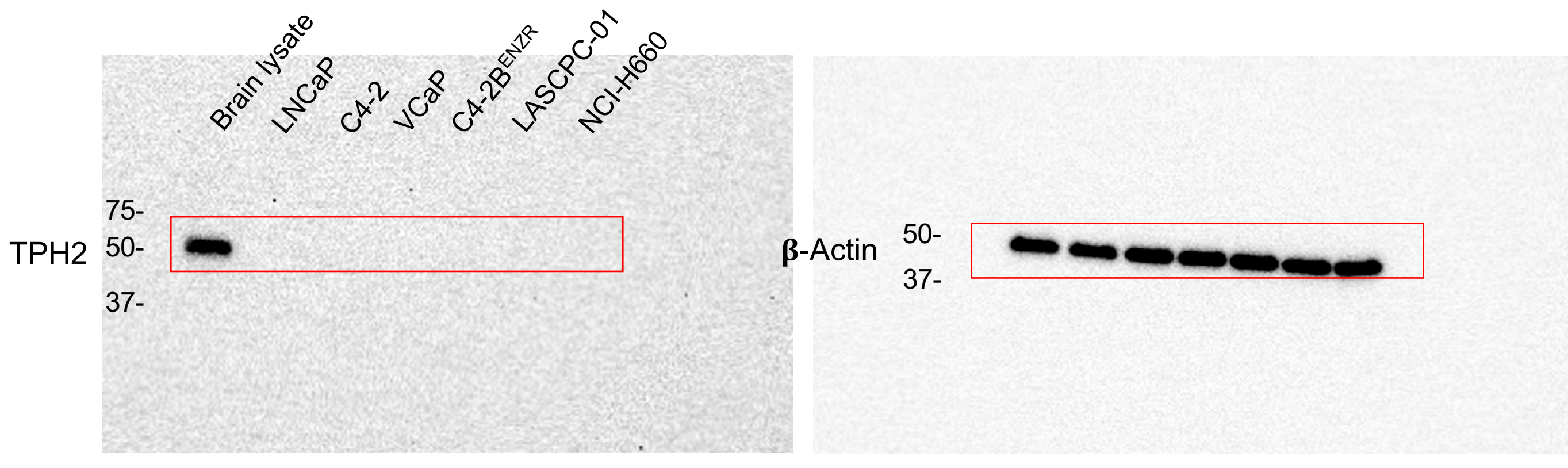

Full unedited blots for Supplemental Figure 1H

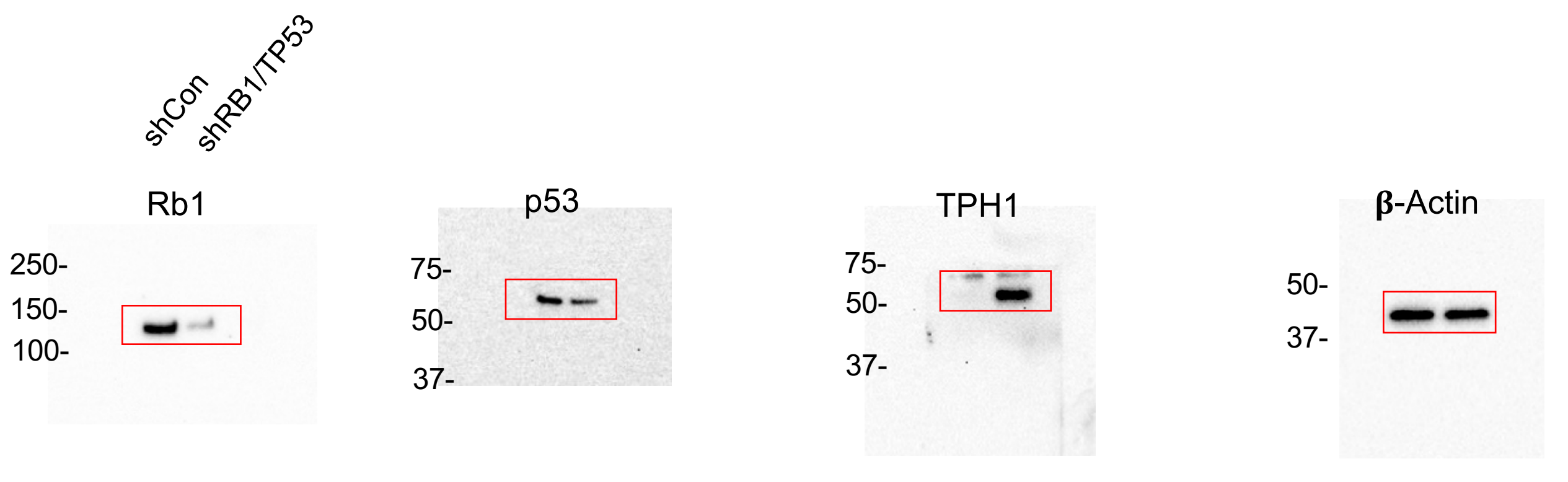

Full unedited blots for Supplemental Figure 2A

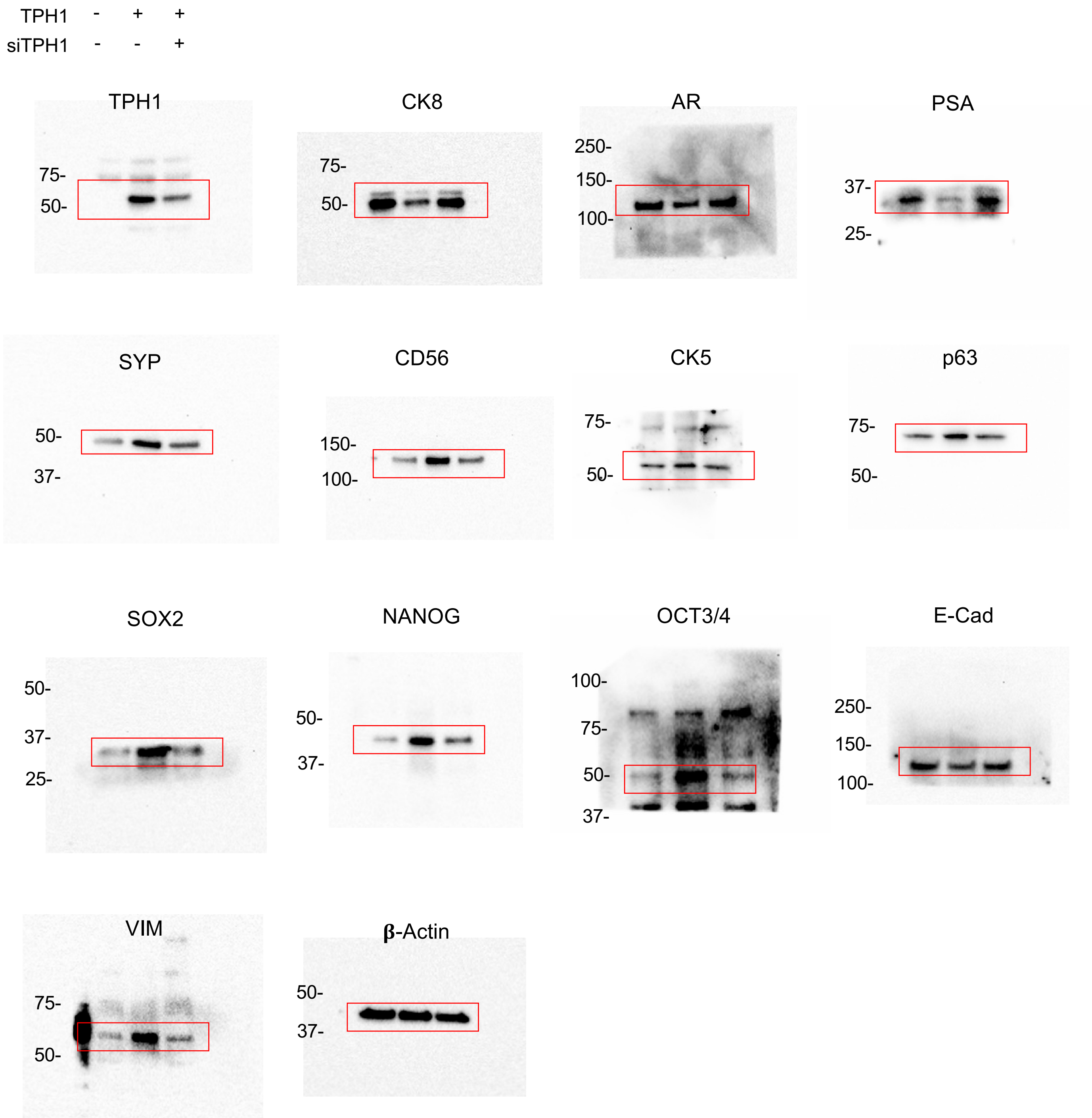

Full unedited blots for Supplemental Figure 2C

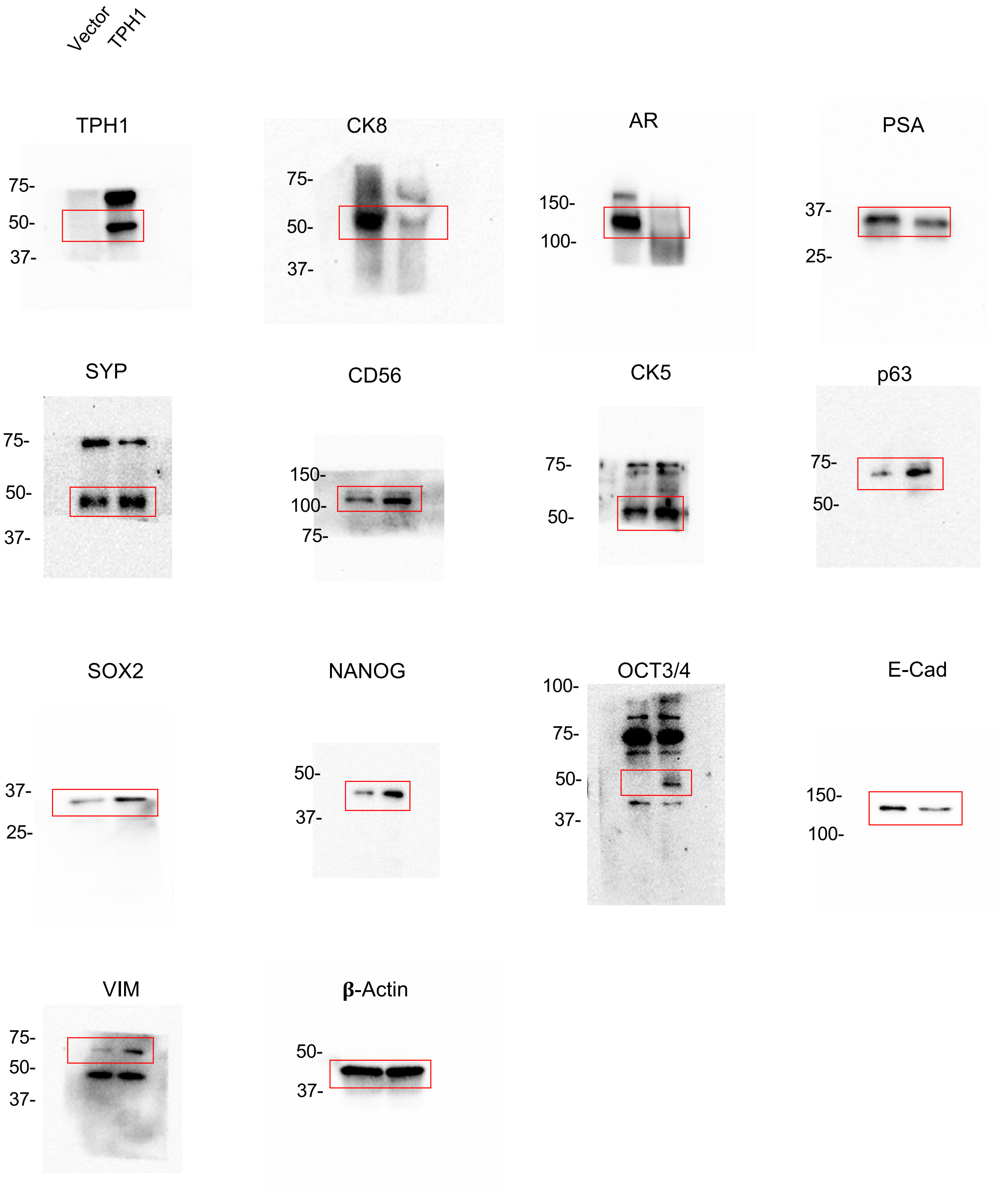

Full unedited blots for Supplemental Figure 4A

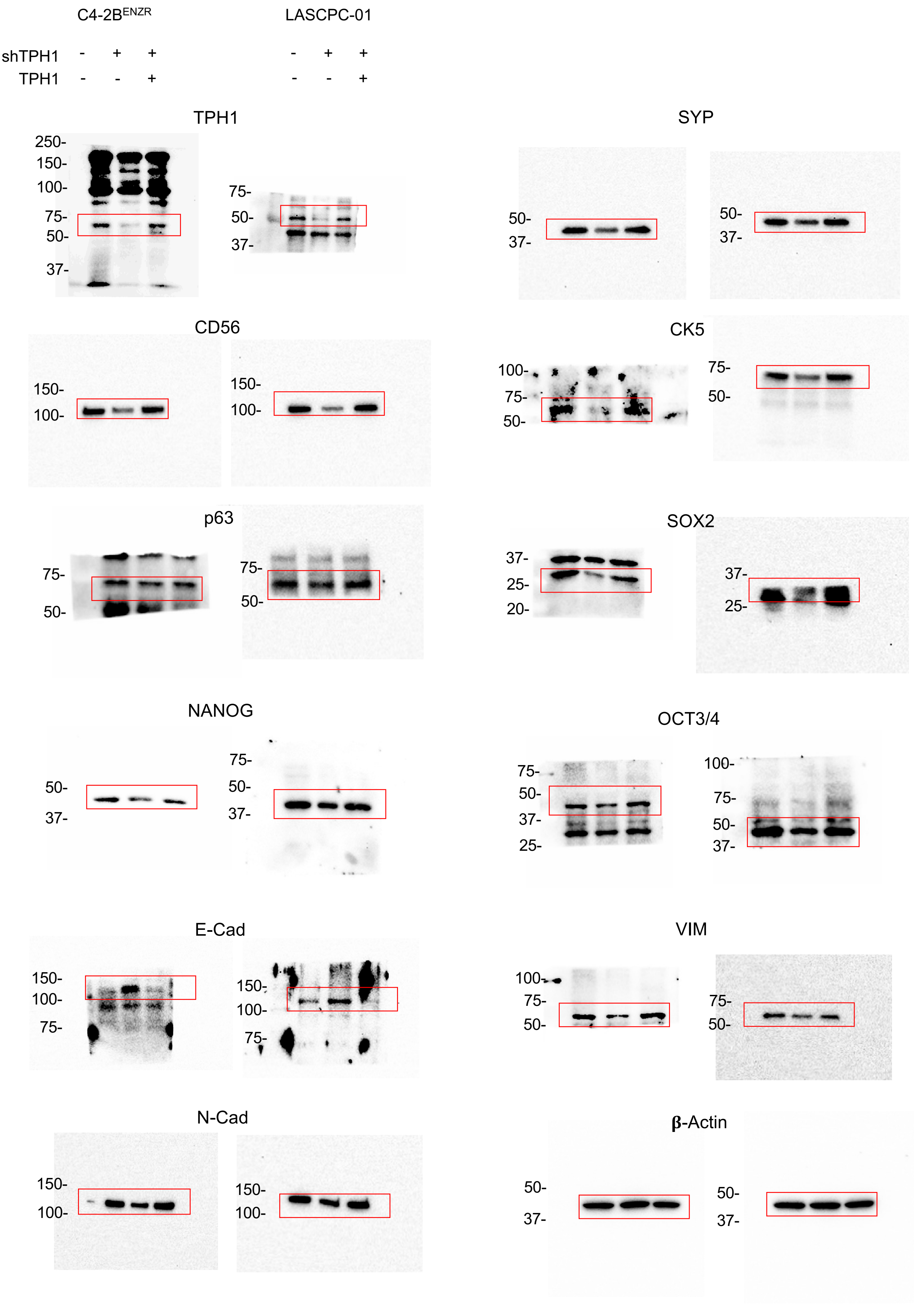

Full unedited blots for Supplemental Figure 4D

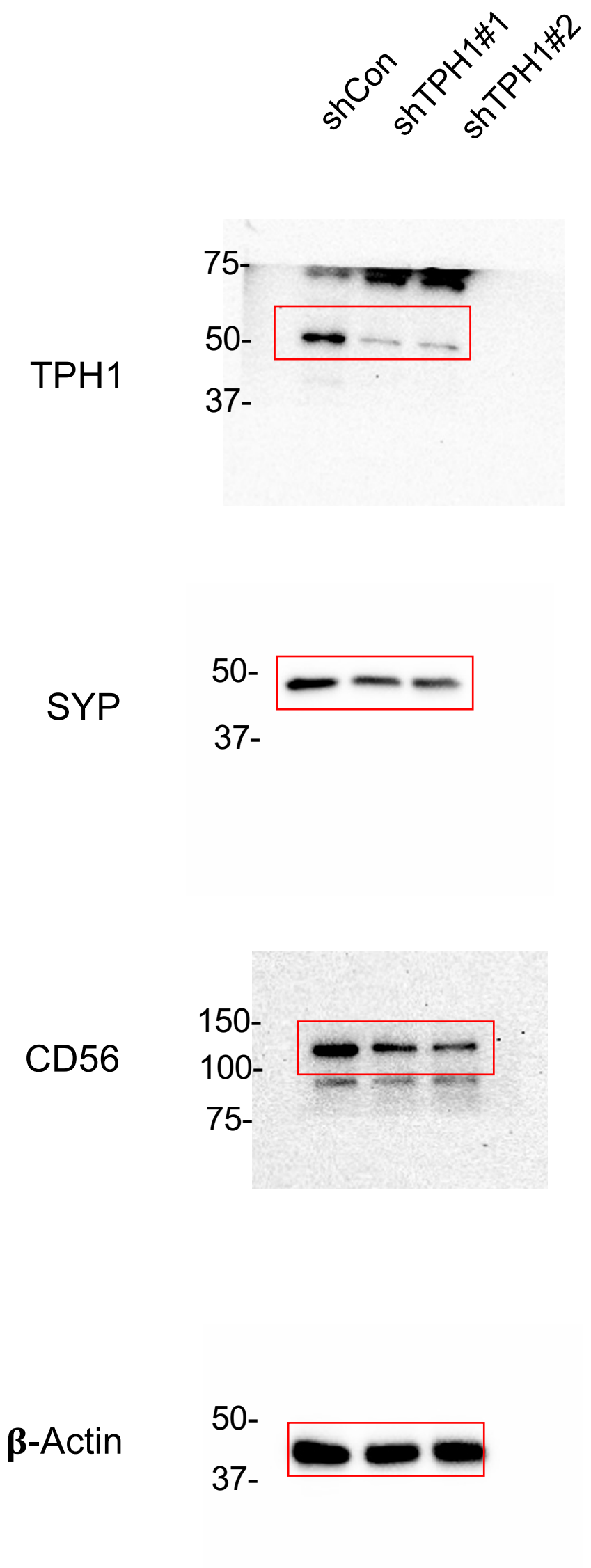

Full unedited blots for Supplemental Figure 4H

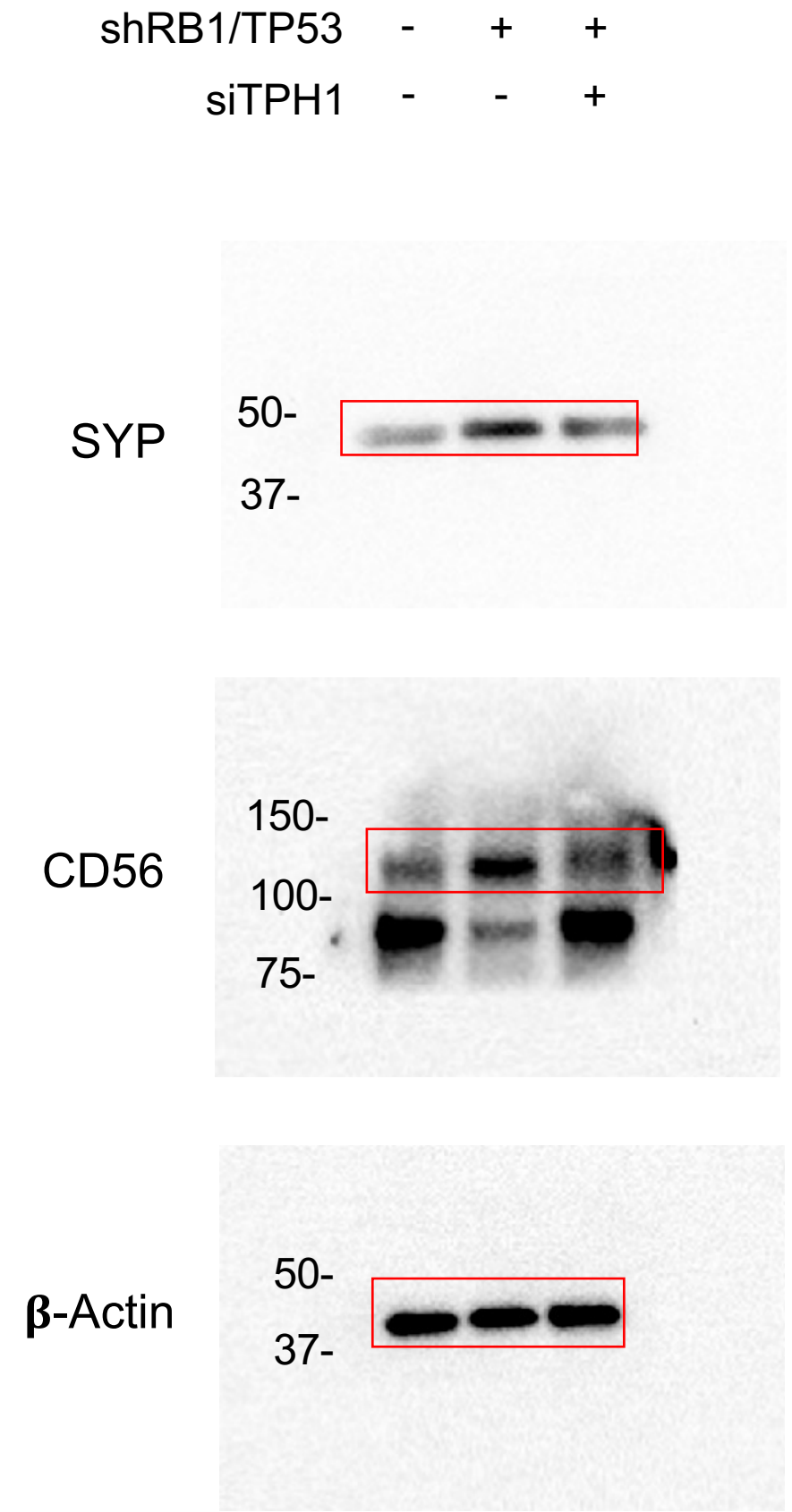

Full unedited blots for Supplemental Figure 6A

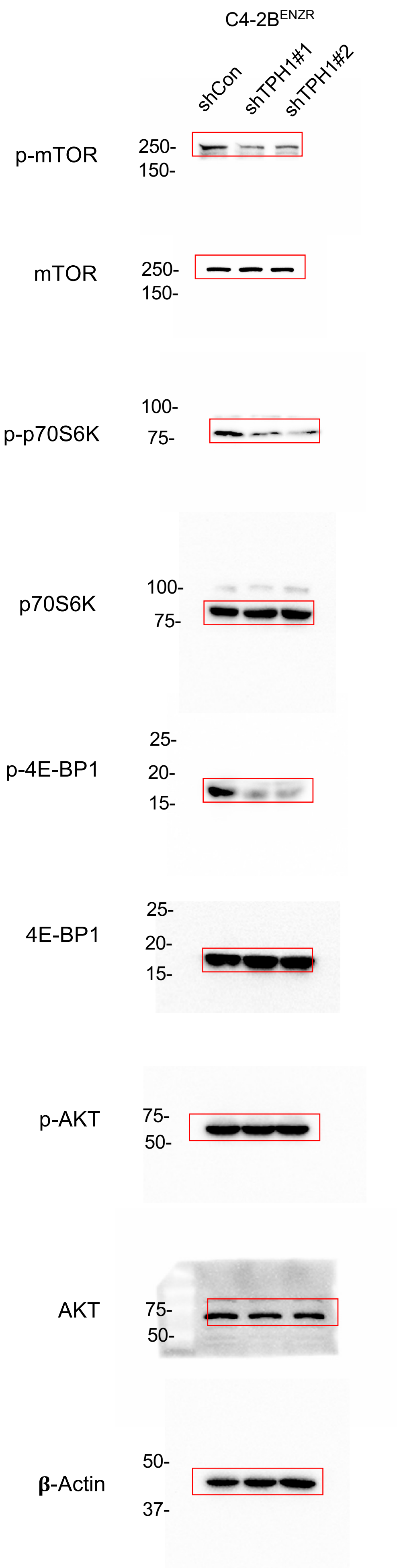

Full unedited blots for Supplemental Figure 6B

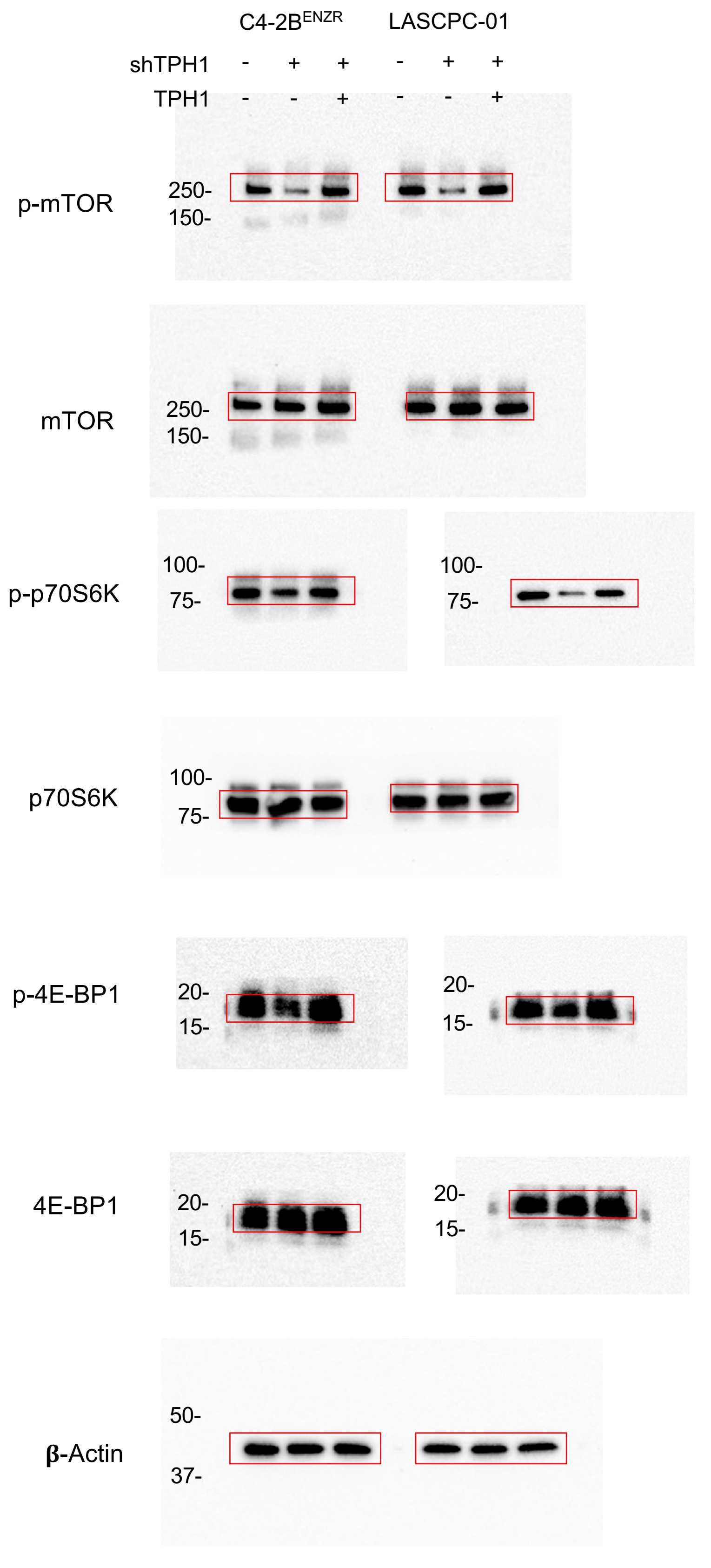

Full unedited blots for Supplemental Figure 6C

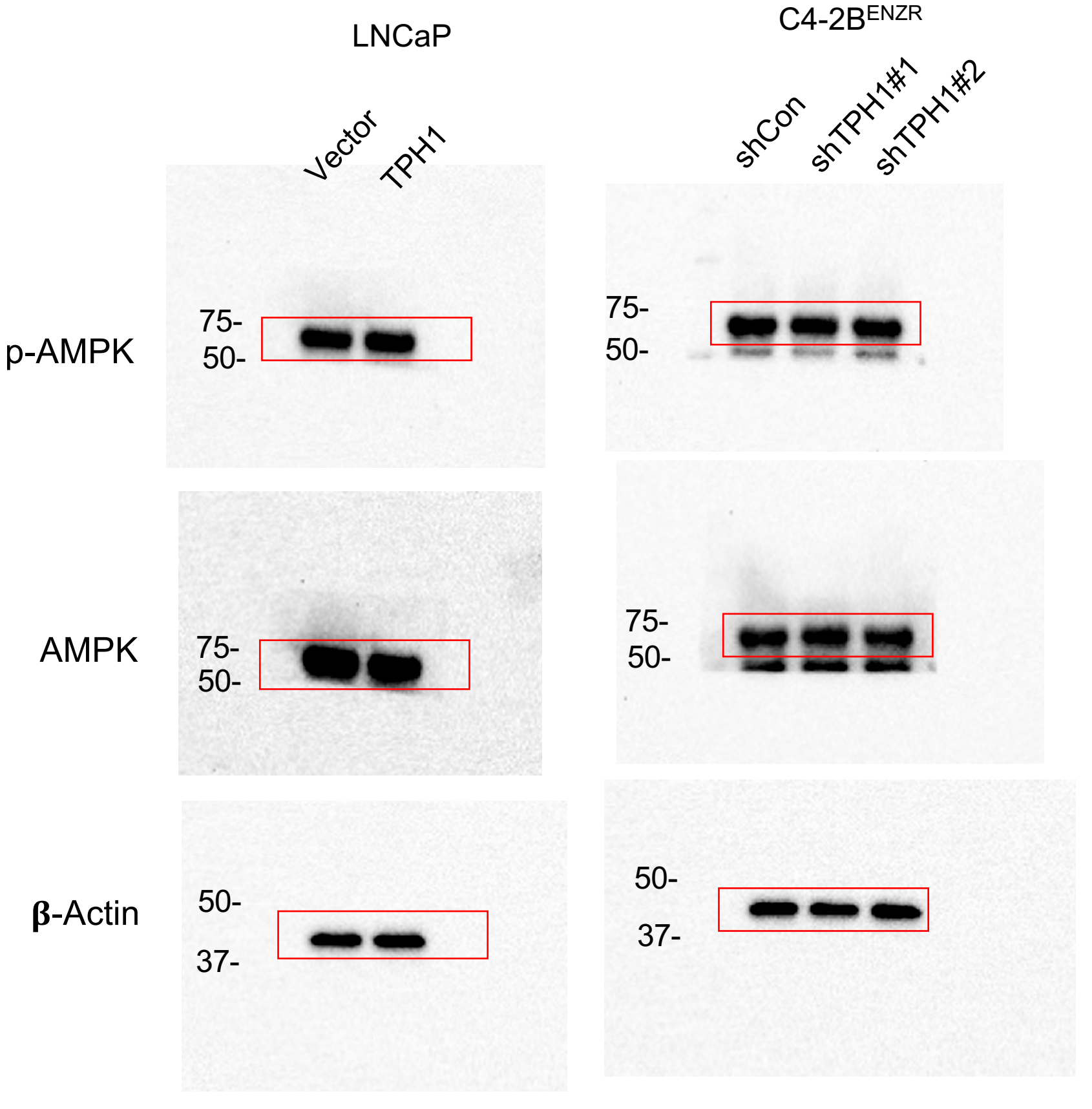

Full unedited blots for Supplemental Figure 6E

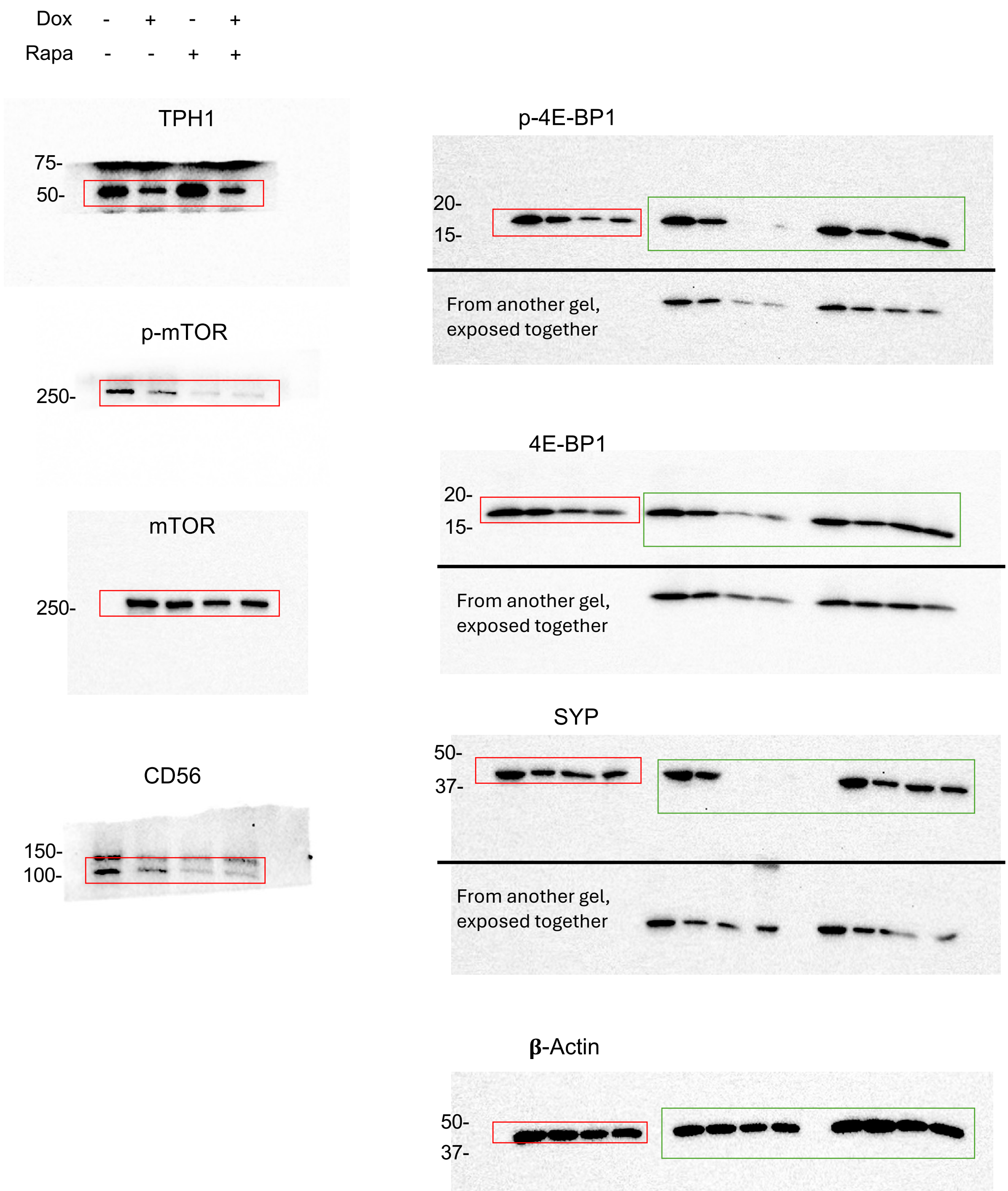

Annotation:
Red box: Results used in this experiment.
Green box: Samples run on the same gel but not related to this experiment.
Black line: Boundary between different gels.

Full unedited blots for Supplemental Figure 6F

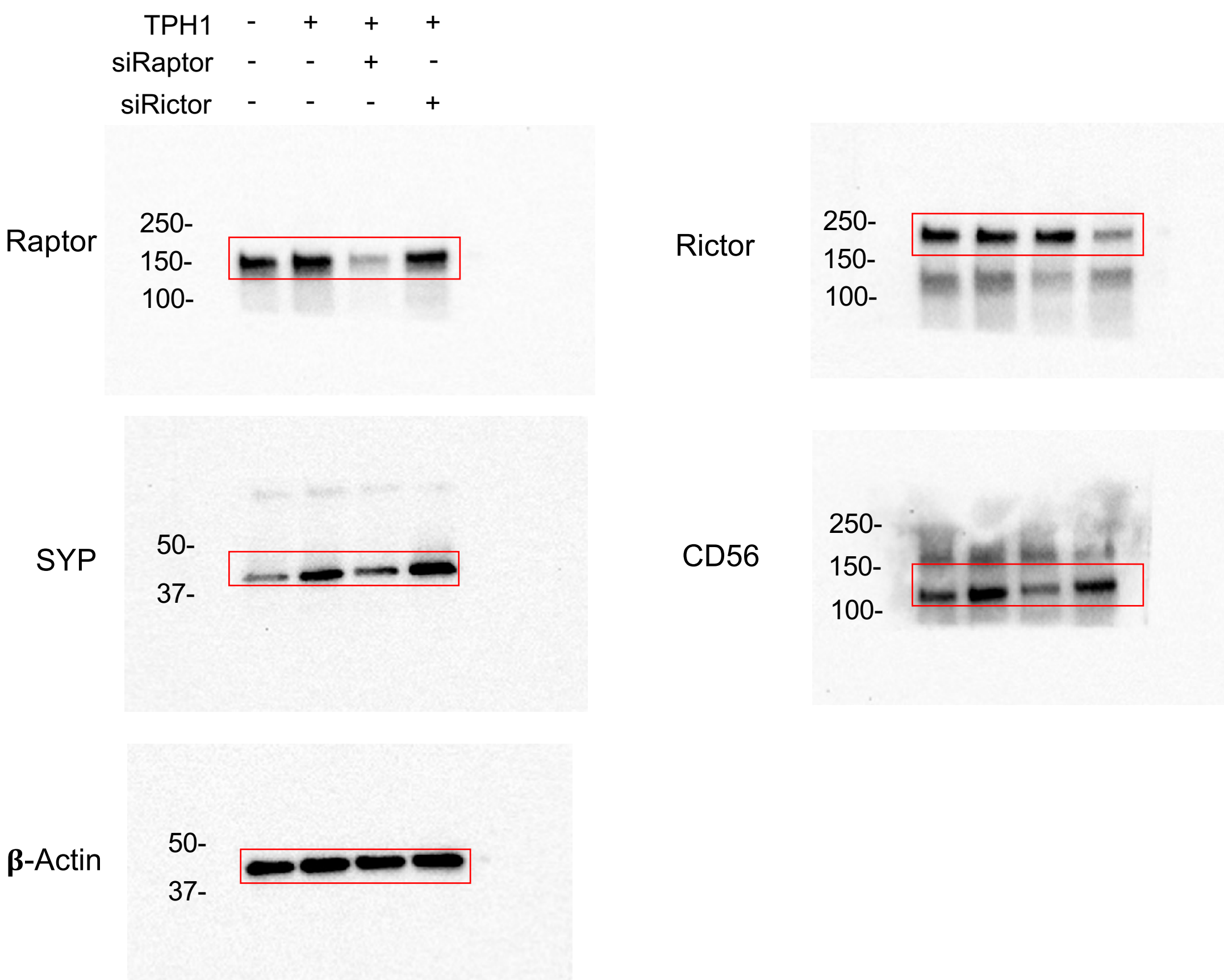

Full unedited blots for Supplemental Figure 7B

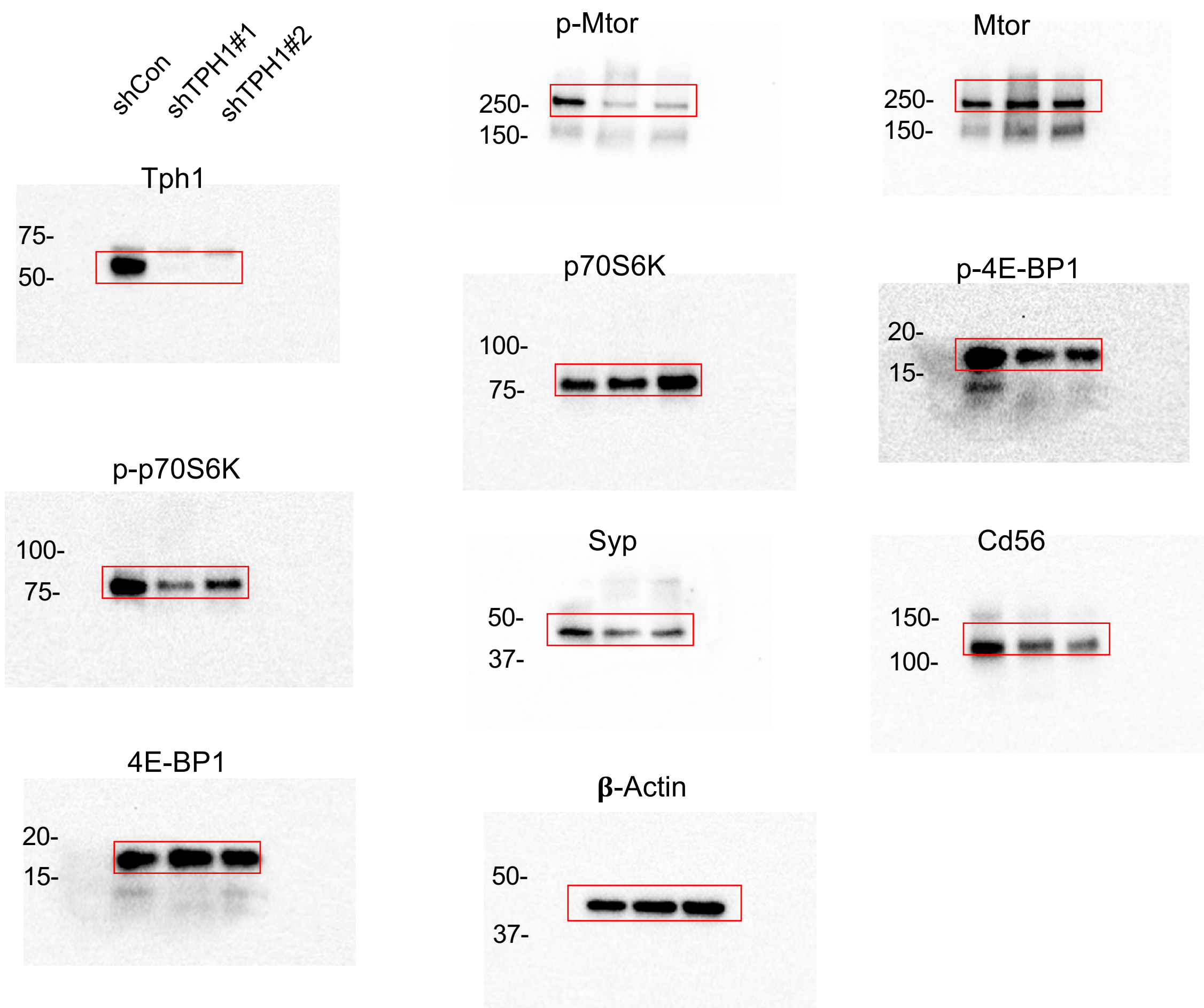

Full unedited blots for Supplemental Figure 8B

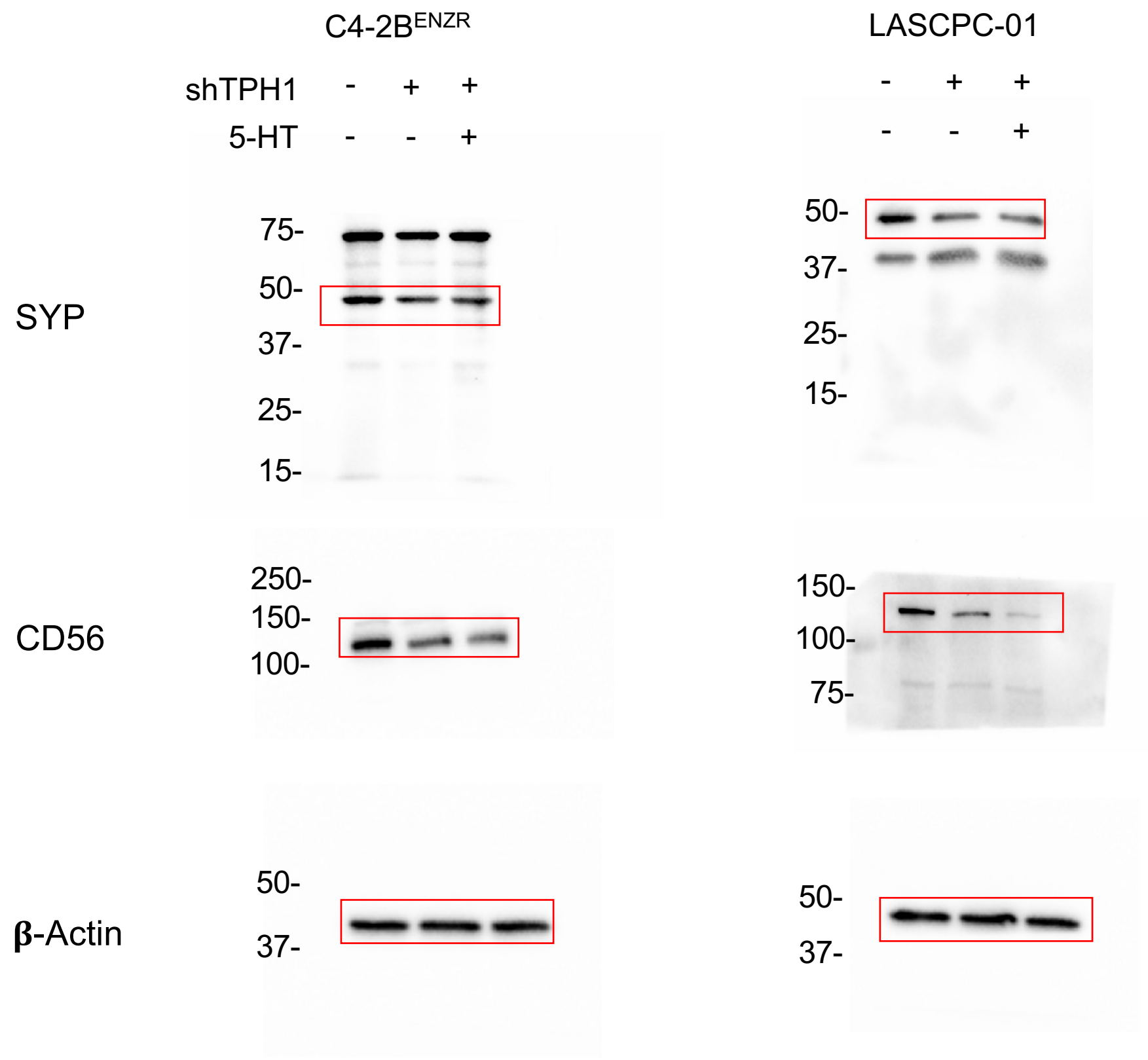

Full unedited blots for Supplemental Figure 8C

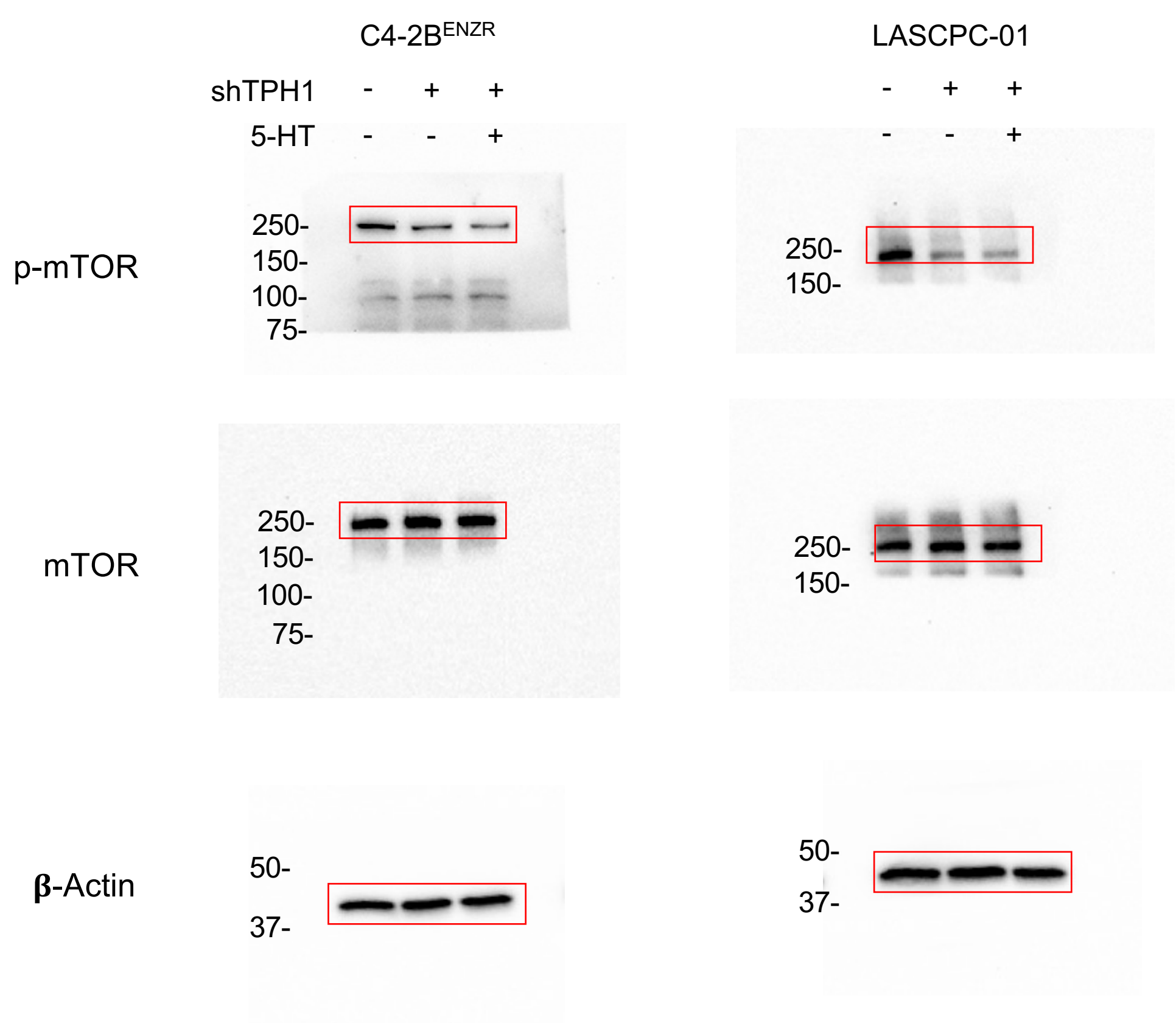

Full unedited blots for Supplemental Figure 8E

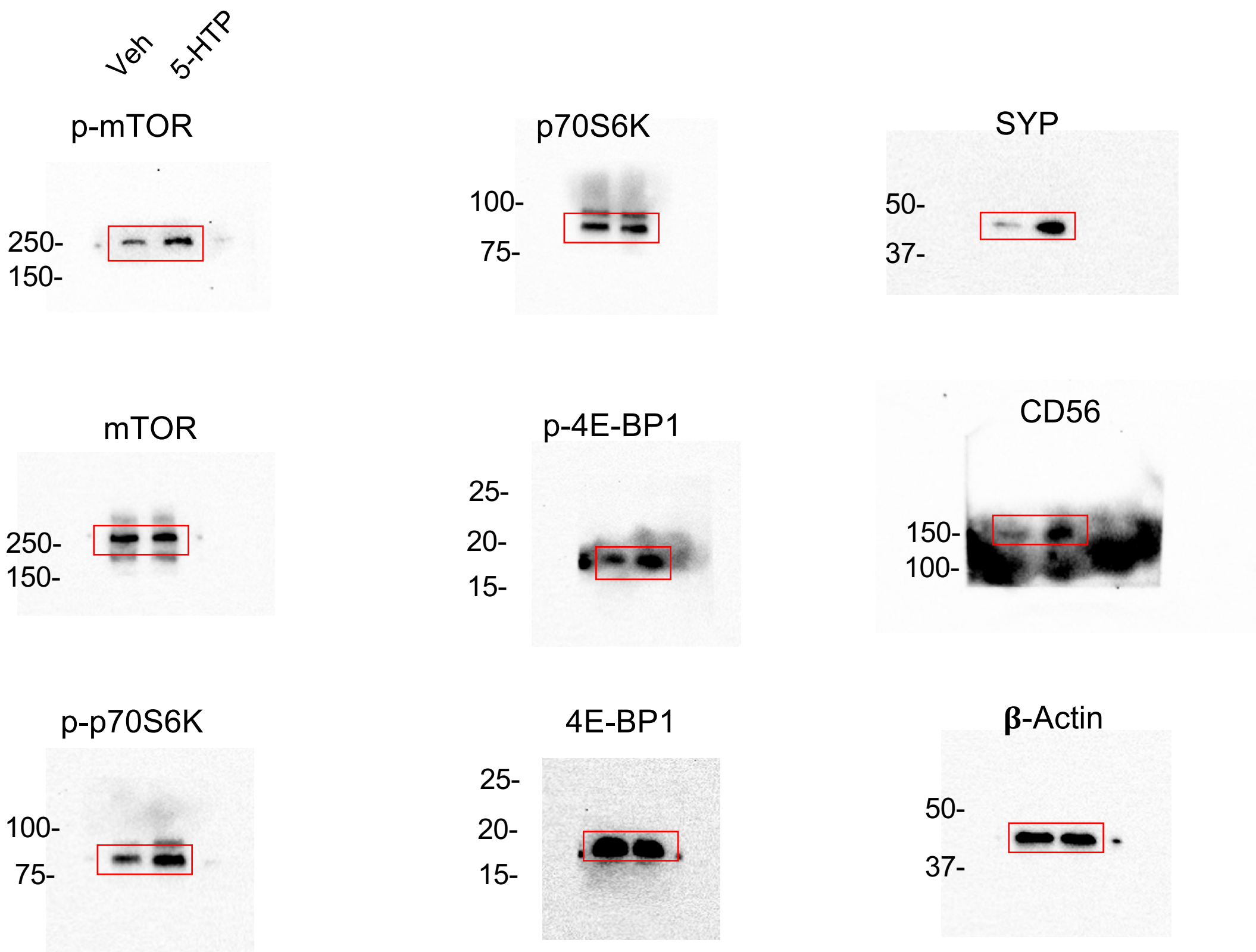

Full unedited blots for Supplemental Figure 8F

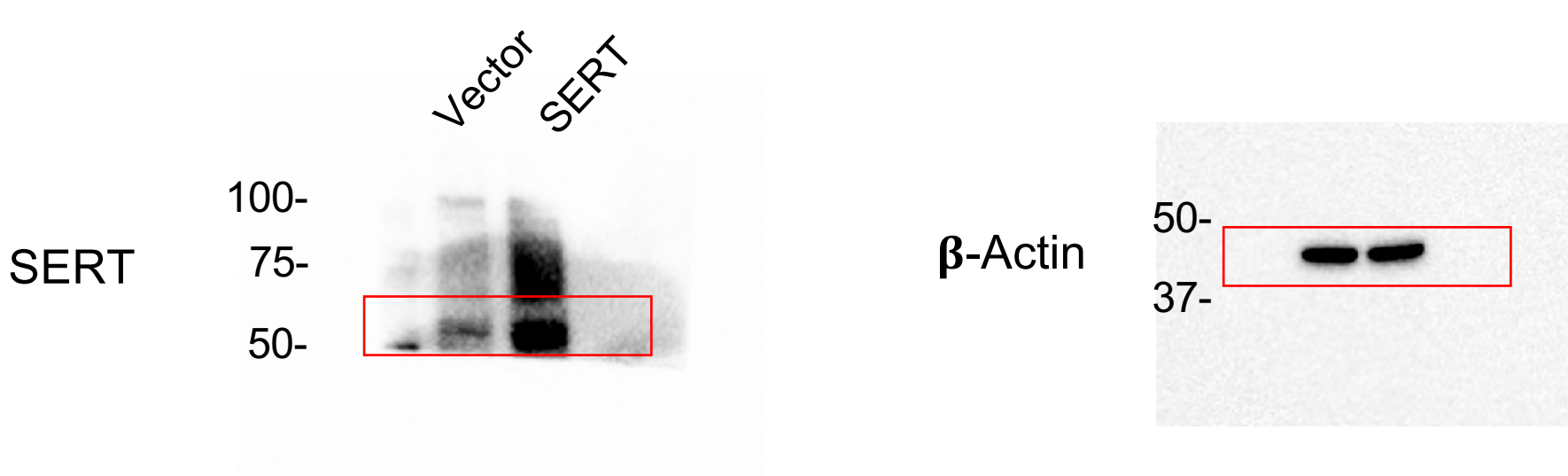

Full unedited blots for Supplemental Figure 9B

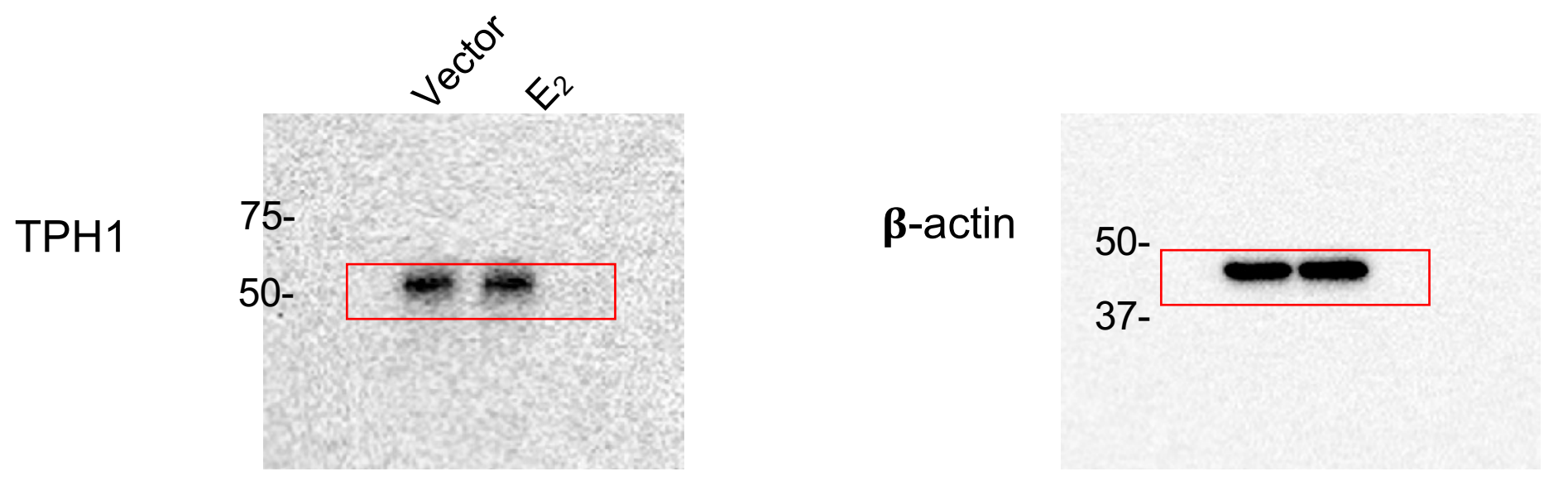

Full unedited blots for Supplemental Figure 9M

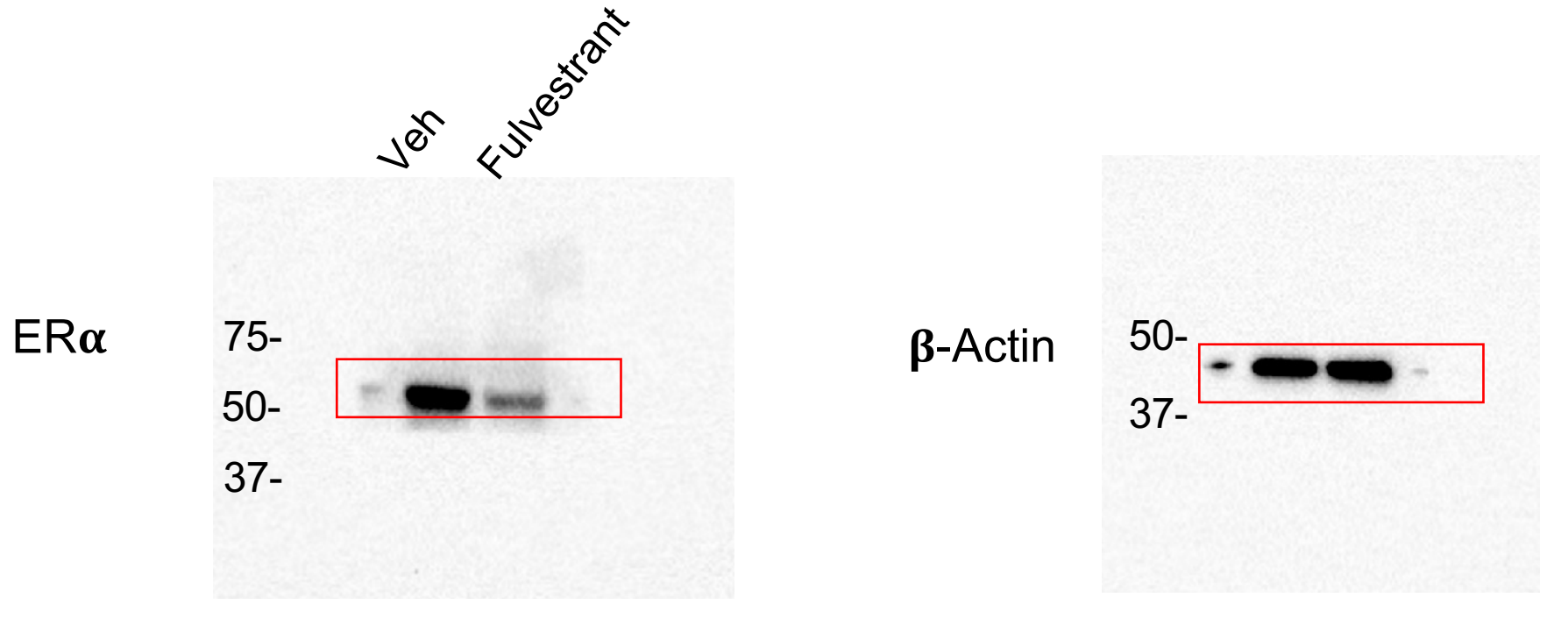

Full unedited blots for Supplemental Figure 9N

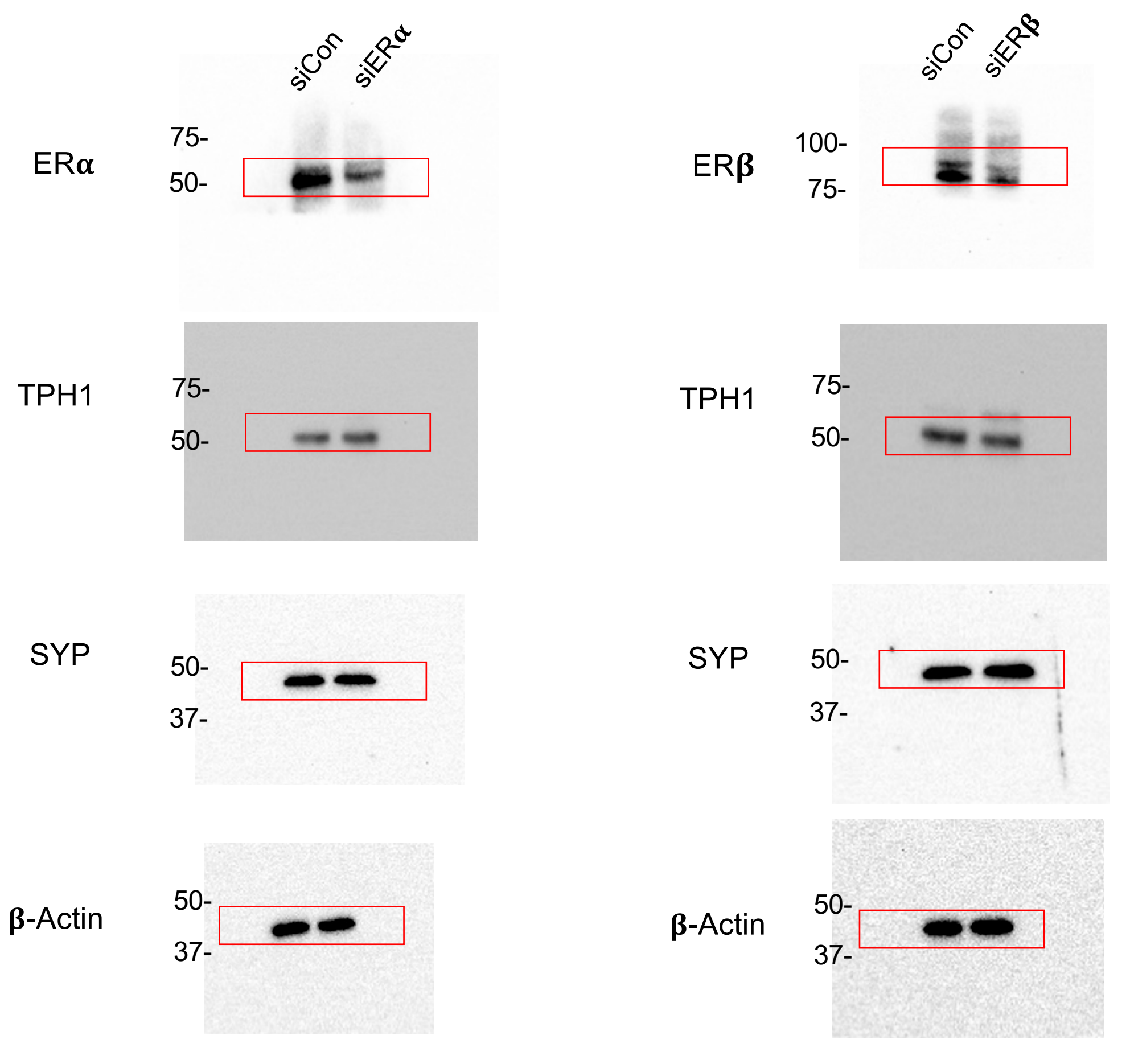

Full unedited blots for Supplemental Figure 10D

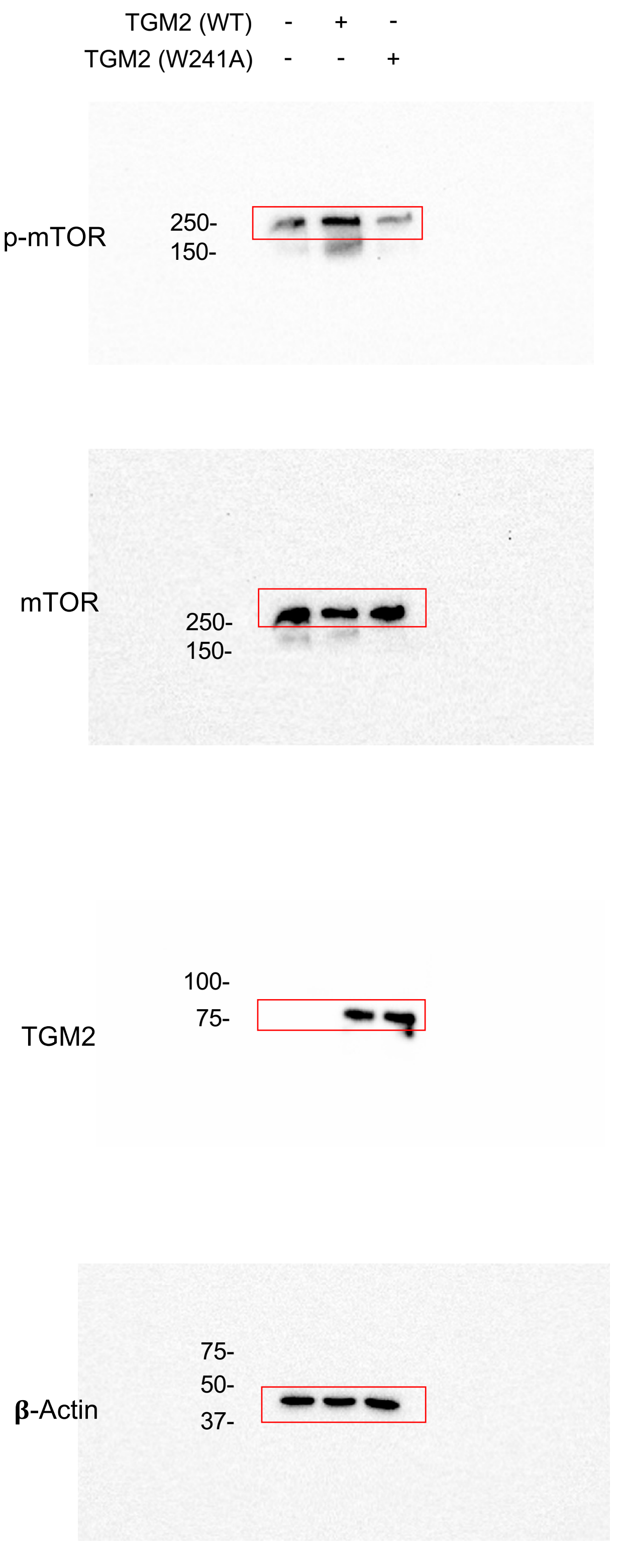

Full unedited blots for Supplemental Figure 10E

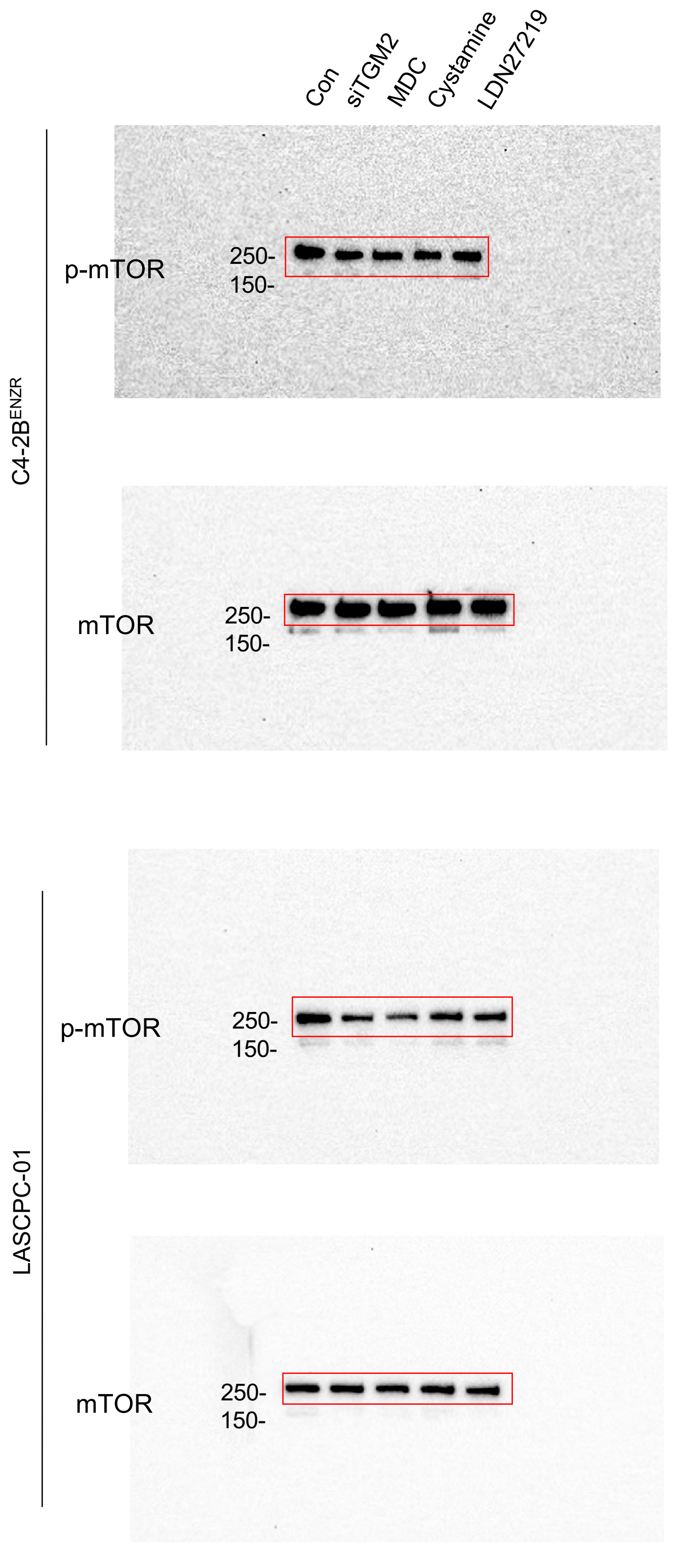

Full unedited blots for Supplemental Figure 11B

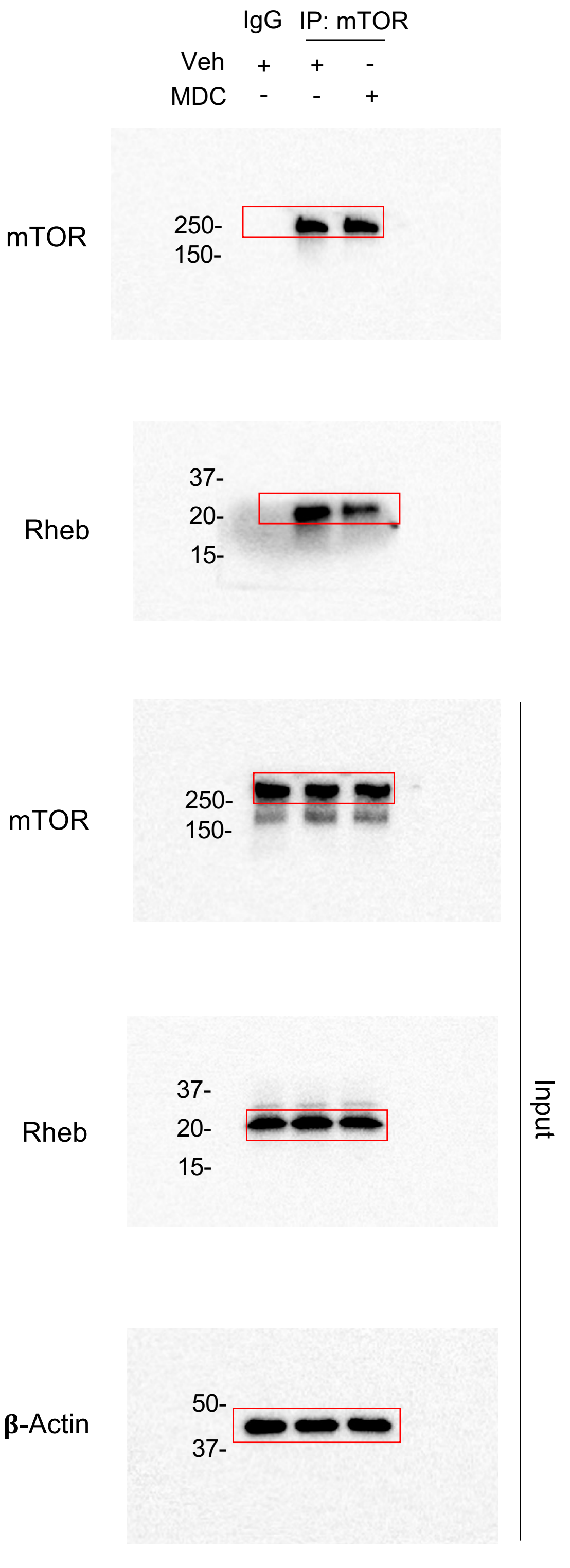

Full unedited blots for Supplemental Figure 11D

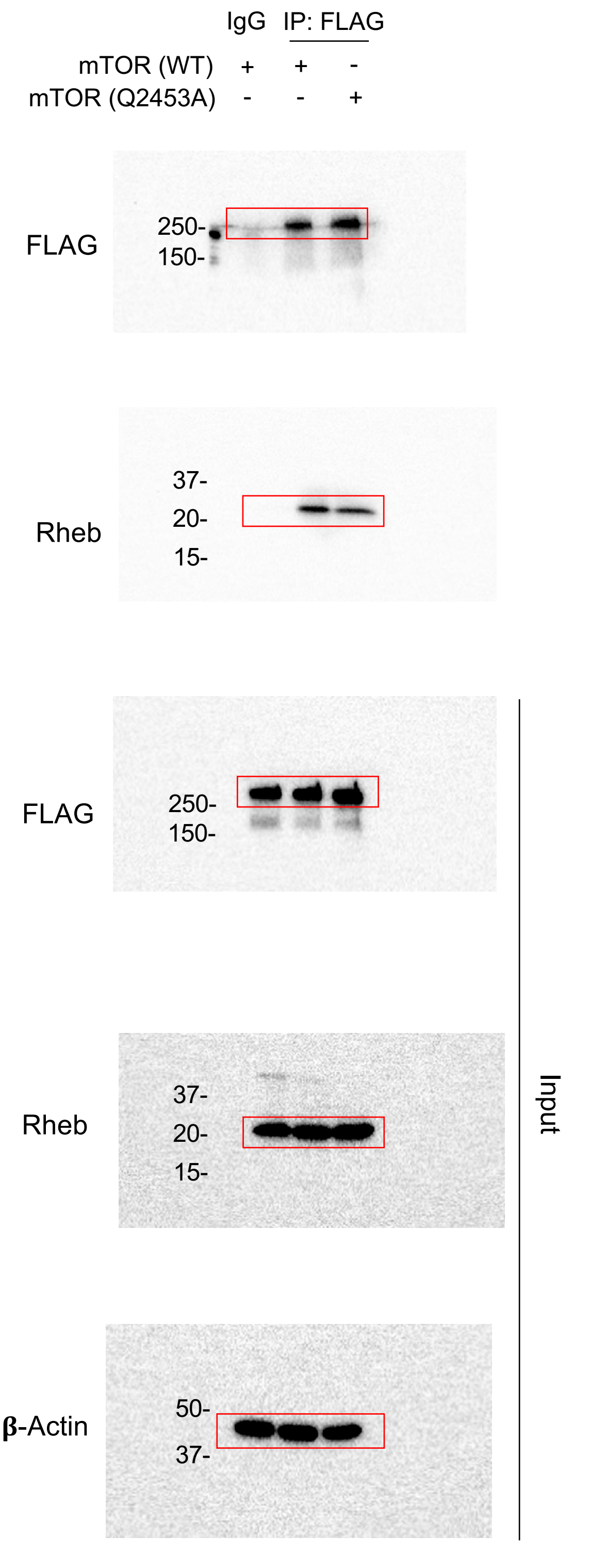

Full unedited blots for Supplemental Figure 12A

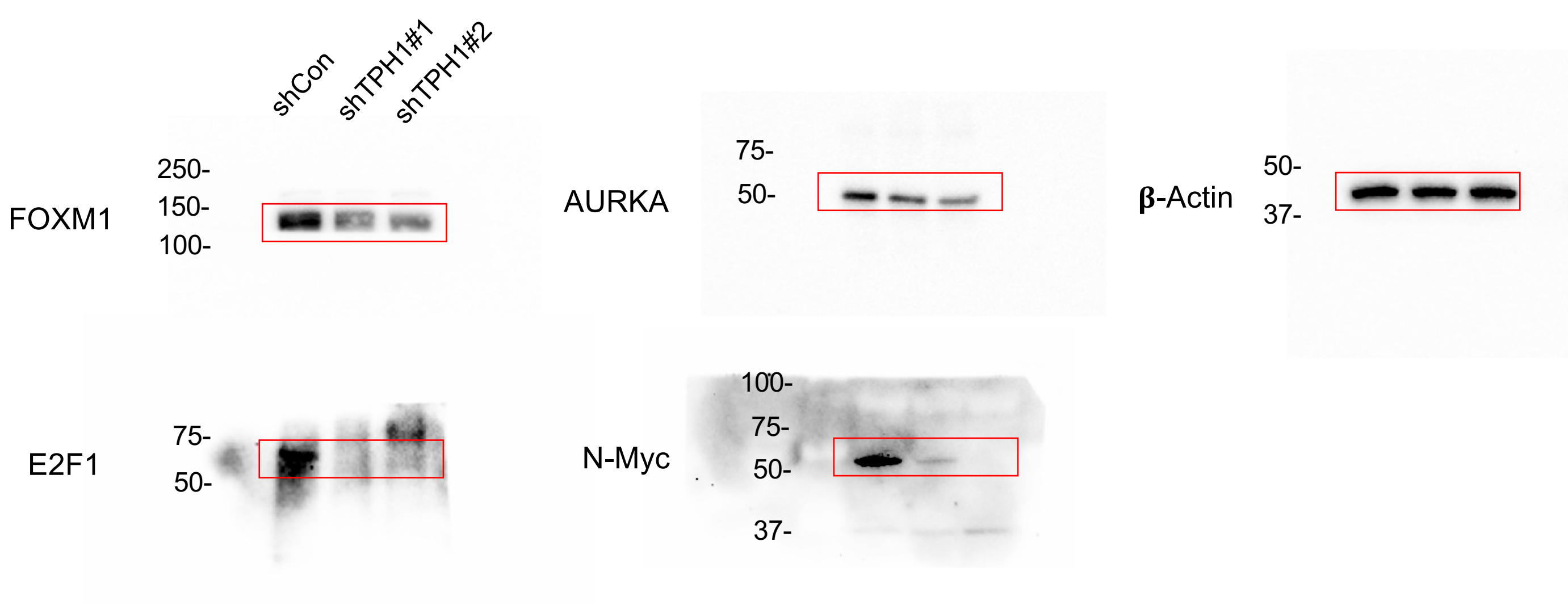

Full unedited blots for Supplemental Figure 12C

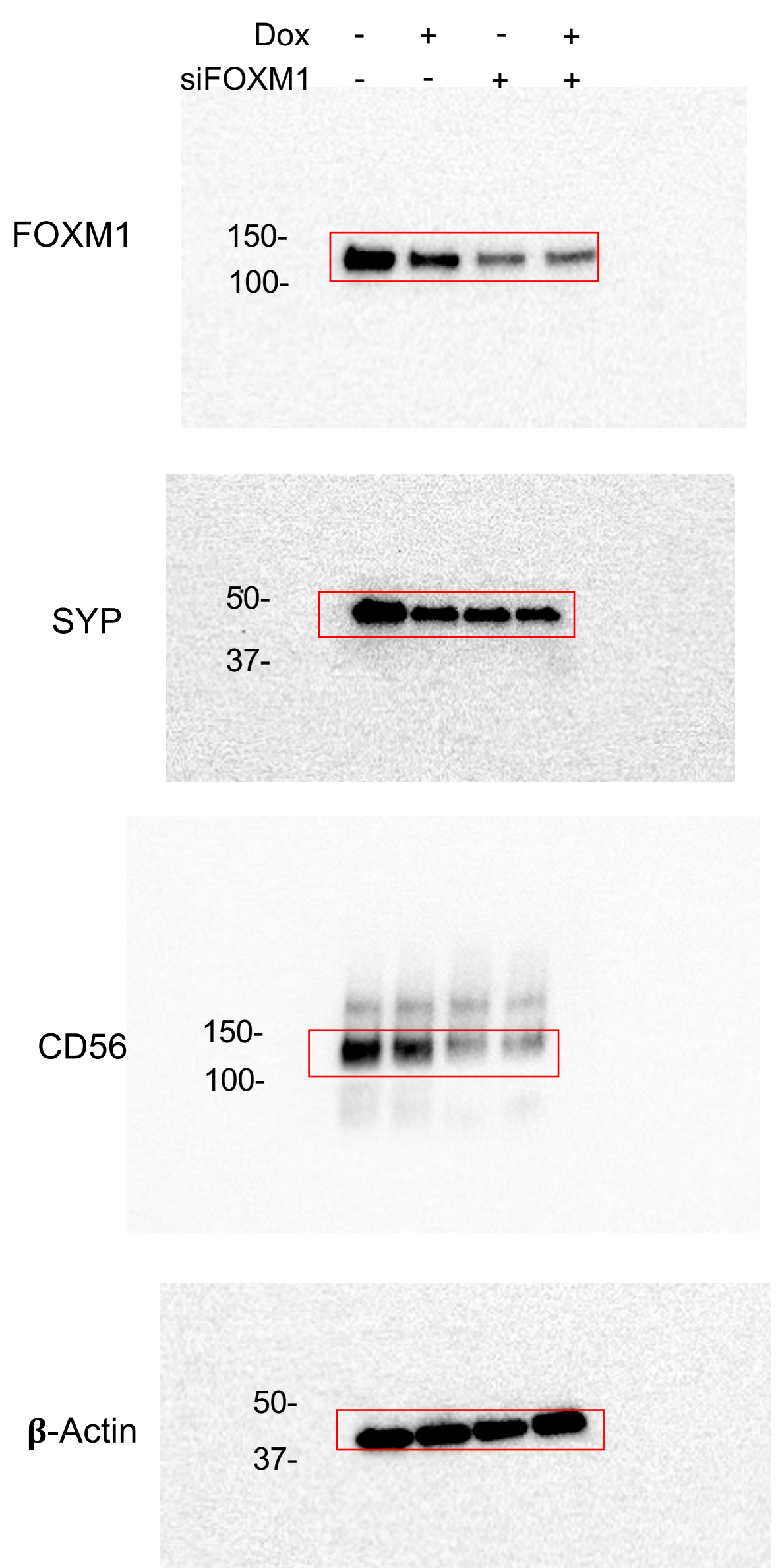

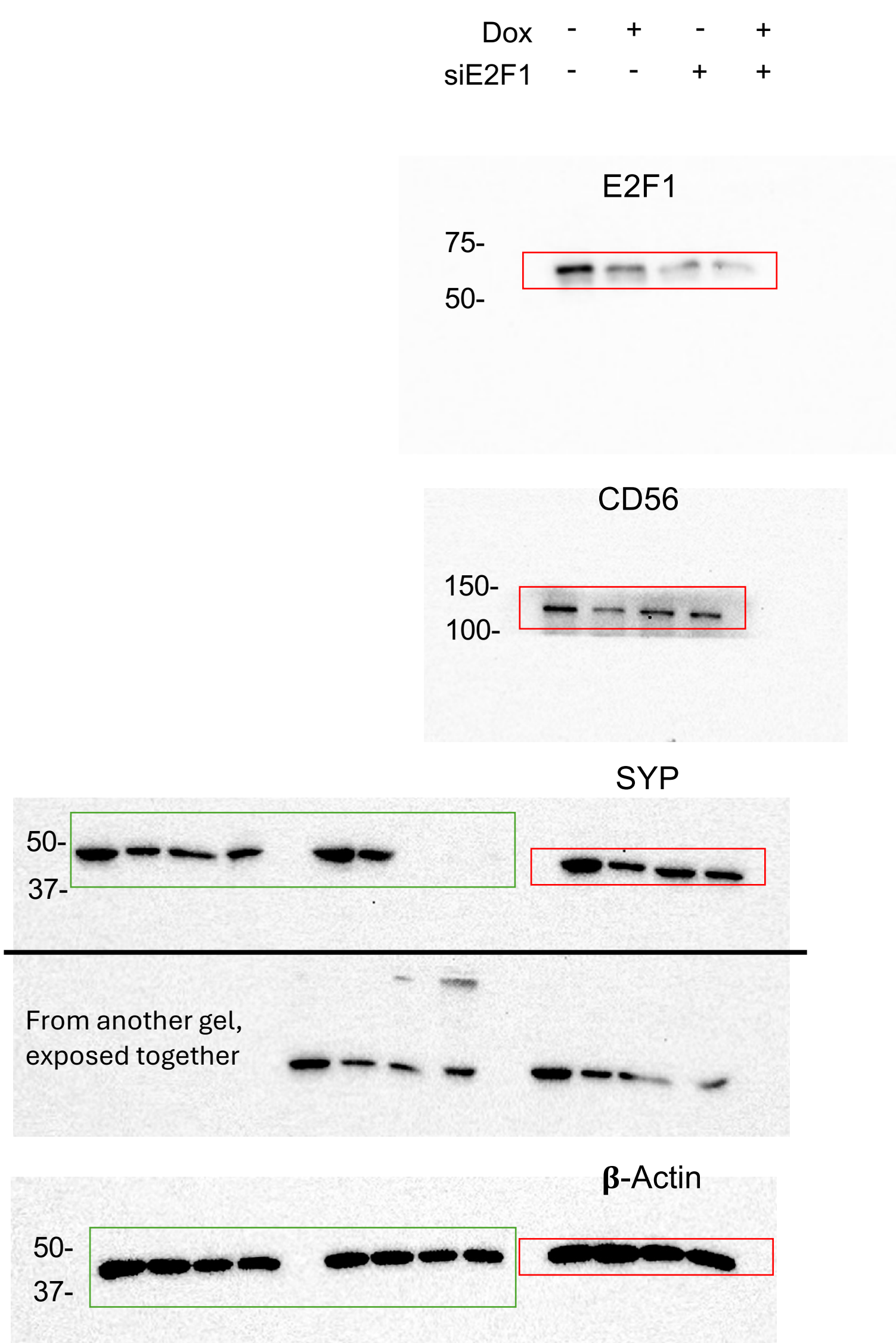

Annotation:
Red box: Results used in this experiment.
Green box: Samples run on the same gel but not related to this experiment.
Black line: Boundary between different gels.

Full unedited blots for Supplemental Figure 13B

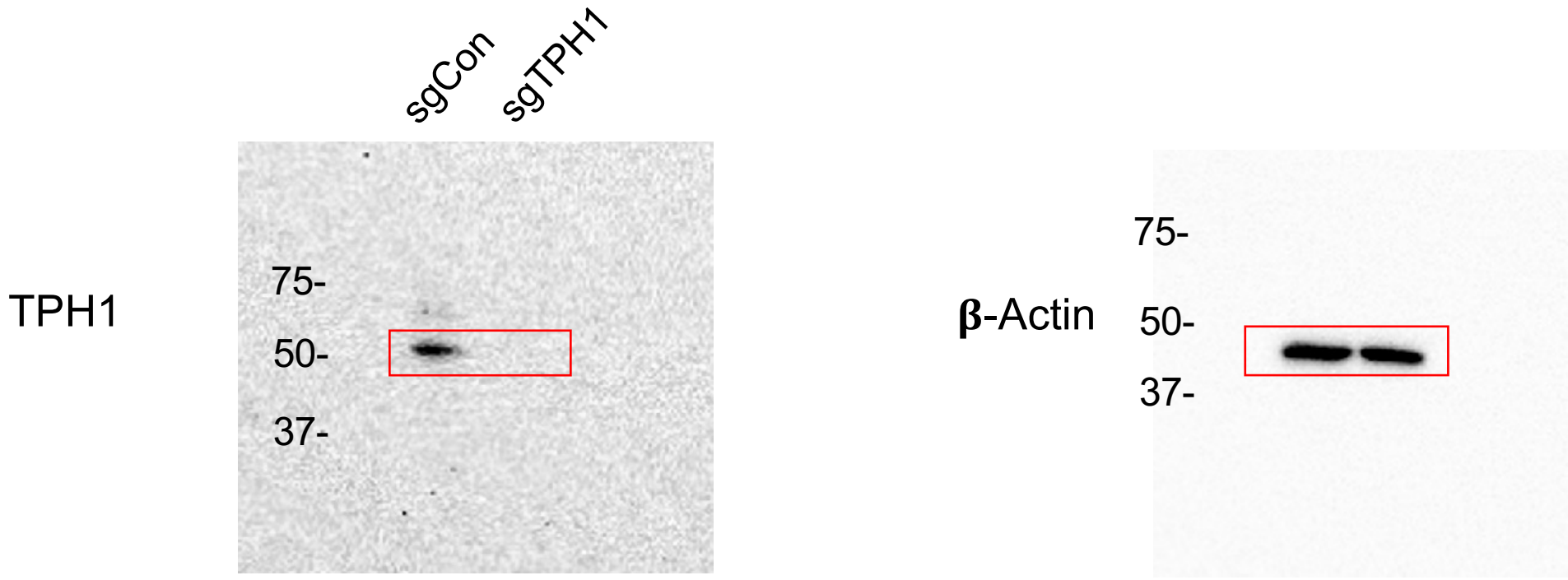

Full unedited blots for Supplemental Figure 13D

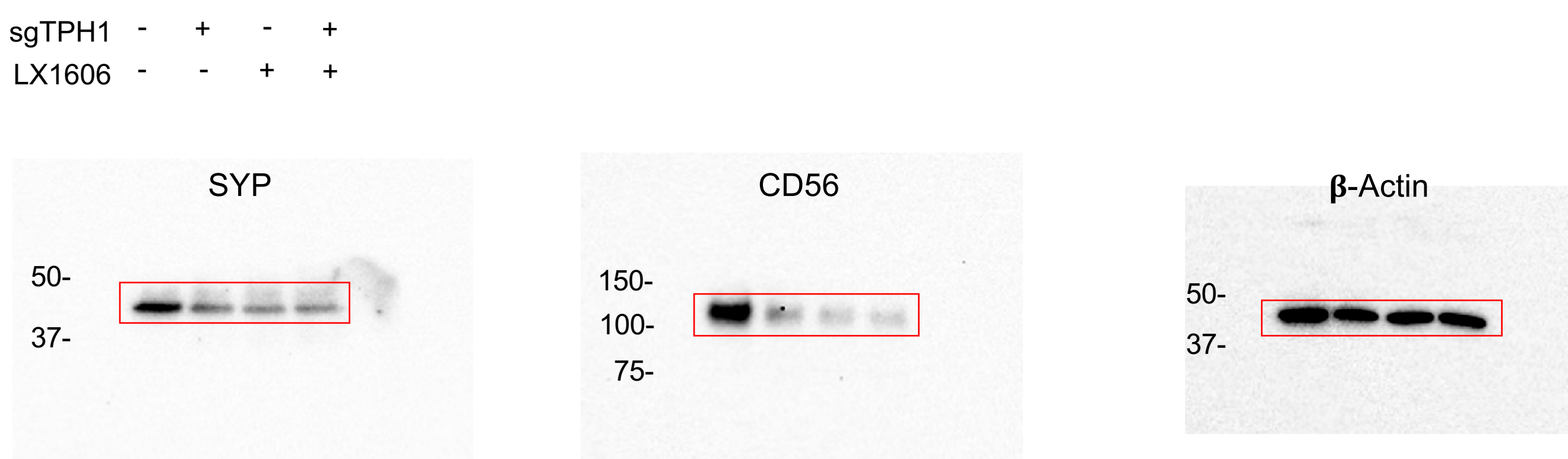

Full unedited blots for Supplemental Figure 13K

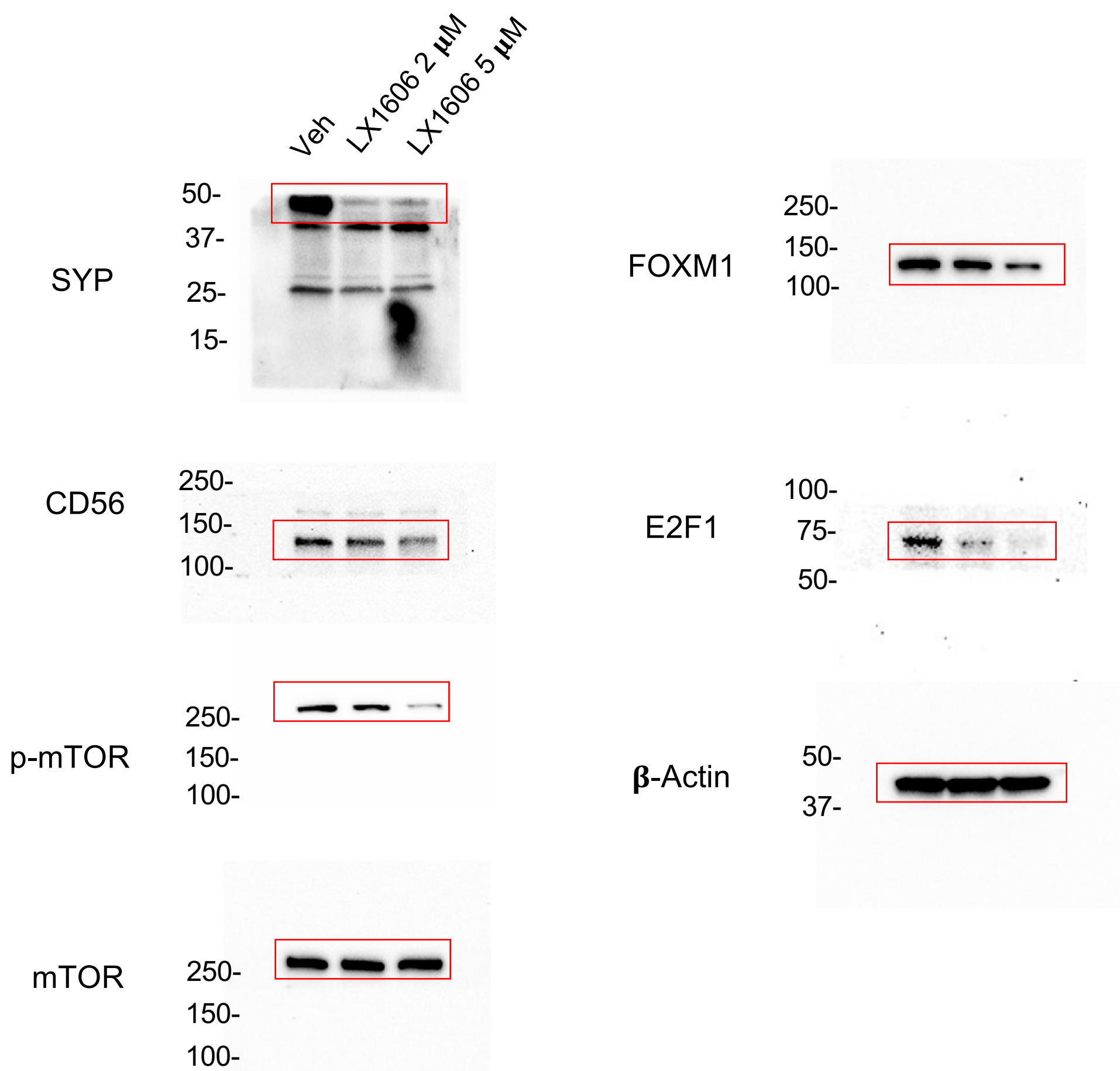

Full unedited blots for Supplemental Figure 18A

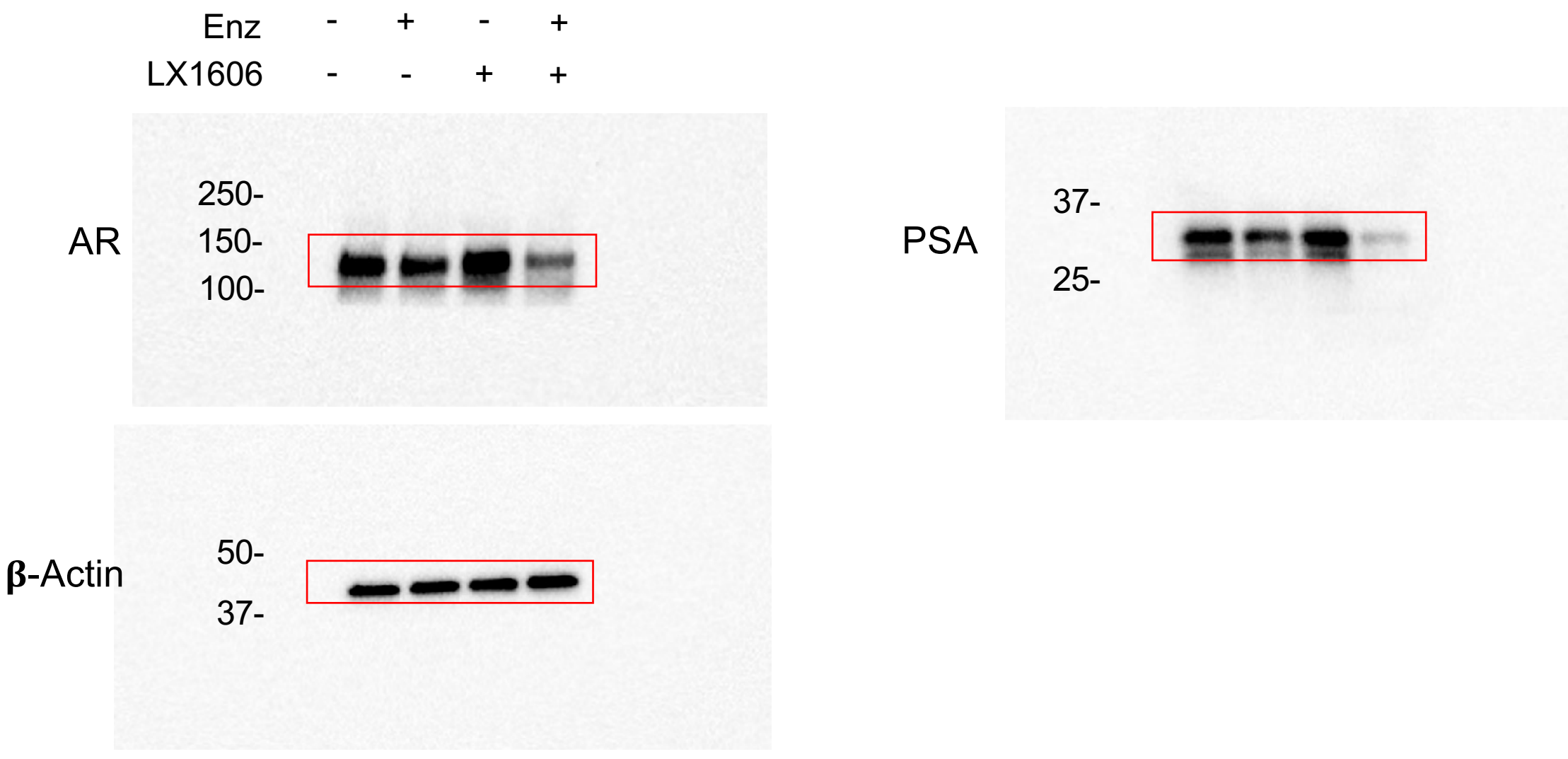

Supplement: Supplementary file 2 [file jci-136-200391-s011.pdf]
